# Design and synthesis of 3-(1*H*-indol-3-yl)-*N*-(3,4,5-trimethoxyphenyl) acrylamide derivatives as anticancer agents

**DOI:** 10.1039/d6ra03896d

**Published:** 2026-10-22

**Authors:** Seyedeh Niloufar Ghafouri, Parviz Rashidi Ranjbar, Mohammad Mahdavi, Aida Iraji

**Affiliations:** a School of Chemistry, College of Science, University of Tehran Tehran Iran parvizrashidi2@ut.ac.ir; b Endocrinology and Metabolism Research Center, Endocrinology and Metabolism Clinical Sciences Institute, Tehran University of Medical Sciences Tehran Iran; c Stem Cells Technology Research Center, Shiraz University of Medical Sciences Shiraz Iran aida.iraji@gmail.com iraji@sums.ac.ir; d Pharmaceutical Sciences Research Center, Shiraz University of Medical Sciences Shiraz Iran

## Abstract

In this study, a new series of indole-based trimethoxy aniline hybrids (10a–o) was designed through a fragment-based hybridization strategy and synthesized *via* a multistep synthetic procedure. The cytotoxicity of the synthesized compounds was evaluated against human breast cancer cell lines (MCF-7 and MDA-MB-468), hepatocellular carcinoma cells (Hep-G2), and the non-cancerous HEK-293 cell line using MTT assay. Several analogs exhibited moderate antiproliferative activity, and compound 10b, bearing an *ortho*-methyl substituent, emerged as the most active derivative against MCF-7. Structure–activity relationship (SAR) analysis revealed that *ortho*-alkyl substituents favored selectivity toward MCF-7 cells, whereas 4-Br substitution and ethyl-alkyl extension preferentially enhanced activity against Hep-G2 cells. Mechanistic studies demonstrated that 10b primarily induced apoptotic cell death at lower concentrations and exposure times, whereas higher concentrations and prolonged treatment resulted in more necrotic cell death. Compound 10b also induced dose-dependent alterations in cell-cycle progression in MCF-7 cells, characterized by S-phase arrest.

## Abstract 2

In this study, a new series of indole-based trimethoxy aniline hybrids (10a–o) was designed through a fragment-based hybridization strategy and synthesized *via* a multistep synthetic procedure.

## Graphical abstract

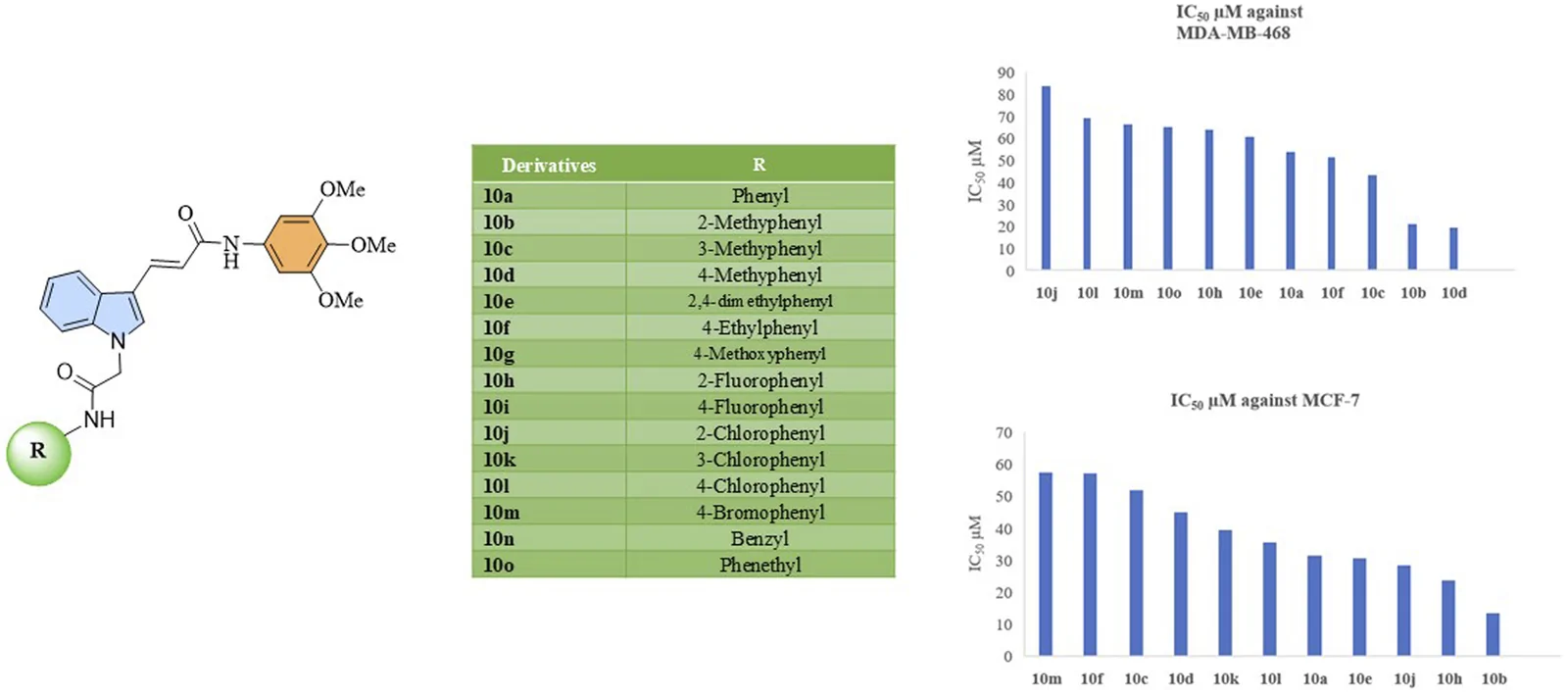

## 1. Introduction

Cancer remains one of the most serious health concerns in the 21st century, ranking as the second leading cause of death globally.^1^ Cancer is a complex disease characterized by deregulated growth and uncontrolled loss of normal cellular response.^2^ Although impressive progress has been made in therapeutic intervention, including chemotherapy, radiotherapy, and immunotherapy, the drug resistance, high toxicity, and insensitivity of some cancer types still prevent an ideal treatment.^3^

Among various cancer types, breast cancer remains the most frequently diagnosed malignancy among women worldwide and a leading cause of cancer-related mortality.^4^ Despite significant progress in targeted and endocrine therapies, treatment of aggressive and drug-resistant subtypes, particularly triple-negative breast cancer (TNBC), remains a major clinical challenge due to the lack of effective molecular targets and the rapid emergence of chemoresistance. Consequently, the development of novel drugs with lower toxicity is of importance.^5^

Heterocyclic compounds, especially indole analogs, have attracted considerable interest in pharmacological studies owing to their potent anticancer activity.^6^ For instance, Sunitinib (Fig. 1), as a tyrosine kinase inhibitor with an indole structure, is approved for the treatment of renal cancer and gastrointestinal tumors. Nintedanib (Fig. 1) is used in the treatment of triple-negative breast cancer.^7^ These examples demonstrate the potential of indole compounds in developing anticancer drugs. Numerous studies have demonstrated that indole derivatives possess significant anticancer activity; 2,3-dimethylindoles (compound A) (Fig. 1) exhibited significant anticancer potency against various cell lines, such as ACHN, Panc1, CaIu1, and H460, with IC_50_ values of 3.7, 2.8, 2.7, and 3.2, respectively.^8^ Moreover, compound 5-fluoro-2,3-dimethyl-1*H*-indole (compound B) (Fig. 1) showed excellent potency against three cell lines (ACHN, Panc1, Calu1) with IC_50_ values of 3.8, 3.2, and 3.1, respectively.^8^ Notably, the presence of polar groups in these compounds plays a critical role in enhancing efficacy. Indole-triazole conjugates (compound C) (Fig. 1) exhibited significant inhibition of tubulin polymerization, with 63 and 66% of cells arrested in the G2/M phase in comparison to control cells (10%). The tubulin polymerization inhibition assay revealed that (compound C) had a significant inhibitory effect, with an IC_50_ value of 2.31 µM.^9^ Additionally, (compound D) (Fig. 1) exhibited a promising IC_50_ value of 8.5 µM against the MCF-7 cell line.^10^

**Fig. 1:**
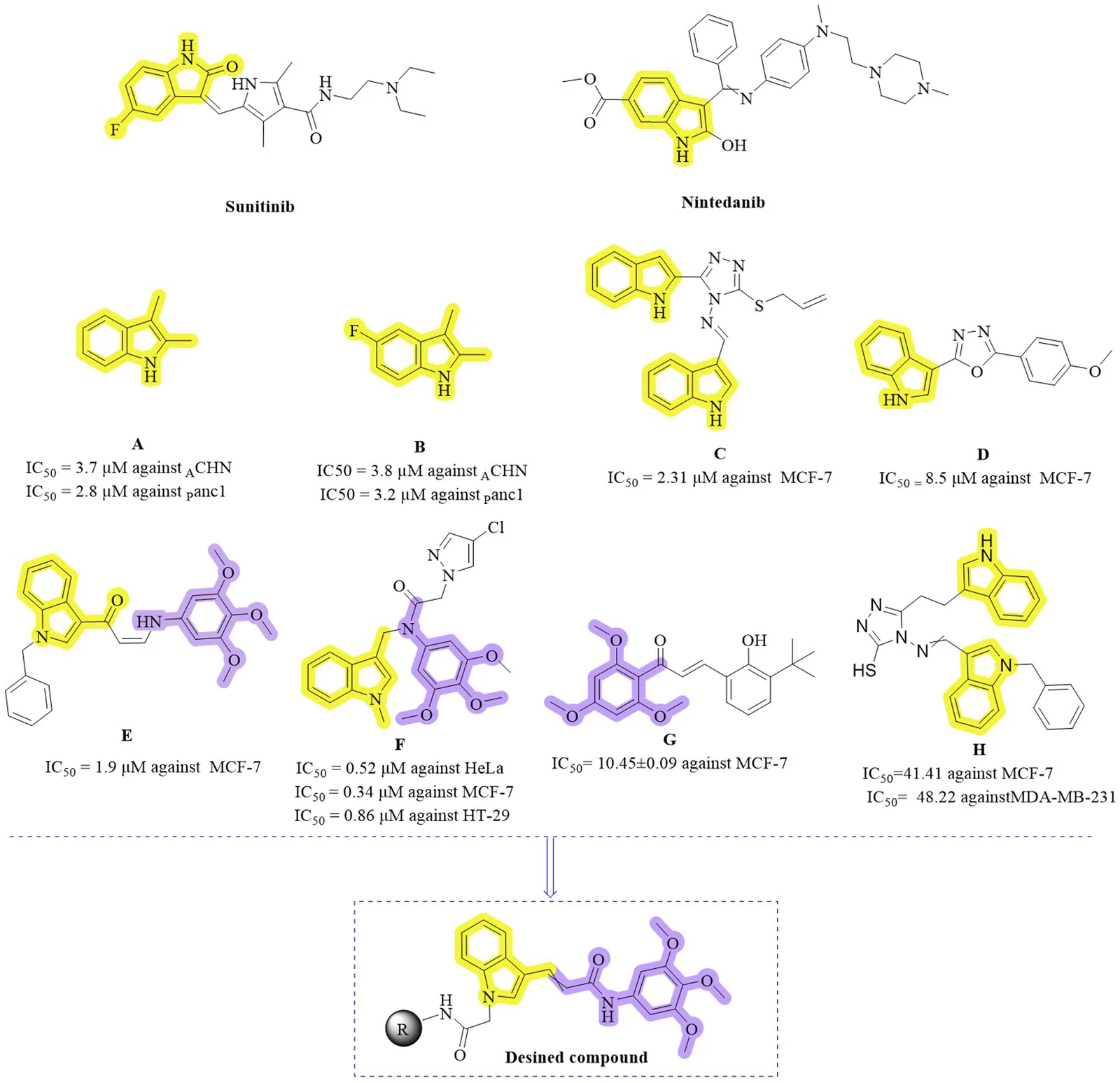
Design of indole derivatives bearing a 3,4,5-trimethoxyphenylacrylamide moiety based on previously reported anticancer agents (A–H).

In another study, Shareef *et al.* demonstrated that incorporation of the 3,4,5-trimethoxyphenyl group into the indolyl backbone in (compound E) (Fig. 1) significantly improved the potency to IC_50_ = 1.9 µM against the MCF-7.^11^ Furthermore, indol-3,4,5-trimethoxyphenylacetamides (compound F) (Fig. 1) were evaluated against HeLa, MCF-7, and HT-29 cancer cell lines, and the most potent analog showed activity comparable to the positive control, colchicine. Importantly, this compound inhibited tubulin polymerization similar to colchicine.^12^ Investigation of chalcone-based derivatives showed that the presence of trimethoxy groups improved cytotoxicity against breast cancer, with IC_50_ values ranging from 10 to 20 µM (compound G) (Fig. 1).^13^ Additionally, new indole-linked 1,2,4-triazole derivatives were investigated as anti-TNBC agents; and the IC_50_ values indicate that the synthesized compounds show promising inhibition against MCF-7 cells and MDA-MB-231 cells, ranging from 41 to 77 µg mL^−1^. Moreover, compound H exhibited an inhibitory effect relative to doxorubicin, the reference drug.^14^

Considering these findings, the present study developed a fragment-based molecular hybridization strategy to design a new series of indole-trimethoxyphenyl conjugates. First, the indole scaffold was retained as the central core owing to its well-documented anticancer activity. Second, the 3,4,5-trimethoxy aniline group was incorporated as it might engage in hydrophobic and hydrogen-bonding interactions with the key residues of the possible target. Third, an acrylamide linker was selected to connect these two pharmacophoric units, as a conformationally extended amide-containing linker. Systematic variation of substituents on the terminal aniline ring was then introduced to explore the influence of steric, electronic, and lipophilic properties on cytotoxic activity and cell line selectivity.

The anticancer activity of all synthesized derivatives was evaluated against MCF-7, MDA-MB-468, and Hep-G2 cancer cell lines, as well as the non-cancerous HEK-293 cell line, to assess selectivity for malignant cells. The breast cancer cell lines were selected for their clinical relevance and the reported sensitivity of breast tumors to indole-trimethoxy aniline-based compounds. In addition, Hep-G2 cells were also included because hepatocellular carcinoma is a primary liver malignancy and remains one of the leading causes of cancer-related mortality worldwide, with limited therapeutic options.^15^ The most potent analog was further characterized at different concentrations and times of exposure to determine its effects on apoptosis and cell-cycle progression.

## 2. Results and discussion

### 2.1 Chemistry

To synthesize indole-3-carboxaldehyde (2), phosphorus oxychloride (340 mmol, 32.0 mL) was added dropwise to DMF (50 mL) at 0–5 °C, and the mixture was stirred in an ice bath for 1 h. Then, indole (1) (170 mmol, 20 g, dissolved in 15 mL of DMF) was added to the latter ice bath, and the reaction was continued at 25 °C for 2 h. After that, the mixture was poured into ice water (600 mL), and NaOH was added to adjust the pH to 8.0. The crude product was filtered off and washed with water to afford indole-3-carboxaldehyde (2).^16^

To synthesize 2-chloro-*N*-phenylacetamide derivatives (5a–o), a mixture of aniline derivatives (3a–o) (2 mmol) and chloroacetyl chloride (4) (2 mmol) in DMF (10 mL) was stirred at room temperature for 24 h. The reaction mixture was then diluted with cold water and poured onto ice. The resulting precipitate was filtered, washed with cold water, and dried to afford pure *N*-phenyl-2-chloroacetamides (5a–o).^17^ To synthesize (*E*)-2-(3-(3-hydroxyprop-1-en-1-yl)-1*H*-indol-1-yl)-*N*-phenylacetamide derivatives (8a–o), a mixture of indole-3-carboxaldehyde (2) (1 mmol), 2-chloro-*N*-substituted acetamide derivatives (5a–o) (1.2 mmol) in dry acetone (10 mL) and anhydrous K_2_CO_3_ (1.2 mmol) was stirred at room temperature for 4 h, filtered and the solid product formed was crystallized in ethanol to give final products (6a–o).^18^ A mixture of malonic acid (7) (12 mmol), aromatic aldehydes (6a–o) (10 mmol), piperidine (15 mmol), and pyridine (0.3 mmol) was stirred and refluxed for about 2–3 h and monitored by TLC. After removal of the solvent under vacuum, the residue was poured into ice water, and the pH was adjusted to 2 with 10% HCl. Then, the precipitate was filtered, washed well with H_2_O, recrystallized from 95% alcohol, and dried under vacuum to afford the cinnamic acid analogs (8a–o).^17,19^ To synthesize 3-(1-(2-oxo-2-(phenylamino) ethyl)-1*H*-indol-3-yl)-*N*-(3,4,5-trimethoxyphenyl) acrylamide derivatives (10a–o), in an ice bath, compound (8a–o) (1 mmol) was dissolved in 5 mL of DMF. Diisopropylethylamine (DIEA, 2.0 equivalents) was then added, and the resulting solution was stirred for 1 hour. Subsequently, TBTU ([benzotriazol-1-yloxy(dimethylamino)methylidene]-dimethylazanium; tetrafluoroborate) (1.4 mmol) was introduced, and after an additional hour of stirring, 3,4,5-trimethoxyaniline (1.1 mmol) was added to the reaction mixture. The progress of the reaction was monitored *via* thin-layer chromatography (TLC). Upon completion of the reaction, the mixture was cooled and diluted with water, yielding the precipitated product. The solid was isolated by filtration and washed several times with cold water, yielding the desired product (10a–o)^20,21^ (Scheme 1).

**Scheme 1:**
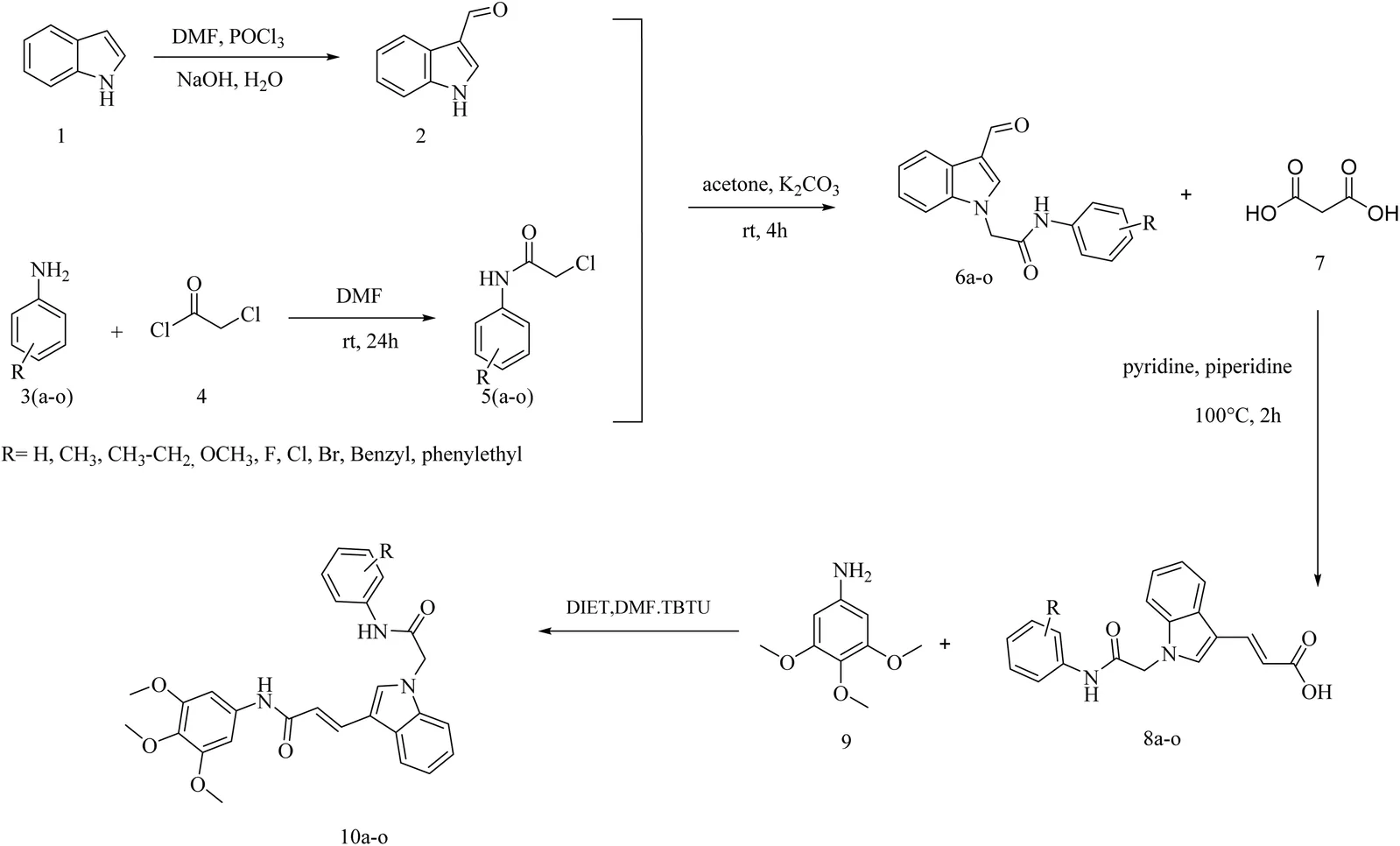
The pathway for synthesis of 10a–o.

### 2.2 Cytotoxicity assay

To establish the structure–activity relationships (SAR) within this series, the cytotoxic activities of compounds 10a–o were analyzed by considering the effects of substituent type, substitution pattern, steric properties, electronic characteristics, and calculated physicochemical descriptors (*c* Log *P* and TPSA). Although the compounds exhibited differences in cytotoxicity, the calculated *c* Log *P* (5.49–6.36) and TPSA (96.14–106.26 Å2) values were only slightly different; therefore, the observed biological differences are not expected to be explained solely by physicochemical properties. The substituent type, size, and electronic properties might have a significant role in the cytotoxic potency.

Analog 10a (Table 1 and Fig. 2) was selected as a reference compound as it exhibited moderate cytotoxicity against both breast cancer cell lines (MCF-7 and MDA-MB-468) and was inactive against Hep-G2 cells. The introduction of alkyl substituents led to pronounced position-dependent effects. Among these derivatives, the *ortho*-methyl analog (10b) was found to be one of the most active compounds of the series with IC_50_ values of 13.52 µM against MCF-7 and 21.15 µM against MDA-MB-468, which corresponds to about 2.5- and 1.5-fold enhancement over the parent compound 10a, respectively. Conversely, the methyl substituent in the *meta* (10c) or *para* (10d) position reduced activity against one or both of the breast cancer cell lines, whereas the introduction of a second methyl substituent (10e) or its replacement by an ethyl group (10f) did not lead to any further enhancement. Since these alkyl derivatives possess highly comparable *c* Log *P* (6.09–6.36) and TPSA (96.14–98.42 Å^2^) values, the differences in biological activity cannot be attributed solely to variations in lipophilicity or molecular polarity. Instead, the results suggest that the positional arrangement and steric orientation of a small hydrophobic substituent at the *ortho* position are more favorable for activity.

**Table 1:** Cytotoxicity assessments of 10a–o^a^^,^^c^

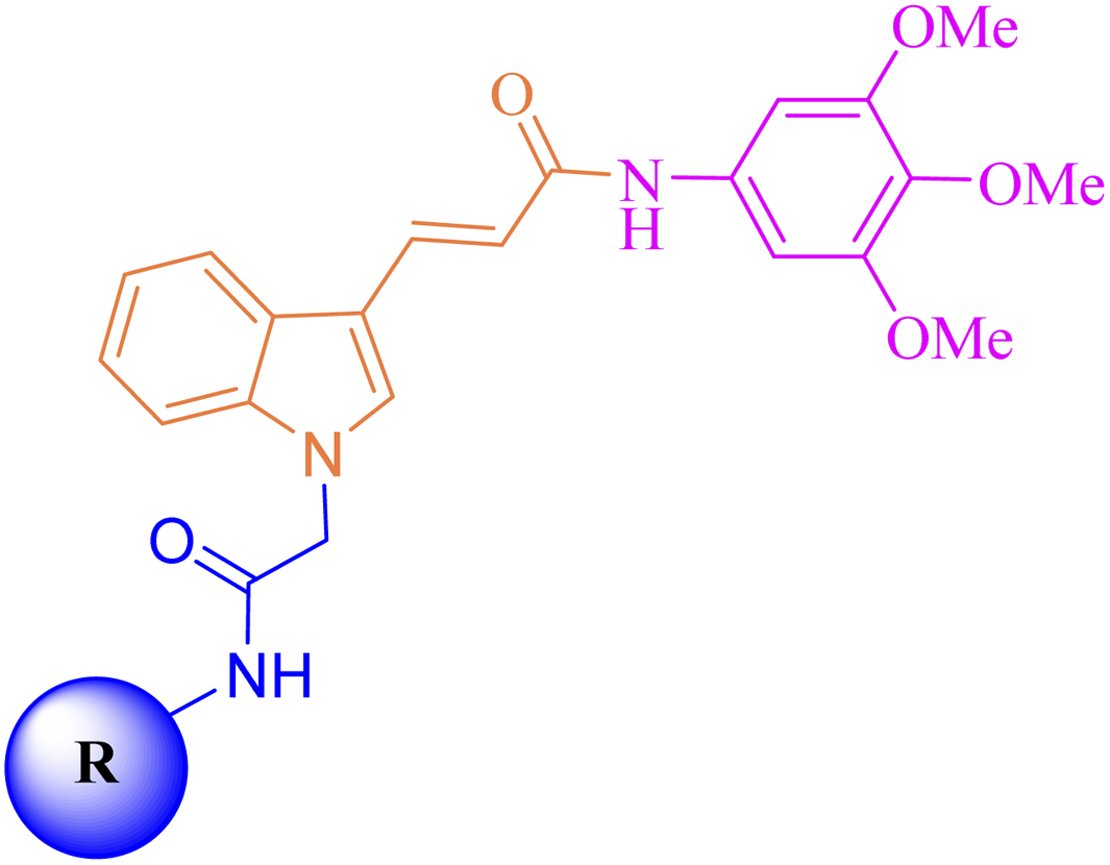							
Code	R	IC_50_ µM against MDA-MB-468	IC_50_ µM against MCF-7	IC_50_ µM against Hep-G2	IC_50_ µM Hek-293	Log *P*	TPSA
10a	Phenyl	53.69 ± 3.40	31.51 ± 3.46	>100**	>100	5.78	98.41
10b	2-Methyphenyl	21.15 ± 2.21**	13.52 ± 2.00*	58.00 ± 5.87**	90.82 ± 10.38**	6.13	96.33
10c	3-Methyphenyl	43.33 ± 2.52	51.80 ± 8.80*	>100	>100	6.10	97.32
10d	4-Methyphenyl	19.67 ± 0.46**	44.82 ± 2.47*	>100	>100	6.091	98.42
10e	2,4-Dimethylphenyl	60.55 ± 1.96**	30.47 ± 2.43	>100	88.8 ± 6.87**	6.101	96.14
10f	4-Ethylphenyl	51.21 ± 2.71	56.99 ± 1.82*	>100	>100	6.357	97.84
10g	4-Methoxyphenyl	>100	>100	>100	>100	5.782	106.26
10h	2-Fluorophenyl	63.77 ± 8.15**	23.64 ± 1.19	43.58 ± 5.48**	>100	6.012	97.76
10i	4-Fluorophenyl	>100	>100*	>100	>100	6.015	98.41
10j	2-Chlorophenyl	83.73 ± 7.69**	28.34 ± 4.77	41.26 ± 9.67**	>100	6.253	97.31
10k	3-Chlorophenyl	>100	39.32 ± 6.70*	36.19 ± 8.45	>100	6.277	98.36
10l	4-Chlorophenyl	69.22 ± 2.90**	35.48 ± 5.22*	>100	>100	6.272	98.42
10m	4-Bromophenyl	66.24 ± 6.52**	57.38 ± 11.39*	21.53 ± 1.49	43.86 ± 8.30**	6.348	98.42
10n	Benzyl	>100	>100	>100	>100	5.487	99.55
10o	Phenethyl	65.06 ± 0.74**	>100	15.79 ± 2.78**	>100	5.853	100.36
Cisplatine^b^		45.81 ± 9.65	28.30 ± 0.91	31.14 ± 1.29	11.87 ± 0.27	—	—

a Mean ± standard deviation of three independent experiments.

b Positive control.

c *P < 0.05 *vs.* the cisplatin-treated group (one-way ANOVA followed by Dunnett's post hoc test) and IC_50_ > 100 µM was eliminated from the assessments. **P < 0.0001 *vs.* the cisplatin-treated group (one-way ANOVA followed by Dunnett's post hoc test) and IC_50_ > 100 µM was eliminated from the assessments.

**Fig. 2:**
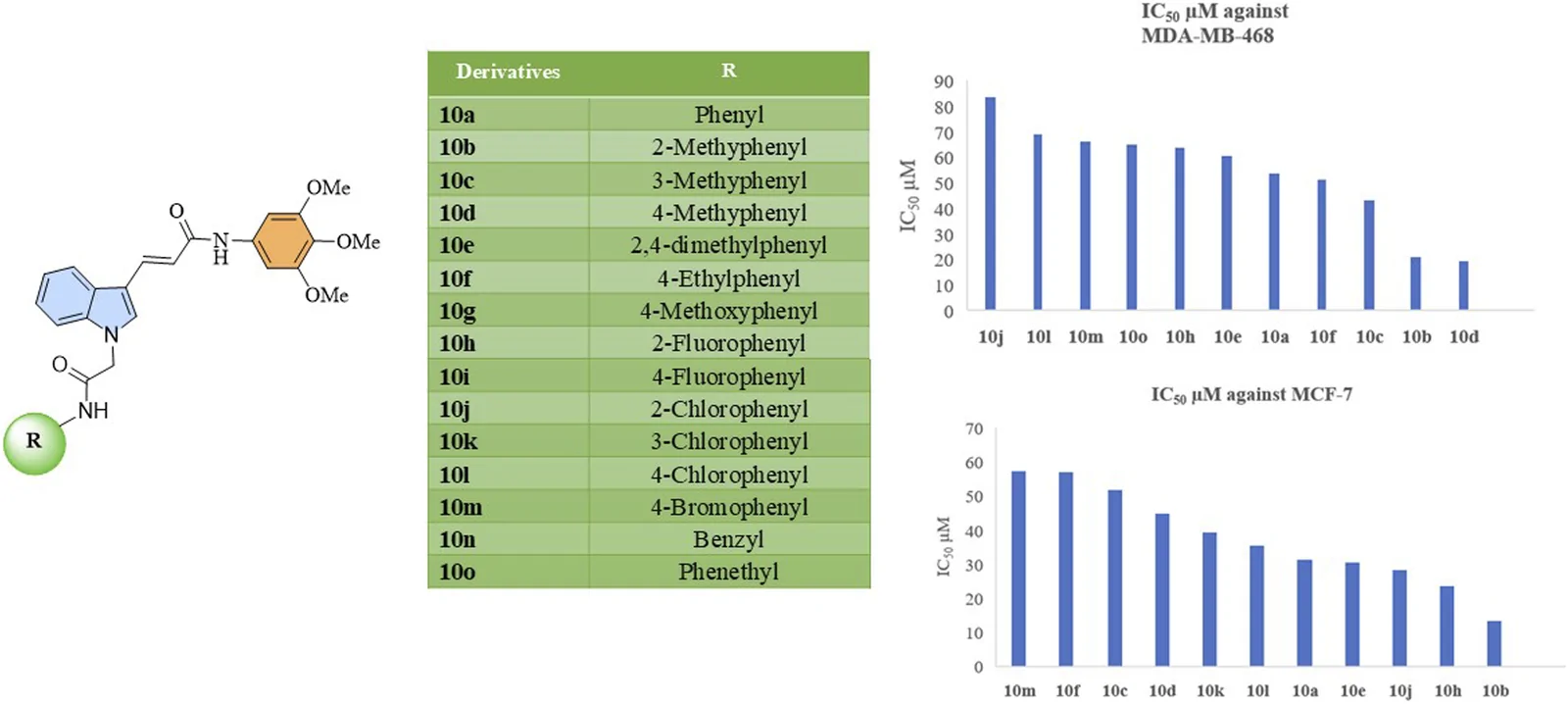
SAR summary.

Comparison of the alkyl-substituted analogues with the *para*-methoxy derivative (10g) indicates that electron-donating character alone is insufficient to account for biological activity. The methoxy group can participate in resonance through conjugation with the phenyl ring, whereas the methyl group exerts its electronic effect primarily through inductive and hyperconjugative interactions. In addition to its stronger electron-donating ability, the methoxy group introduces an additional hydrogen-bond acceptor and increases molecular polarity (TPSA = 106.26 Å^2^), suggesting that multiple structural factors alone influence cytotoxicity.

The SAR of the halogenated derivatives was more complex, indicating that the type and position of the halogen play important roles in cytotoxic activity. Fluorine, chlorine, and bromine differ in electronegativity, size, and hydrophobic character; nevertheless, no direct correlation between these parameters and biological activity was observed. For instance, the *ortho*-fluoro analog (10h) displayed moderate activity against both MCF-7 and Hep-G2 cells, whereas the *para*-fluoro derivative (10i) was completely inactive despite very similar cLogP and TPSA values. *Ortho* (10j) and *meta* (10k) analogs of chloro derivatives were also active against Hep-G2 cells, whereas the *para* isomer (10l) lost activity against this cell line and showed moderate activity against MCF-7 cells. Interestingly, the substitution of the chlorine atom with the larger and more polarizable bromine atom (10m) showed the best activity against Hep-G2 cells (IC_50_ = 21.53 µM).

Comparison of the benzyl (10n) and phenethyl (10o) derivatives further highlights the importance of conformational flexibility. Although these compounds differ by a single methylene unit, their biological profiles were markedly different. Compound (10n) was completely inactive against all tested cell lines, whereas (10o) displayed potent and selective activity against Hep-G2 cells (IC_50_ = 15.79 µM). Because both analogs possess similar cLogP (5.49 *vs.* 5.85) and TPSA (99.55 *vs.* 100.36 Å^2^) values, these differences are unlikely to arise solely from changes in global physicochemical properties. Instead, extension of the linker probably alters the orientation and conformation of the terminal aromatic ring, thereby modifying its interaction with the target.

Evaluation against the normal HEK-293 cell line further demonstrated that several active compounds retained measurable selectivity toward cancer cells. In particular, 10b, the most potent analog against MCF-7 breast cancer cell lines, exhibited an IC_50_ of 90.82 µM against HEK-293 cells, corresponding to approximately 6.7-fold selectivity relative to MCF-7 cells and 4.3-fold selectivity relative to MDA-MB-468 cells. Likewise, 10m, despite its potent activity against Hep-G2 cells, showed approximately 2-fold selectivity over normal cells. These findings indicate that the most active analogs possess an acceptable therapeutic window, although additional toxicological studies will be required to understand their safety profiles.

### 2.3 Time- and dose-dependent induction of apoptosis and necrosis

The mechanism of the cell death induced by the most active compound 10b was studied by annexin V-FITC/propidium iodide (PI). The analysis was performed at two times (24 h and 72 h) to evaluate time- and concentration-dependent effects on apoptotic and necrotic cell populations (Table 2, Fig S51 and S52).

**Table 2:** Flow cytometric analysis of apoptosis and necrosis induced by compound 10b in MCF-7 after 24 and 72 hours

Status (%)	Control, 24 h	10b-15 µM, 24 h	Control, 72 h	10b-15 µM, 72 h	10b-50 µM, 72 h
Live cells	79.3	36.2	80.1	25.5	21.02
Early apoptosis	14.1	32.9	5.81	4.01	0.2
Late apoptosis	1.15	26.0	1.97	12.4	3.0
Necrosis	5.51	4.94	11.4	58.0	75.7

Exposure to 10b (15 µM) for 24 h significantly decreased the number of viable cells compared to the untreated control. The live cell population decreased from 79.3% in control cells to 36.2% after treatment. This reduction was accompanied by a significant increase in both early and late apoptotic populations from 14.1% to 32.9% and from 1.15% to 26.0%, respectively. Conversely, the necrotic population was almost unaffected (5.51% in control *vs.* 4.94% after treatment), indicating that at this early time point, 10b predominantly induces apoptotic cell death rather than direct membrane-disruptive necrosis.

However, prolonged exposure (72 h) to 10b treatment at 15 µM resulted in a significant decrease in viable cells to 25.5%, with a marked increase in the necrotic population (58.0%) and a moderate increase in late apoptotic cells (12.4%). Increasing the concentration to 50 µM reduced the viable cell population to 21.2%. However, it markedly increased the necrotic cell population to 75.7%, while the early and late apoptotic populations decreased to 0.2% and 3.0%, respectively.

These results indicate dependence of the biological response to compound 10b on both the exposure time and the concentration. Initial 10b treatment induces a predominantly apoptotic process, whereas prolonged treatment, especially at higher concentrations, results in a predominantly necrotic process.

### 2.4 Cell cycle distribution in MCF-7 cells treated with 10b

Flow cytometric analysis of the cell cycle in MCF-7 breast cancer cells treated with the most potent analogue 10b, revealed a concentration-dependent effect at 7, 15, and 50 µM (Table 3 and Fig. 3). Treatment with 10b resulted in a progressive decrease in the G1 population to 55.1%, 46.4%, and 38.1% at 7, 15, and 50 µM, respectively, compared with the control group with 56.3%. In contrast, the S-phase population increased from 22.2% in the control to 38.1%, 37.1%, and 48.8%, indicating S-phase accumulation and suggesting inhibition of DNA replication progression.^22^ Meanwhile, the G2M population decreased markedly from 21.7% in the control to 10.9%, 10.7%, and 9.16% following treatment with compound 10b at 7, 15, and 50 µM, respectively. These findings demonstrate that compound 10b induces S-phase arrest.

**Table 3:** The distribution percentage of MCF-7 cells in different phases of the cell cycle in the presence or absence of 10b

Status	Control	10b-7 µM	10b-15 µM	10b-50 µM
G1	56.3	55.1	46.4	38.1
S	22.2	38.1	37.1	48.8
G2/M	21.7	10.9	10.7	9.16

**Fig. 3:**
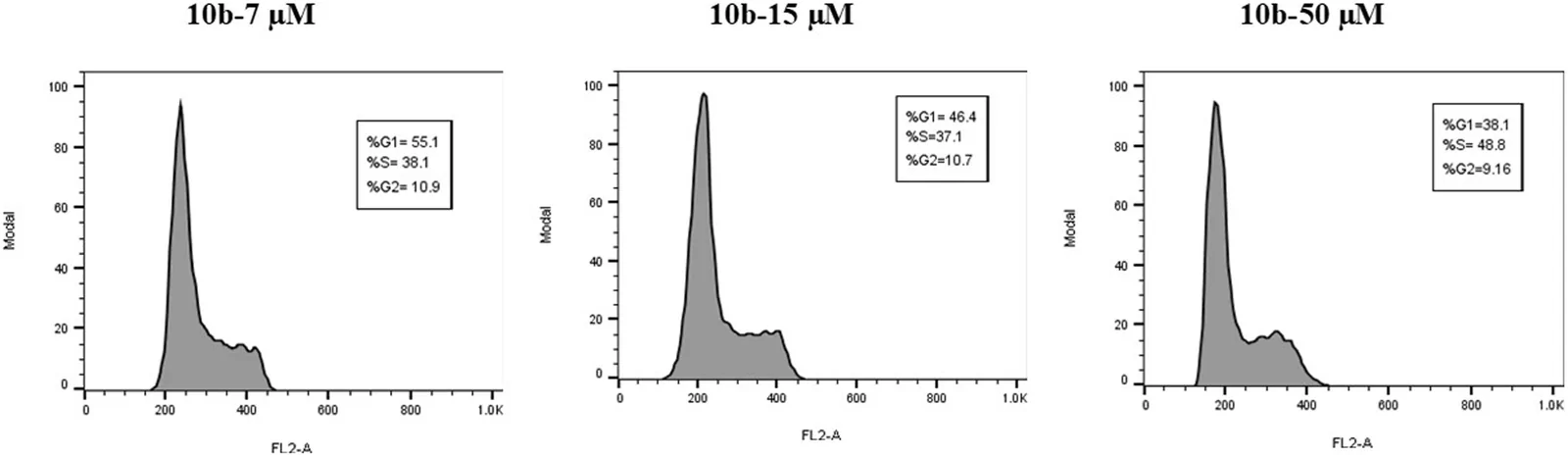
The effect of 10b on different phases of the MCF-7 cell cycle.

## 3. Conclusion

In the present study, a new series of indole-based trimethoxy aniline hybrids (10a–o) was designed through a fragment-based hybridization approach, synthesized, and evaluated as anticancer agents. All analogs were analyzed against breast (MCF-7, MDA-MB-468), liver (Hep-G2) cancer cell lines and the non-cancerous HEK-293 cell line to assess their anticancer potential and selectivity. Several compounds exhibited promising cytotoxic activity; among them, 10b, bearing an *ortho*-methyl substituent, emerged as the most promising lead, with moderate cytotoxicity against MCF-7 (IC_50_ = 13.52 ± 2.00 µM) and MDA-MB-468 (IC_50_ = 21.15 ± 2.21 µM), as well as moderate activity against Hep-G2 cells. Cell-cycle and apoptosis analyses revealed that compound 10b induced dose- and time-dependent cellular responses with S-phase arrest. Moreover, 10b primarily induced apoptosis after short-term exposure, while prolonged treatment at higher concentrations shifted the cell death pattern toward predominant necrosis.

Overall, this study identifies compound 10b as a promising indole-based lead for further anticancer drug development, particularly against breast cancer cell lines.

## 4. Methods and materials

^1^H and ^13^C-NMR spectra were recorded on a Bruker BioSpin GmbH spectrometer at 500 MHz, using TMS as an internal standard. Infrared spectra were recorded on a Bruker (USA) Equinox 55. Elemental analysis was performed on an Elementar Analysensystem GmbH VarioEL CHNS mode. HPLC analysis was performed on a Waters Alliance e2695 HPLC system with a Waters 2489 UV/Vis detector. HPLC analysis was performed to determine purity using a C18 column (4.6 × 150 mm, 5 µm) with acetonitrile: methanol (80 : 20, v/v), at a flow rate of 1.0 mL min^−1^, UV detection at 230 nm, and a total run time of 37 min.

### 4.1 Synthesis of compound 2

Initially, the Vilsmeier–Haack reaction (POCl_3_/DMF) was used to formylate the indole compound (1) at the C-3 position, which resulted in the synthesis of indole-3-carbaldehyde (2) in high yield and purity. In this method, the chloroiminium reagent is first produced *in situ*, which then undergoes an electrophilic reaction at the C-3 position of the indole ring – the most nucleophilic position in the molecule due to the conjugation of the ring nitrogen electron pair – and then, during the hydrolysis process, the desired aldehyde group is revealed at this position. This formylation method is simpler and more efficient than other alternative methods such as the Reimer–Tiemann reaction, which require more stringent reaction conditions and functional group protection and deprotection steps, and it also eliminates the need for protecting-group strategies.

### 4.2 Synthesis of compound (10a–o)

In the final step, the coupling reagent TBTU was used to form the amide bond between the acrylic acid group and 3,4,5-trimethoxyaniline. The use of this reagent instead of classical coupling reagents such as DCC and EDC is due to its mechanism of action, which creates an active OBt moiety with high reactivity, minimizes racemization, and minimizes the formation of by-products. In addition, the separation and purification of the product is also simpler. Given the reduced nucleophilicity of 3,4,5-trimethoxyaniline, a group of agents that generate methoxy electrons on aromatic rings, this reaction is expected to require a longer time than is usually recorded for similar reactions. However, the use of TBTU causes the reaction to be completed within the usual time frame for all synthesized derivatives, without incomplete conversion or unexpected increase in reaction time.
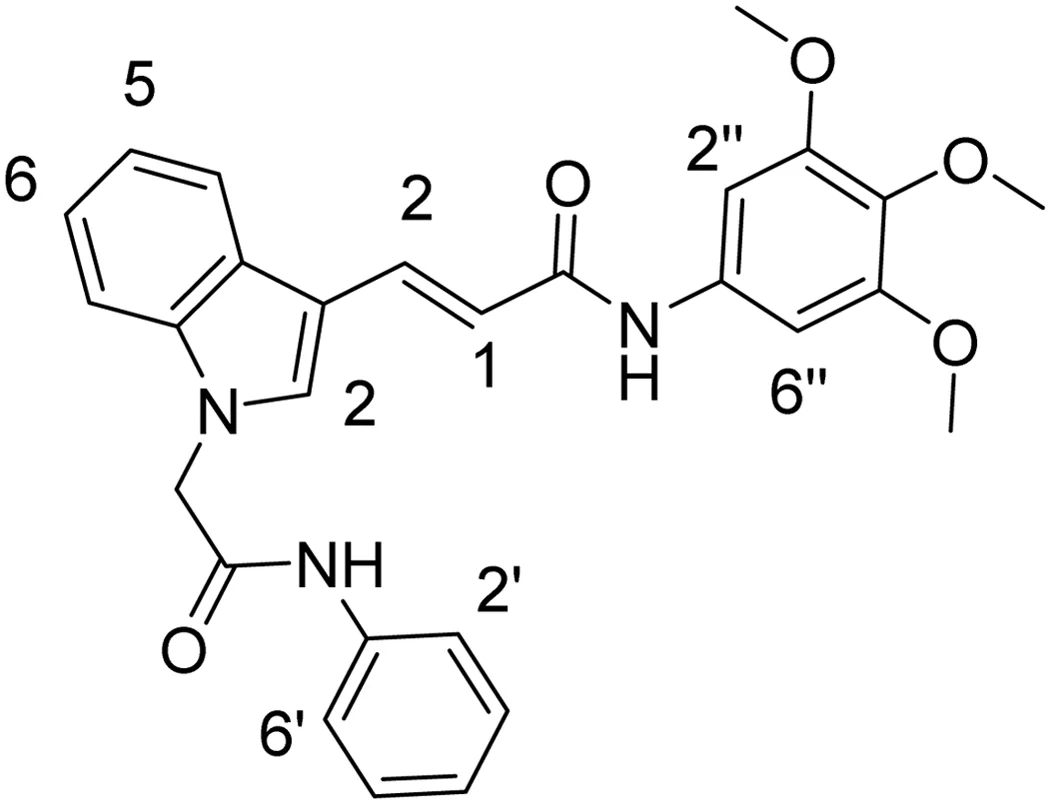


#### 4.2.1 3-(1-(2-oxo-2-(Phenylamino) ethyl)-1*H*-indol-3-yl)-*N*-(3,4,5-trimethoxyphenyl) acrylamide (10a)

Cream solid; yield: 78%; MP = 235–237; IR (KBr) = 3272 (N–H, amide), 3137 (=C–H, aromatic, vinyl), 2934 (C–H, aliphatic), 1669 (C=O, amide), 1606 (C=C, alkene, aromatic), 1506 (N–H bend, amide II), 1454, 1406 (C–N, amide III), 1312 (C–N), 1225, 1126 (C–O–C, Ar–OMe). ^1^H NMR (500 MHz, DMSO) *δ* 10.45 (s, 1H, NH_acetamide_), 9.98 (s, 1H, NH_trimethoxy_), 7.97 (d, *J* = 8.0 Hz, 1H, H_4_), 7.87 (s, 1H, H_2_), 7.72 (d, *J* = 15.8 Hz, 1H, H_2double bond_), 7.59 (d, *J* = 8.0 Hz, 2H, H2′, H6′), 7.52 (d, *J* = 8.0 Hz, 1H, H7), 7.31 (t, *J* = 8.0 Hz, 2H, H3′, H5′), 7.28–7.24 (m, 2H, H4′, H6), 7.09 (s, 2H, H2″, H6″), 7.06 (t, *J* = 8.0 Hz, 1H, H5), 6.81 (d, *J* = 15.8 Hz, 1H, H_1double bond_), 5.12 (s, 2H, CH_2_), 3.77 (s, 6H, CH_3_), 3.62 (s, 3H, CH_3_); ^13^C NMR (126 MHz, DMSO) *δ* 165.70 (C=O), 164.65 (C=O), 152.71, 138.58, 137.90, 135.85, 134.90, 133.77, 133.11, 128.80, 125.32, 123.53, 122.56, 120.80, 120.03, 119.10, 116.57, 111.66, 110.74, 96.56 (C2″, C6″), 60.06 (CH_3_), 55.61 (CH_3_), 49.13 (C_CH_2__); anal. calcd: C_28_H_27_N_3_O_5_; C, 69.26; H, 5.61; N, 8.65; found: C, 69.33; H, 5.76; N, 8.69. HPLC purity of 97.44%.
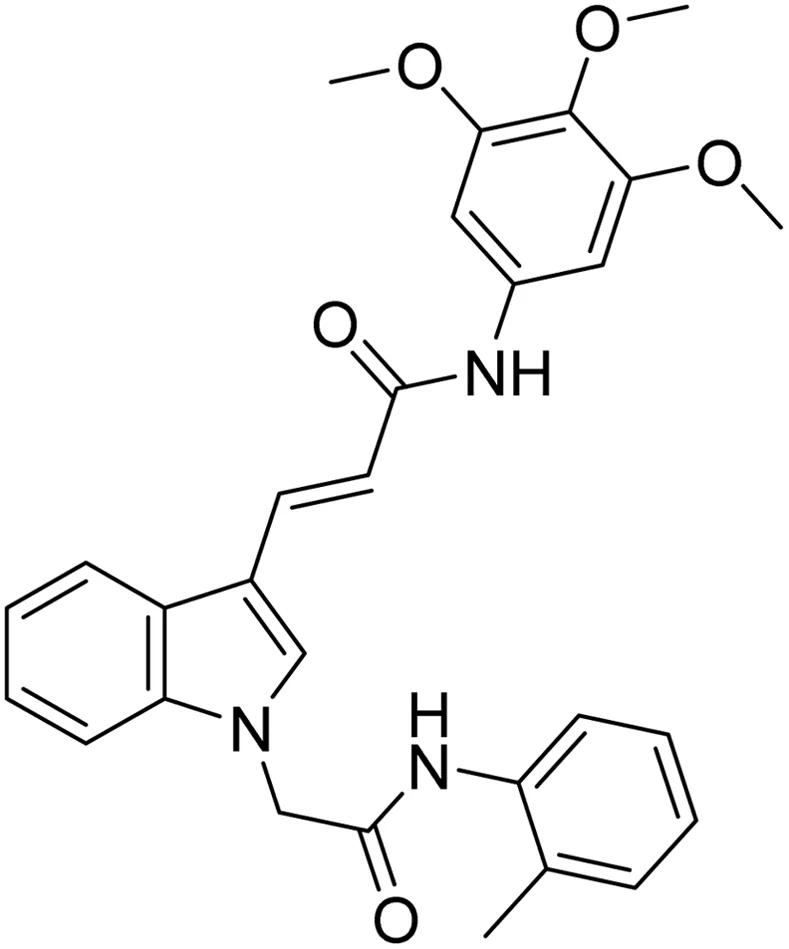


#### 4.2.2 3-(1-(2-Oxo-2-(*o*-tolylamino)ethyl)-1*H*-indol-3-yl)-*N*-(3,4,5-trimethoxyphenyl)acrylamide (10b)

Cream solid; yield: 78%; MP = 236–238; IR = 3264 (N–H, amide), 2933 (C–H, aromatic), 1668 (C=O, amide), 1609 (C=C, alkene, aromatic), 1510 (N–H, amide II), 1459 (CH_3_, *o*-methyl), 1393 (C–N, amide III), 1290 (C–N), 1177, 1128 (C–O–C, Ar–OMe) cm^−1^. ^1^H NMR (500 MHz, DMSO) *δ* 10.00 (s, 1H, NH_acetamide_), 9.76 (s, 1H, NH_trimethoxy_), 7.99 (d, *J* = 8.0 Hz, 1H, H4), 7.89 (s, 1H, H2), 7.75 (d, *J* = 15.8 Hz, 1H, H_2double bond_), 7.57 (d, *J* = 8.0 Hz, 1H, H7), 7.42 (d, *J* = 8.0 Hz, 1H, H6′), 7.31 (t, *J* = 8.0 Hz, 1H, H5′), 7.27 (t, *J* = 8.0 Hz, 1H, H4′), 7.22 (d, *J* = 8.0 Hz, 1H, H3′), 7.15 (t, *J* = 8.0 Hz, 1H, H6), 7.11 (s, 2H, H2″, H6″), 7.08 (t, *J* = 8.0 Hz, 1H, H5), 6.83 (d, *J* = 15.8 Hz, 1H, H1 H_1double bond_), 5.18 (s, 2H, CH_2_), 3.78 (s, 6H, CH_3_), 3.63 (s, 3H, 3H, CH_3_), 2.23 (s, 3H, CH_3_); ^13^C NMR (126 MHz, DMSO) *δ* 165.82 (C=O), 164.68 (C=O), 152.73, 137.83, 135.86, 135.68, 134.88, 133.78, 133.17, 131.55, 130.34, 125.96, 125.40, 124.70, 122.56, 120.84, 120.08, 116.60, 111.69, 110.70, 96.62 (C2″, C6″), 60.06, (CH_3_),55.63 (CH_3_), 48.96 (CH_2_), 17.78 (C CH_3_); anal. calcd: C_29_H_27_N_3_O_5_; C, 69.72; H, 5.85; N, 8.41; found: C, 69.80; H, 5.91; N, 8.55.HPLC purity of 97.94%.
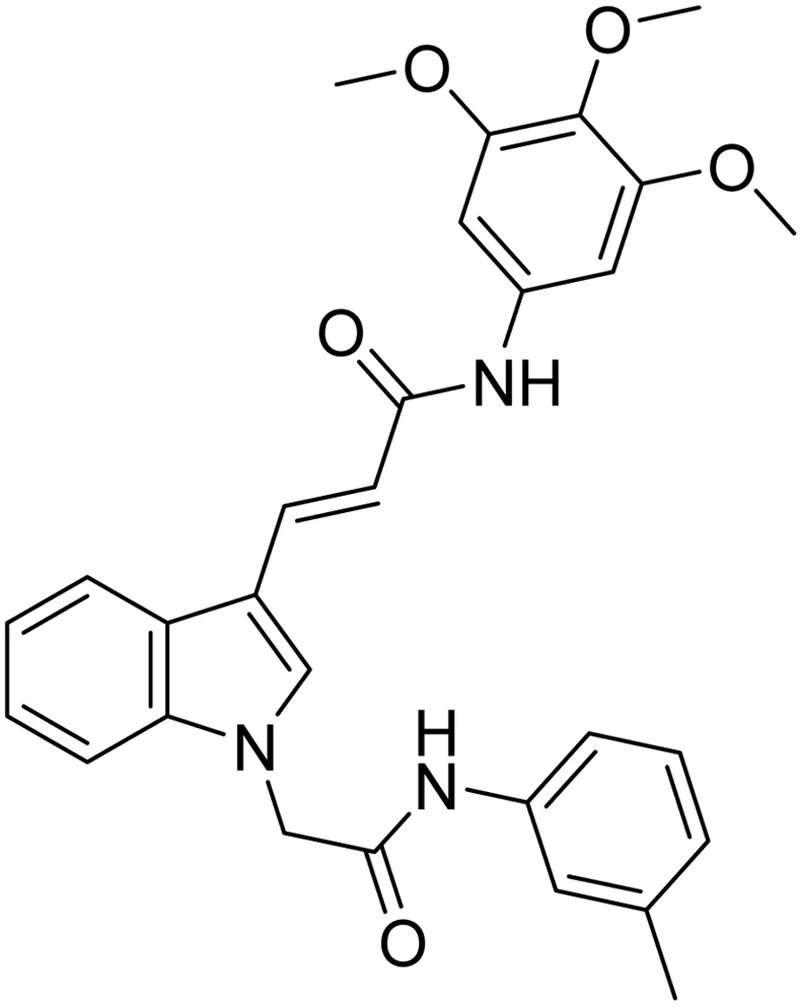


#### 4.2.3 3-(1-(2-Oxo-2-(*m*-tolylamino) ethyl)-1*H*-indol-3-yl)-*N*-(3,4,5-trimethoxyphenyl) acrylamide (10c)

Cream solid; yield: 74%; MP = 237–239; IR (ATR) = 3412 (N–H, amide), 2935 (C–H, aliphatic), 1669 (C=O, amide), 1608 (C=C, alkene, aromatic), 1507 (N–H bend, amide II), 1459, 1409 (CH_3_ bend), 1297 (C–N, amide III), 1221, 1128 (C–O–C, Ar–OMe) cm^−1^. ^1^H NMR (500 MHz, DMSO) *δ* 10.40 (s, 1H, NH_acetamide_), 10.00 (s, 1H, NH_trimethoxy_), 7.99 (d, *J* = 8.0 Hz, 1H, H4), 7.87 (s, 1H, H2), 7.75 (d, *J* = 15.8 Hz, 1H, H_2double bond_), 7.53 (d, *J* = 8.0 Hz, 1H, 7H), 7.44 (s, 1H, H2), 7.39 (d, *J* = 8.0 Hz, 1H, H6′), 7.30–7.24 (m, 2H, H6, H5′), 7.19 (t, *J* = 8.0 Hz, 1H, H5), 7.12 (s, 2H, H2″, H6″), 6.88 (d, *J* = 8.0 Hz, 1H, H4′), 6.84 (d, *J* = 15.8 Hz, 1H, H_1double bond_), 5.12 (s, 2H, CH_2_), 3.78 (s, 6H, CH_3_), 3.64 (s, 3H, CH_3_), 2.26 (s, 3H, CH_3_): ^13^C NMR (126 MHz, DMSO) *δ* 165.63 (C=O), 164.69 (C=O), 152.73, 138.51, 138.00, 137.91, 135.87, 134.88, 133.79, 133.17, 128.62, 125.36, 124.24, 122.56, 120.81, 120.04, 119.69, 116.61, 116.33, 111.69, 110.73, 96.63 (C2″, C6″), 60.06 (CH_3_), 55.62 (CH_3_), 49.19 (CH_2_), 21.08 (CH_3_); anal. calcd: C_29_H_27_N_3_O_5_; C, 69.72; H, 5.85; N, 8.41; found: C, 69.83; H, 5.98; N, 8.57.
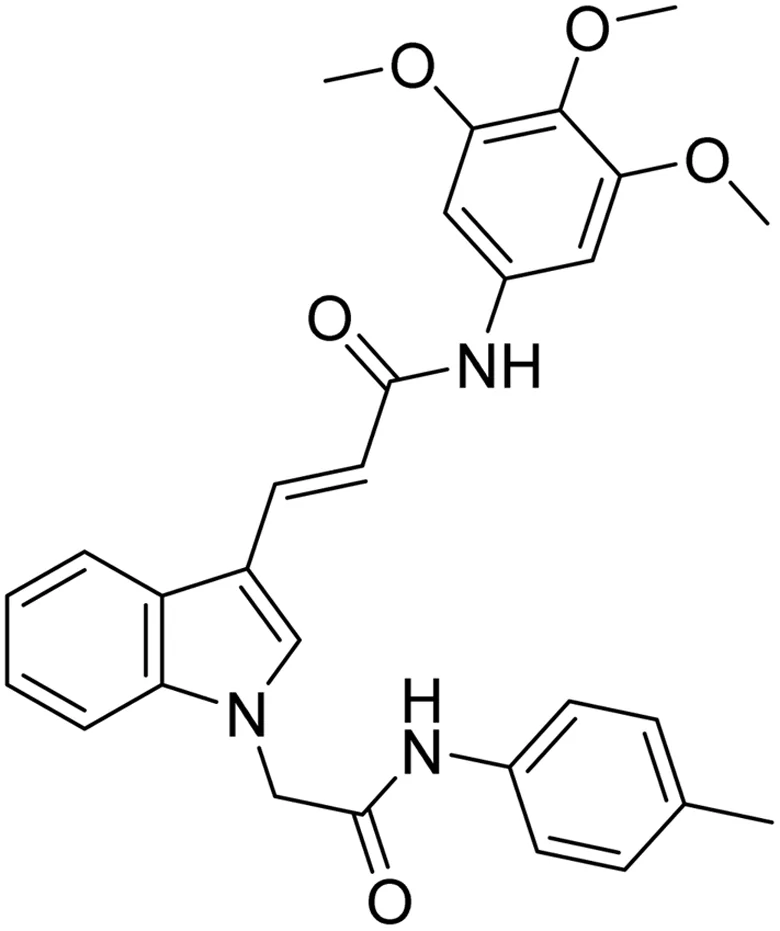


#### 4.2.4 3-(1-(2-Oxo-2-(*p*-tolylamino) ethyl)-1*H*-indol-3-yl)-*N*-(3,4,5-trimethoxyphenyl) acrylamide (10d)

Cream solid; yield: 76%; MP = 205–207; IR (ATR) = 3263 (N–H, amide), 3045 (=C–H, aromatic/vinyl), 2932 (C–H, aliphatic), 1661 (C=O, amide), 1607 (C=C, alkene, aromatic), 1533 (N–H bend, amide II), 1461, 1410 (CH_3_ bend), 1352 (C–N, amide III), 1309, 1233, 1181, 1132 (C–O–C, Ar–OMe) cm^−1^. ^1^H NMR (500 MHz, DMSO) *δ* 10.40 (s, 1H, NH_acetamide_), 10.01 (s, 1H, NH_trimethoxy_), 8.00 (d, *J* = 8.0 Hz, 1H, H4), 7.88 (s, 1H, H2), 7.77 (d, *J* = 15.8 Hz, 1H, H_2double bond_), 7.54 (d, *J* = 8.0 Hz, 1H, H7), 7.50 (d, *J* = 8.0 Hz, 2H, H2′, H6′), 7.31–7.23 (m, 2H, H5, H6), 7.13 (s, 2H, H2″, H6″), 7.12 (d, *J* = 8.0 Hz, 2H, H3′, H5′), 6.85 (d, *J* = 15.8 Hz, 1H, H_1double bond_), 5.12 (s, 2H, CH_2_), 3.78 (s, 6H, CH_3_), 3.64 (s, 3H, CH_3_), 2.24 (s, 3H, CH_3_); ^13^C NMR (126 MHz, DMSO) *δ* 165.45 (C=O), 164.71 (C=O), 152.74, 137.92, 136.08, 135.89, 134.89, 133.81, 133.17, 132.51, 129.16, 125.36, 122.56, 120.81, 120.05, 119.16, 116.61, 111.69, 110.75, 96.63 (C2″, C6″_trimethoxy_), 60.06 (CH_3_), 55.62 (CH_3_), 49.14 (CH_2_), 20.36 (CH_3_); anal. calcd: C_29_H_27_N_3_O_5_; C, 69.72; H, 5.85; N, 8.41; found: C, 69.80; H, 5.92; N, 8.53.
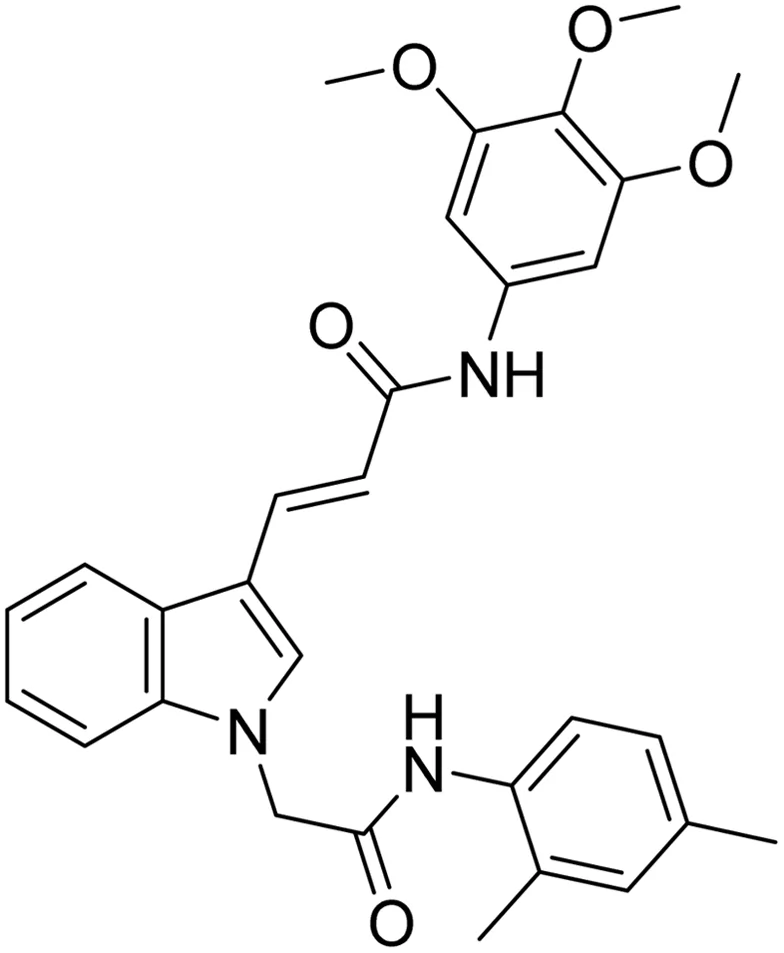


#### 4.2.5 3-(1-(2-((2,4-Dimethylphenyl) amino)-2-oxoethyl)-1*H*-indol-3-yl)-*N*-(3,4,5-trimethoxyphenyl) acrylamide (10e)

Cream solid; yield: 81%; MP = 240–242; IR (ATR): *ν* = 3264 (N–H, amide), 2933, 2835 (C–H, aliphatic/OMe), 1659 (C=O, amide), 1609 (C=C, alkene/aromatic), 1534 (N–H bend, amide II), 1460, 1412 (CH_3_ bend), 1353 (C–N, amide III), 1278, 1230, 1182, 1131 (C–O–C, Ar–OMe) cm^−1^.^1^H NMR (500 MHz, DMSO) *δ* 9.98 (s, 1H, NH_acetamide_), 9.68 (s, 1H, NH_trimethoxy_), 7.98 (d, *J* = 8.0 Hz, 1H, H4), 7.88 (s, 1H, H2), 7.74 (d, *J* = 15.8 Hz, 1H, H_2double bond_), 7.56 (d, *J* = 8.0 Hz, 1H, H7), 7.30 (t, *J* = 7.6 Hz, 2H6, H5, H), 7.26 (d, *J* = 8.0 Hz, 1H, H6′), 7.11 (s, 2H, H2″, H6″), 7.02 (s, 1H, H3′), 6.95 (d, *J* = 8.0 Hz, 1H, H5′), 6.83 (d, *J* = 15.8 Hz, 1H, H_1double bond_), 5.14 (s, 2H, CH_2_), 3.77 (s, 6H, CH_3_), 3.63 (s, 3H, CH_3_), 2.22 (s, 3H, CH_3_), 2.18 (s, 3H, CH_3_); ^13^C NMR (126 MHz, DMSO) *δ* 165.74 (C=O), 164.68 (C=O), 152.74, 137.83, 135.86, 134.85, 134.53, 133.78, 133.20, 133.08, 131.54, 130.84, 126.46, 125.42, 124.79, 122.54, 120.83, 120.08, 116.60, 111.67, 110.70, 96.66 (C2″, C6″), 60.07 (CH_3_), 55.64 (CH_3_), 48.95 (CH_2_), 20.37 (CH_3_), 17.69 (CH_3_); anal. calcd: C_30_H_31_N_3_O_5_; C, 70.16; H, 6.08; N, 8.18; found: C, 70.24; H, 6.21; N, 8.23.
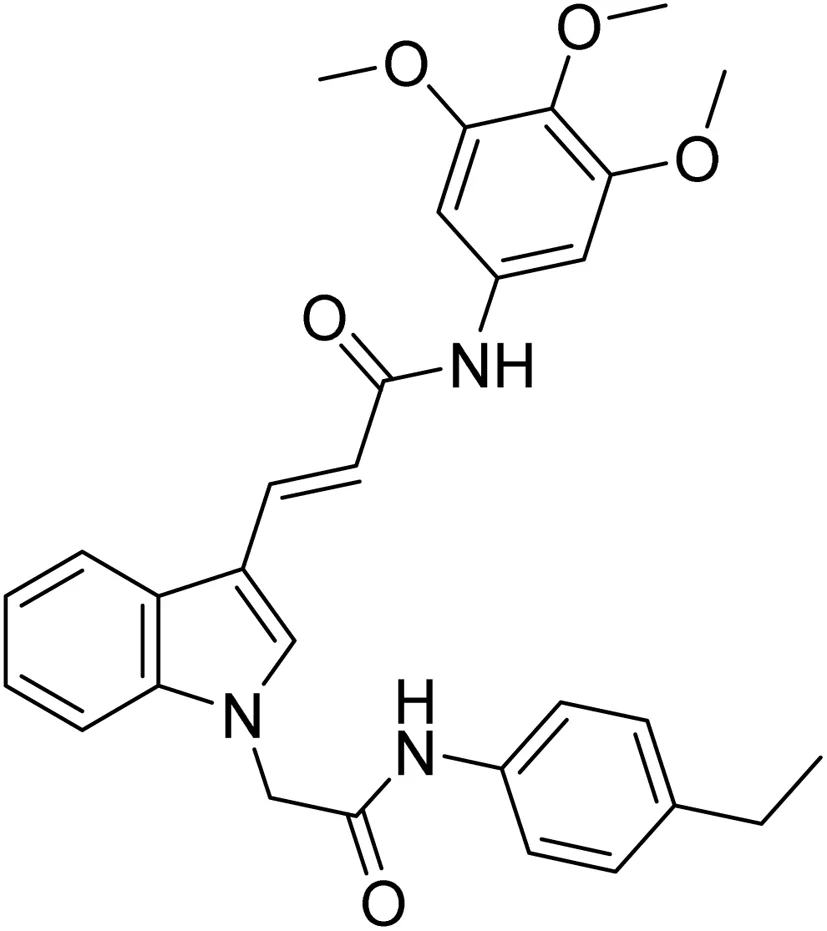


#### 4.2.6 3-(1-(2-((4-Ethylphenyl) amino)-2-oxoethyl)-1*H*-indol-3-yl)-*N*-(3,4,5-trimethoxyphenyl) acrylamide (10f)

Cream solid; yield: 84%; MP = 244–246; IR = 3287 (N–H, amide), 2963 (C–H, aliphatic), 1663 (C=O, amide), 1606 (C=C, alkene, aromatic), 1518 (N–H, amide), 1461 (CH_2_, CH_3_, ethyl), 1411 (C–N, amide), 1309 (C–N), 1232, 1129 (C–O–C, Ar–Ome) cm^−1^. ^1^H NMR (500 MHz, DMSO) *δ* 10.40 (s, 1H, NH_acetamide_), 10.00 (s, 1H, NH_trimethoxy_), 7.99 (d, *J* = 8.0 Hz, 1H, H4), 7.87 (s, 1H, H2), 7.75 (d, *J* = 15.8 Hz, 1H, H_2double bond_), 7.55–7.52 (m, 1H, H7), 7.51 (d, *J* = 9.0 Hz, 2H, H2′, H6′), 7.27 (t, *J* = 8.0 Hz, 2H, H5, H6), 7.14 (d, *J* = 9.0 Hz, 2H, H3′, H5′), 7.12 (s, 2H, H2″, H6″), 6.84 (d, *J* = 15.8 Hz, 1H, H_1double bond_), 5.12 (s, 2H, CH_2_), 3.78 (s, 6H, CH_3_), 3.63 (s, 3H, CH_3_), 2.53 (q, *J* = 7.5 Hz, 2H, CH_2_), 1.13 (t, *J* = 7.5 Hz, 3H, CH_3_); ^13^C NMR (126 MHz, DMSO) *δ* 165.44 (C=O), 164.69 (C=O), 152.73, 138.97, 137.89, 136.27, 135.87, 134.89, 133.78, 133.15, 127.96, 125.36, 122.55, 120.80, 120.04, 119.22, 116.59, 111.67, 110.73, 96.61 (C2″, C6″), 60.06 (CH_3_), 55.62 (CH_3_), 49.15 (CH_2_), 27.52 (CH_2_), 15.57 (CH_3_); anal. calcd: C_30_H_31_N_3_O_5_; C, 70.16; H, 6.08; N, 8.18; found: C, 70.27; H, 6.11; N, 8.29.
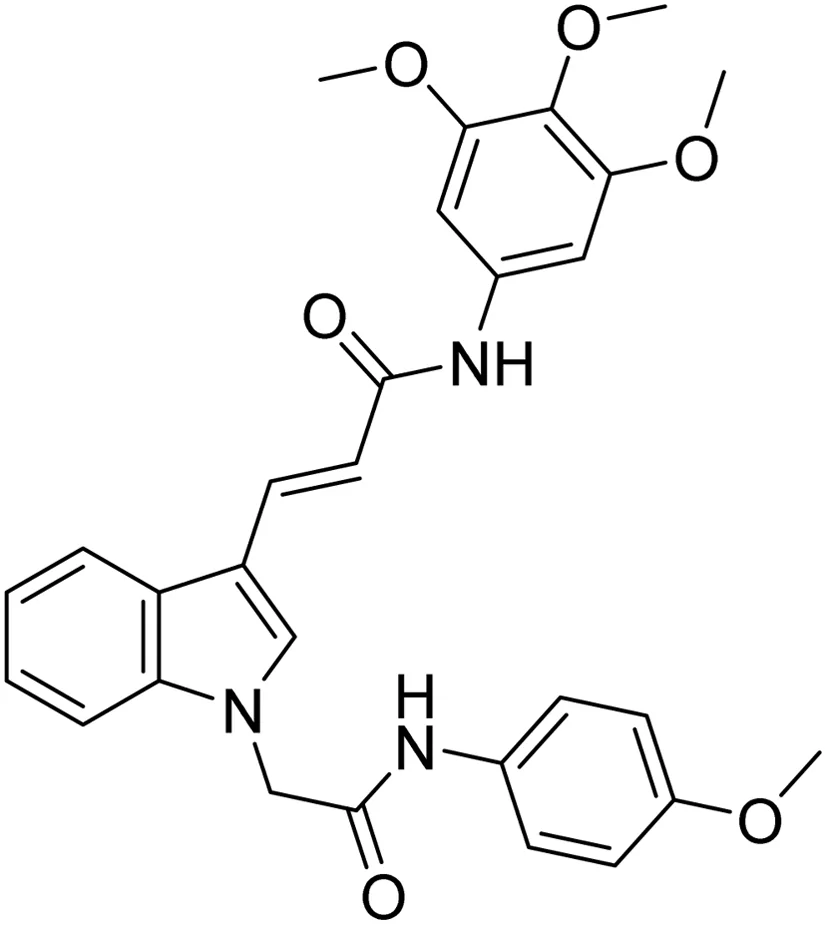


#### 4.2.7 3-(1-(2-((4-Methoxyphenyl) amino)-2-oxoethyl)-1*H*-indol-3-yl)-*N*-(3,4,5-trimethoxyphenyl) acrylamide (10g)

Cream solid; yield: 84%; MP = 238–240; IR (ATR) = 3268 (N–H, amide), 3053 (C–H, aromatic), 2933, 2834 (C–H, aliphatic), 1663 (C=O, amide), 1608 (C=C, alkene/aromatic), 1513 (N–H bend, amide II), 1459, 1407 (CH_2_ bend), 1352 (C–N, amide III), 1298, 1238, 1128 (C–O–C, Ar–Ome) cm^−1^.^1^H NMR (500 MHz, DMSO) *δ* 10.30 (s, 1H, NH_acetamide_), 9.98 (s, 1H, NH_trimethoxy_), 7.98 (d, *J* = 8.0 Hz, 1H, H4), 7.86 (s, 1H, H2), 7.75 (d, *J* = 15.8 Hz, 1H, H_2double bond_), 7.55–7.53 (m, 1H. H7), 7.51 (d, *J* = 9.0 Hz, 2H, H2′, H6′), 7.26 (t, *J* = 8.0 Hz, 2H, H5, H6), 7.11 (s, 2H, H2″, H6″), 6.88 (d, *J* = 9.0 Hz, 2H, H3′, H5′), 6.83 (d, *J* = 15.8 Hz, 1H, H_1double bond_), 5.09 (s, 2H, CH_2_), 3.77 (s, 6H, CH_3_), 3.70 (s, 3H, CH_3_), 3.63 (s, 3H, CH_3_).^13^C NMR (126 MHz, DMSO) *δ* 165.15 (C=O), 164.69 (C=O), 155.39, 152.72, 137.89, 135.86, 134.88, 133.80, 133.16, 131.69, 125.35, 122.55, 120.80, 120.68, 120.04, 116.58, 113.91, 111.66, 110.73, 96.61 (C2″, C6″), 60.06 (CH_3_), 55.62 (CH_3_), 55.09 (CH_3_), 49.10 (CH_2_); anal. calcd: C_29_H_29_N_3_O_6_; C, 67.56; H, 5.67; N, 8.15; found: C, 67.63; H, 5.71; N, 8.27.
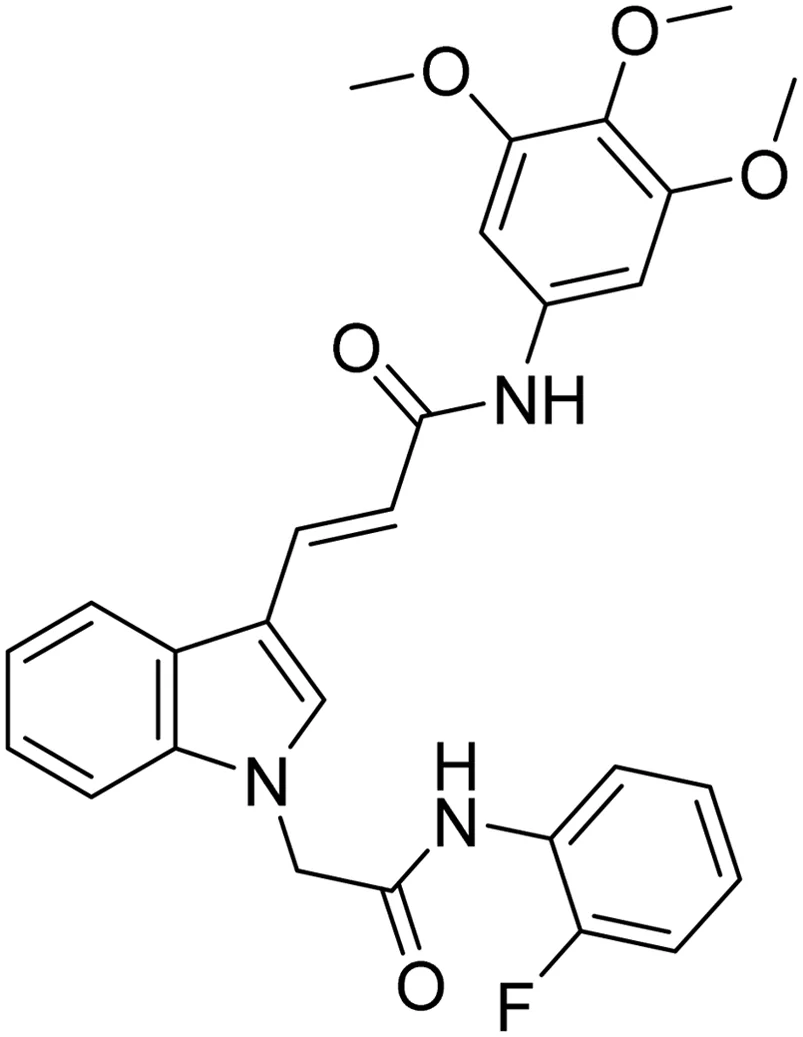


#### 4.2.8 3-(1-(2-((2-Fluorophenyl) amino)-2-oxoethyl)-1*H*-indol-3-yl)-*N*-(3,4,5-trimethoxyphenyl) acrylamide (10h)

Cream solid; yield: 76%; MP = 246–248; IR (ATR) = 3265 (N–H, amide), 3046 (=C–H, aromatic/vinyl), 2934, 2834 (C–H, aliphatic/OMe), 1675 (C=O, amide), 1609 (C=C, alkene/aromatic), 1536 (N–H bend, amide II), 1459, 1411 (CH_2_ bend), 1353 (C–N, amide III), 1307, 1225, 1133 (C–O–C, Ar–OMe) cm^−1^.^1^H NMR (500 MHz, DMSO) *δ* 10.30 (s, 1H, NH_acetamide_), 10.05 (s, 1H, NH_trimethoxy_), 8.02 (d, *J* = 8.0 Hz, 1H, H4), 7.96–7.89 (m, 2H, H2, H6′), 7.81 (d, *J* = 15.8 Hz, 1H, H_2double bond_), 7.56 (d, *J* = 8.0 Hz, 1H, H7), 7.32–7.25 (m, 4H, H5, H6, H3′, H5′), 7.16 (s, 2H, H2″, H6″), 7.14–7.12 (m, 1H, H4′), 6.89 (d, *J* = 15.8 Hz, 1H, H_1double bond_), 5.25 (s, 2H, CH_2_), 3.80 (s, 6H, CH_3_), 3.66 (s, 3H, CH_3_);^13^C NMR (126 MHz, DMSO) *δ* 166.35 (C=O), 164.75 (C=O), 154.37, 152.76, 152.42, 137.90, 135.90, 134.87, 133.83, 133.18, 125.73, 125.64, 125.39, 124.38, 123.63, 122.62, 120.87, 120.09, 116.69, 115.55, 115.40, 111.80, 110.72, 96.61 (C2″, C6″), 60.06 (CH_3_), 55.59 (CH_3_), 48.95 (CH_2_); anal. calcd: C_28_H_29_FN_3_O_5_; C, 66.79; H, 5.20; N, 8.35; found: C, 66.87; H, 5.36; N, 8.41. HPLC purity of 97.45%.
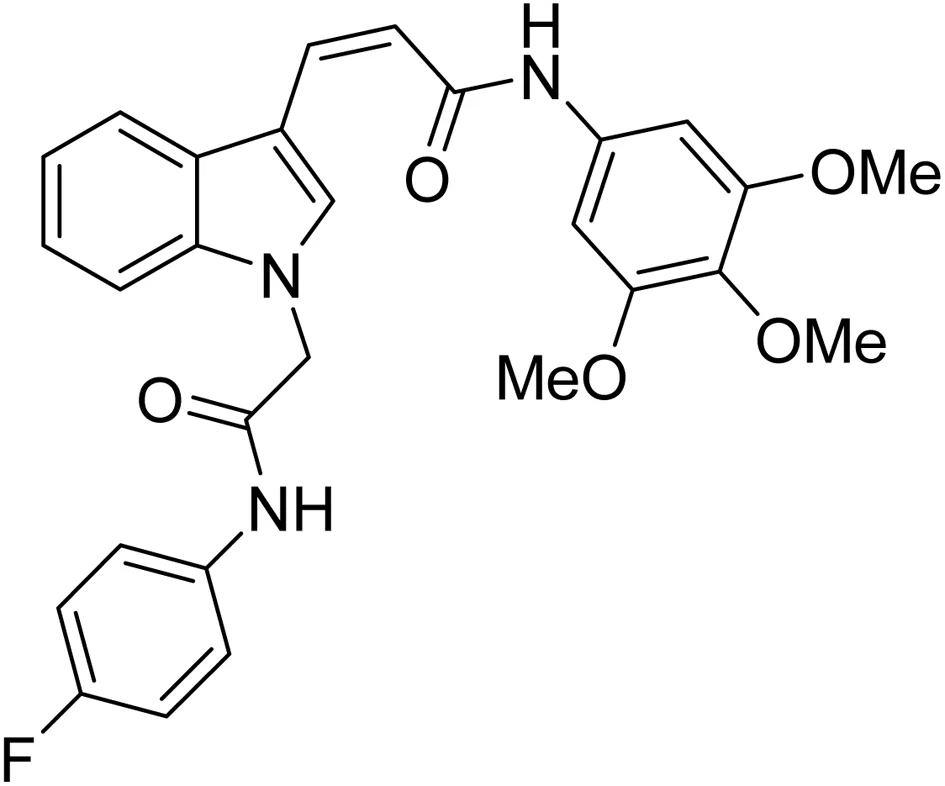


#### 4.2.9 3-(1-(2-((4-Fluorophenyl) amino)-2-oxoethyl)-1*H*-indol-3-yl)-*N*-(3,4,5-trimethoxyphenyl) acrylamide (10i)

Cream solid; yield: 78%; MP = 241–243; IR (ATR) = 3258 (N–H, amide), 3120, 3044 (=C–H, aromatic/vinyl), 2931, 2834 (C–H, aliphatic/OMe), 1661 (C=O, amide), 1606 (C=C, alkene/aromatic), 1539 (N–H bend, amide II), 1459, 1408 (CH_2_ bend), 1352 (C–N, amide III), 1307, 1236, 1131 (C–O–C, Ar–OMe) cm^−1^. ^1^H NMR (500 MHz, DMSO) *δ* 10.51 (s, 1H, NH_acetamide_), 10.00 (s, 1H, NH_trimethoxy_), 8.00 (d, *J* = 8.0 Hz, 1H, H4), 7.88 (s, 1H, H2), 7.76 (d, *J* = 15.8 Hz, 1H, H_2double bond_), 7.63 (d, *J* = 8.8 Hz, 2H, H2′, H6′), 7.54 (d, *J* = 8.0 Hz, 1H, H7), 7.32–7.23 (m, 2H, H5, H6), 7.17 (d, *J* = 8.8 Hz, 2H, H3′, H5′), 7.13 (s, 2H, H2″, H6″), 6.85 (d, *J* = 15.8 Hz, 1H, H_1double bond_), 5.13 (s, 2H, CH_2_), 3.78 (s, 6H, CH_3_), 3.64 (s, 3H, CH_3_); ^13^C NMR (126 MHz, DMSO) *δ* 165.66 (C=O), 164.70 (C=O), 159.06, 157.15, 152.73, 137.92, 135.87, 134.98, 134.88, 133.80, 133.14, 125.35, 122.58, 120.95, 120.88, 120.83, 120.05, 116.63, 115.47, 115.29, 111.73, 110.75, 96.58 (C2″, C6″), 60.05 (CH_3_), 55.59 (CH_3_), 49.07 (CH_2_); anal. calcd: C_28_H_29_FN_3_O_5_; C, 66.79; H, 5.20; N, 8.35; found: C, 66.94; H, 5.31; N, 8.47.
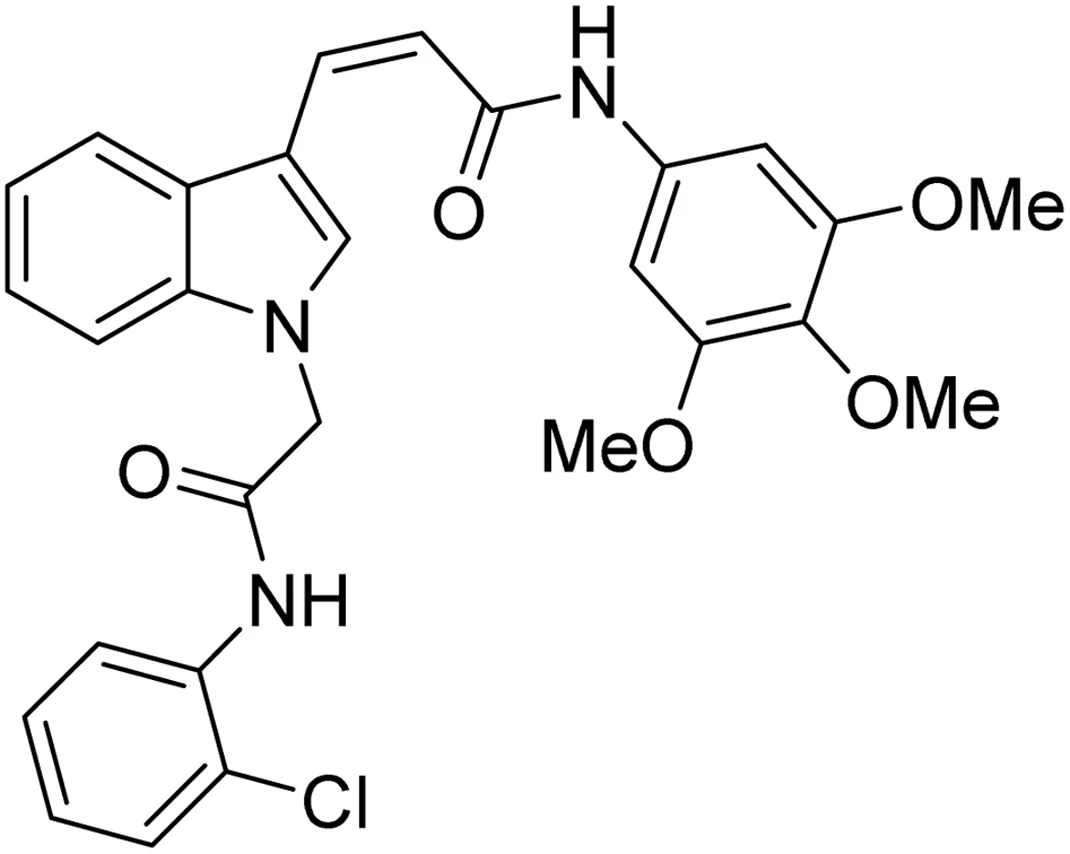


#### 4.2.10 3-(1-(2-((2-Chlorophenyl) amino)-2-oxoethyl)-1*H*-indol-3-yl)-*N*-(3,4,5-trimethoxyphenyl) acrylamide (10j)

Cream solid; yield: 72%; MP = 235–237; IR (ATR) = 3262 (N–H, amide), 3050 (=C–H, aromatic), 2934 (C–H, aliphatic), 1673 (C=O, amide), 1609 (C=C, alkene,aromatic), 1535 (N–H bend, amide II), 1460, 1413 (CH_2_ bend), 1352 (C–N, amide III), 1289, 1231, 1185, 1132 (C–O–C, Ar–OMe), 1058 (C–Cl) cm^−1^. ^1^H NMR (500 MHz, DMSO) *δ* 10.03 (s, 1H, NH_acetamide_), 9.99 (s, 1H, NH_trimethoxy_), 8.02 (d, *J* = 8.0 Hz, 1H, H4), 7.91 (s, 1H, H2), 7.81–7.75 (m, 2H, H6′, H_2double bond_), 7.58 (d, *J* = 8.0 Hz, 1H, H7), 7.50 (d, *J* = 8.0 Hz, 1H, H5′), 7.31–7.27 (m, 3H, H3′, H5, H6), 7.18 (t, *J* = 8.0 Hz, 1H, H4′), 7.14 (s, 2H, H2″, H6″), 6.87 (d, *J* = 15.8 Hz, 1H, H_1double bond_), 5.26 (s, 2H, CH_2_), 3.79 (s, 6H, CH_3_), 3.65 (s, 3H, CH_3_); ^13^C NMR (126 MHz, DMSO) *δ* 166.37 (C=O), 164.71 (C=O), 152.75, 137.84, 135.88, 134.79, 134.32, 133.77, 133.19, 129.51, 127.45, 126.48, 126.18, 125.68, 125.42, 122.64, 120.91, 120.10, 116.75, 111.84, 110.73, 96.64 (C2″, C6″), 60.07 (CH_3_), 55.61 (CH_3_), 48.96 (CH_2_); anal. calcd: C_28_H_29_ClN_3_O_5_; C, 64.68; H, 5.04; N, 8.08; found: C, 64.92; H, 5.19; N, 8.21.
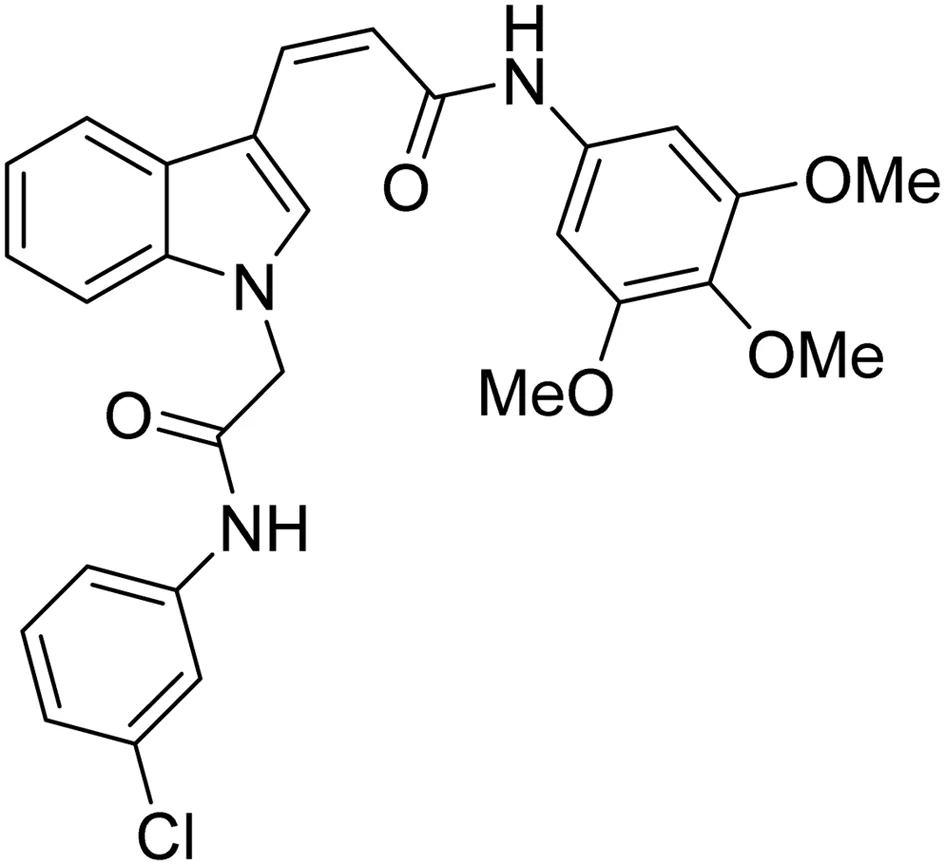


#### 4.2.11 3-(1-(2-((3-Chlorophenyl) amino)-2-oxoethyl)-1*H*-indol-3-yl)-*N*-(3,4,5-trimethoxyphenyl) acrylamide (10k)

Cream solid; yield: 73%; MP = 233–235; IR (ATR) = 3265 (N–H, amide), 3067 (=C–H, aromatic), 2937 (C–H, aliphatic), 1670 (C=O, amide), 1605 (C=C, alkene, aromatic), 1535 (N–H bend, amide II), 1463, 1413 (CH_2_ bend), 1351 (C–N, amide III), 1301, 1232, 1180, 1125 (C–O–C, Ar–OMe) cm^−1^. ^1^H NMR (500 MHz, DMSO) *δ* 10.61 (s, 1H, NH_acetamide_), 9.95 (s, 1H, NH_trimethoxy_), 7.97 (d, *J* = 8.0 Hz, 1H, H4), 7.86 (s, 1H, H2), 7.78 (s, 1H, H2′), 7.72 (d, *J* = 15.8 Hz, 1H, H_2double bond_), 7.52 (d, *J* = 8.0 Hz, 1H, H7), 7.46 (d, *J* = 8.0 Hz, 1H, H6′), 7.35 (t, *J* = 8.0 Hz, 1H, H5′), 7.26 (t, *J* = 8.0 Hz, 2H, H5, H6), 7.13 (d, *J* = 8.0 Hz, 1H, H4′), 7.09 (s, 2H, H2″, H6″), 6.81 (d, *J* = 15.7 Hz, 1H, H_1double bond_), 5.14 (s, 2H, CH_2_), 3.77 (s, 6H, CH_3_), 3.63 (s, 3H, CH_3_); ^13^C NMR (126 MHz, DMSO) *δ* 166.25 (C=O), 164.69 (C=O), 152.76, 140.05, 137.98, 135.90, 134.92, 133.80, 133.15, 130.61, 125.36, 123.32, 122.65, 120.88, 120.08, 118.67, 117.58, 111.79, 110.82, 96.60 (C2″, C6″), 60.12 (CH_3_), 55.65 (CH_3_), 49.15^.^(CH_2_); anal. calcd: C_28_H_29_ClN_3_O_5_; C, 64.68; H, 5.04; N, 8.08; found: C, 64.90; H, 5.17; N, 8.24.
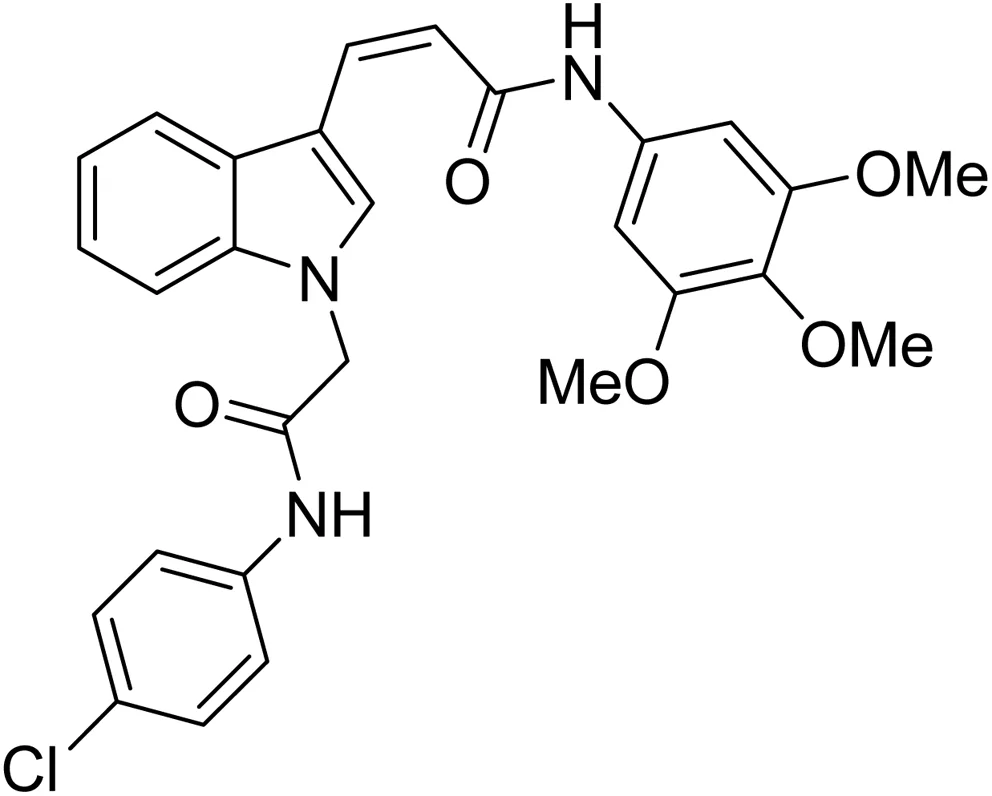


#### 4.2.12 3-(1-(2-((4-Chlorophenyl) amino)-2-oxoethyl)-1*H*-indol-3-yl)-*N*-(3,4,5-trimethoxyphenyl) acrylamide (10l)

Cream solid; yield: 86%; MP = 237–239; IR = 3257 (N–H, amide), 2930 (C–H, aliphatic), 1662 (C=O, amide), 1606 (C=C, alkene, aromatic), 1534 (N–H bend, amide II), 1458 (CH_2_ bend), 1407 (C–N, amide III), 1350 (C–N), 1287, 1232, 1181, 1131 (C–O–C, Ar–OMe) cm^−1^. ^1^H NMR (500 MHz, DMSO) *δ* 10.57 (s, 1H, NH_acetamide_), 9.96 (s, 1H, NH_trimethoxy_), 7.97 (d, *J* = 8.0 Hz, 1H, H4), 7.86 (s, 1H, H2), 7.72 (d, *J* = 15.8 Hz, 1H, H_2double bond_), 7.61 (d, *J* = 8.5 Hz, 2H, H2′, H6′), 7.52 (d, *J* = 8.0 Hz, 1H, H7), 7.37 (d, *J* = 8.5 Hz, 2H, H3′, H5′), 7.29–7.23 (m, 2H, H5, H6), 7.09 (s, 2H, H2″, H6″), 6.81 (d, *J* = 15.8 Hz, 1H, H_1double bond_), 5.13 (s, 2H, CH_2_), 3.77 (s, 6H, CH_3_), 3.62 (s, 3H, CH_3_), 13C NMR (126 MHz, DMSO) *δ* 165.94 (C=O), 164.72 (C=O), 152.74, 137.93, 137.56, 135.89, 134.83, 133.78, 133.16, 128.70, 127.15, 125.36, 122.59, 120.84, 120.71, 120.06, 116.69, 111.77, 110.75, 96.62 (C2″, C6″), 60.06 (CH_3_), 55.61 (CH_3_), 49.12 (CH_2_); anal. calcd: C_28_H_29_ClN_3_O_5_; C, 64.68; H, 5.04; N, 8.08; found: C, 64.95; H, 5.12; N, 8.19.
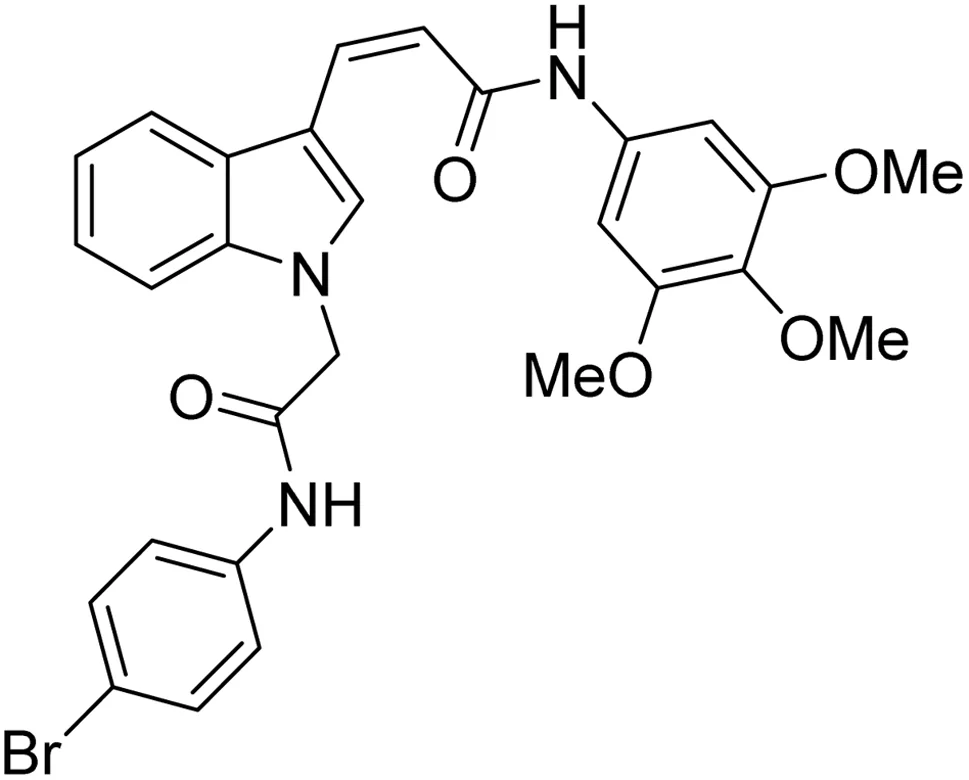


#### 4.2.13 3-(1-(2-((4-Bromophenyl) amino)-2-oxoethyl)-1*H*-indol-3-yl)-*N*-(3,4,5-trimethoxyphenyl) acrylamide (10m)

Cream solid; yield: 70%; MP = 240–242; IR: 3267 (N–H, amide), 3053 (=C–H, aromatic, vinyl), 2934 (C–H, aliphatic), 1678 (C=O, amide), 1607 (C=C, alkene, aromatic), 1533 (N–H amide II), 1298 (C–N), 1177, 1127 (C–O–C, Ar–OMe) cm^−1^.^1^H NMR (500 MHz, DMSO) *δ* 10.62 (s, 1H, NH_acetamide_), 10.00 (s, 1H, NH_trimethoxy_), 7.98 (d, *J* = 8.0 Hz, 1H, H4), 7.87 (s, 1H, H2), 7.73 (d, *J* = 15.8 Hz, 1H, H_2double bond_), 7.57 (d, *J* = 9.0 Hz, 2H, H2′, H6′), 7.53 (d, *J* = 8.0 Hz, 1H, H7), 7.50 (d, *J* = 9.0 Hz, 2H, H3′, H6′), 7.28–7.25 (m, 2H, H5, H6), 7.11 (s, 2H, H2″, H6″), 6.83 (d, *J* = 15.8 Hz, 1H, H_1double bond_), 5.14 (s, 2H, CH_2_), 3.77 (s, 6H, CH_3_), 3.63 (s, 3H, CH_3_); 13C NMR (126 MHz, DMSO) *δ* 165.96 (C=O), 164.74 (C=O), 152.75, 137.96, 137.94, 135.89, 134.81, 133.79, 133.21, 131.62, 125.38, 122.60, 121.10, 120.85, 120.08, 116.72, 115.19, 111.80, 110.75, 96.68 (C2″, C6″), 60.08 (CH_3_), 55.63 (CH_3_), 49.16 (CH_2_); anal. calcd: C_28_H_29_BrN_3_O_5_; C, 59.58; H, 4.64; N, 7.44; found: C, 59.71; H, 4.69; N, 7.52.
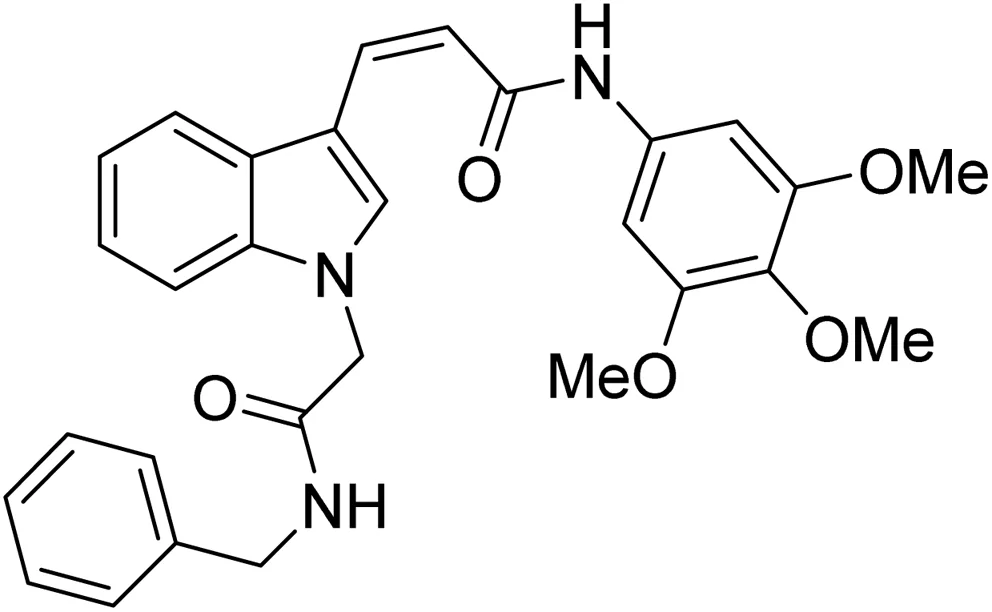


#### 4.2.14 3-(1-(2-(Benzylamino)-2-oxoethyl)-1*H*-indol-3-yl)-*N*-(3,4,5-trimethoxyphenyl) acrylamide (10n)

Cream solid; yield: 79%; MP = 238–240; IR (ATR) = 3265 (N–H, amide), 3067 (=C–H, aromatic), 2935 (C–H, aliphatic), 1658 (C=O, amide), 1605 (C=C, alkene, aromatic), 1540, 1504 (N–H bend, amide II), 1460 (CH_2_ bend), 1401 (C–N, amide III), 1319, 1281, 1207, 1131 (C–O–C, Ar–OMe) cm^−1^.^1^H NMR (500 MHz, DMSO) *δ* 9.99 (s, 1H, NH_trimethoxy_), 8.78 (t, *J* = 6.0 Hz, 1H, NH_acetamide_), 7.98 (d, *J* = 8.0 Hz, 1H, H4), 7.84 (s, 1H, H2), 7.74 (d, *J* = 15.8 Hz, 1H, H_2double bond_), 7.48 (d, *J* = 8.0 Hz, 1H, H7), 7.31 (d, *J* = 8.0 Hz, 2H, H2′, H6′), 7.29–7.27 (m, 2H, H5, H6), 7.27–7.26 (m, 3H, H5′, H3′, H4′), 7.12 (s, 2H, H2″, H6″), 6.83 (d, *J* = 15.8 Hz, 1H, H_1double bond_), 4.97 (s, 2H, CH_2_), 4.33 (d, *J* = 6.0 Hz, 2H, CH_2_), 3.78 (s, 6H, CH_3_), 3.64 (s, 3H, CH_3_); ^13^C NMR (126 MHz, DMSO) *δ* 166.91 (C=O), 164.69 (C=O), 152.73, 138.89, 137.76, 135.87, 134.81, 133.80, 133.17, 128.25, 127.27, 126.86, 125.42, 122.48, 120.81, 120.05, 116.55, 111.65, 110.71, 96.62 (C2″, C6″), 60.07 (CH_3_), 55.63 (CH_3_), 48.72 (CH_2_), 42.31 (CH_2_); anal. calcd: C_29_H_29_N_3_O_5_; C, 69.72; H, 5.85; N, 8.41; found: C, 69.81; H, 5.93; N, 8.47.
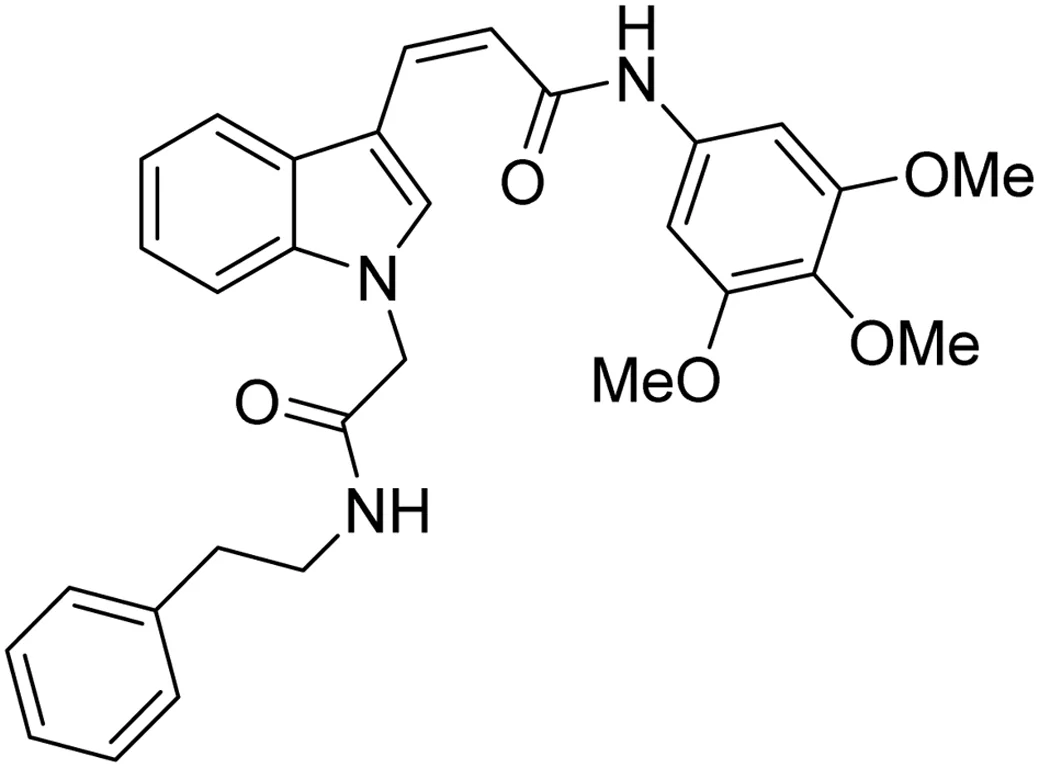


#### 4.2.15 3-(1-(2-Oxo-2-(phenethylamino) ethyl)-1*H*-indol-3-yl)-*N*-(3,4,5-trimethoxyphenyl) acrylamide (10o)

Cream solid; yield: 89%; MP = 235–237; IR (ATR): = 3290 (N–H, amide), 2933 (C–H, aliphatic), 1654 (C=O, amide), 1605 (C=C, alkene, aromatic), 1511 (N–H bend, amide II), 1459, 1408 (CH_2_ bend), 1318 (C–N, amide III), 1222, 1129 (C–O–C, Ar–OMe) cm^−1^.^1^H NMR (500 MHz, DMSO) *δ* 9.99 (s, 1H, NH_trimethoxy_), 8.33 (t, *J* = 6.0 Hz, 1H, NH_acetamide_), 7.98 (d, *J* = 8.0 Hz, 1H, H4), 7.78 (s, 1H, H2), 7.74 (d, *J* = 15.8 Hz, 1H, H_2double bond_), 7.38 (d, *J* = 8.0 Hz, 1H, H7), 7.29–7.27 (m, 4H, H2′, H3′, H5′, H6′), 7.21–7.18 (m, 3H, H4′, H5, H6), 7.12 (s, 2H, H2′, H6′), 6.83 (d, *J* = 15.8 Hz, 1H, H_1double bond_), 4.86 (s, 2H, CH_2_), 3.78 (s, 6H, CH_3_), 3.64 (s, 3H, CH_3_), 3.38–3.30 (m, 2H, CH_2_), 2.74 (t, *J* = 7.0 Hz, 2H, CH_2_); ^13^C NMR (126 MHz, DMSO) *δ* 166.70 (C=O), 164.70 (C=O), 152.74, 139.19, 137.69, 135.88, 134.75, 133.80, 133.17, 128.62, 128.26, 126.07, 125.38, 122.48, 120.79, 120.02, 116.52, 111.58, 110.70, 96.62 (C2″, C6″), 60.07 (CH_3_), 55.63 (CH_3_), 48.73 (CH_2_), 34.91(CH_2_); anal. calcd: C_30_H_31_N_3_O_5_; C, 70.16; H, 6.08; N, 8.18; found: C, 70.32; H, 6.11; N, 8.29.

### 4.3 Cell culture

Breast cancer cell lines MCF-7 and MDA-MB-468, the hepatocellular carcinoma cell line Hep-G2, and HEK-293 as a normal cell line were obtained from the Iranian Biological Resource Center (Tehran, Iran). Cells were cultured in high-glucose DMEM supplemented with 10% heat-inactivated fetal bovine serum (FBS) and antibiotics (penicillin/streptomycin). All cell lines were maintained as monolayer cultures at 37 °C in a humidified incubator containing 5% CO_2_.

### 4.4 MTT cell viability assay

The cytotoxic effects of the synthesized compounds were evaluated using the MTT [3-(4,5-dimethylthiazol-2-yl)-2,5-diphenyltetrazolium bromide] assay. Cells were seeded into 96-well microplates at a density of 7000 cells per well (100 µL per well) and incubated overnight at 37 °C. Subsequently, 50 µL of the medium in each well was replaced with the same volume of fresh medium containing different concentrations of the test compounds. Test compounds were first dissolved in DMSO and then diluted in culture medium, ensuring that the final DMSO concentration did not exceed 0.5% in any well.^23^ After 72 h of incubation, 80 µL of the medium was replaced with 80 µL of MTT solution (0.5 mg mL^−1^ in medium), and the cells were incubated for an additional 4 h at 37 °C. The supernatant was then removed, and 200 µL of DMSO was added to each well to dissolve the formazan crystals formed by viable cells. The plates were incubated for 1 h and shaken for an additional 30 min to ensure complete dissolution. The optical density of each well was measured at 570 nm using a microplate reader. The IC_50_ values of the synthesized derivatives were determined from three independent experiments using CurveExpert 1.34 software^24^ and are expressed as mean ± standard deviation.

### 4.5 Cell cycle analysis

The MCF-7 cells (2.5 × 10^4^ cells per well) were treated with 10b at final concentrations of 7, 15 and 50 µM for 72 h, then cells were harvested with 2.5 mL of ice-cooled 70% ethanol for 2 hours. Cells were precipitated at 4000 rpm for 2 minutes, followed by resuspension in 500 µL PBS containing 0.25% Triton X-100 and incubation on ice for 15 minutes. Cells were precipitated again at 4000 rpm for 2 minutes. The cells were finally suspended in 500 µL PBS containing 10 µg mL^−1^ RNase A and 20 µg mL^−1^ propidium iodide (PI) and incubated for 30 minutes in the dark. The cells were finally analyzed using BD FACS Calibur flow cytometry.^25^

### 4.6 Assessment of apoptosis/necrosis in MCF-7 cells

The ability of 10b at final concentrations of 15 and 50 µM to induce apoptosis or necrosis was evaluated using an Annexin V-FITC/propidium iodide (PI) staining kit (BD Pharmingen, San Diego, CA, USA). To eliminate the potential effect of the DMSO vehicle, the compound was diluted 400-fold in culture medium, resulting in a final DMSO concentration of 0.25% (v/v). MCF-7 cells were cultured in 6-well plates at a density of 2.5 × 10^4^ cells per well. After 24 h, the synthesized derivatives at different concentrations were added and incubated for 24 and 72 h. After adding trypsin to each well, the cells were harvested, transferred to 1.5 ml tubes, and washed twice with PBS. In the final step, the cells were stained with Annexin V-FITC (5 µl) and PI (5 µl), then analyzed by flow cytometry.^26^

### 4.7 Statistical analysis

Statistical analysis was performed using one-way ANOVA in GraphPad Prism. Data are expressed as the mean ± standard deviation (SD).

## Authors contributions

S. N. G. and M. M. synthesized compounds and contributed to the characterization of compounds. A. I. supervised the *in silico* and biological part of the study. P. R. R. supervised the synthetic part of the study and interpretation of ^1^H NMR and ^13^C NMR spectra. All authors read and approved the final version of the article.

## Conflicts of interest

There are no conflicts to declare.

## Abbreviations

DCC: *N*,*N*′-Dicyclohexylcarbodiimide
DIEA: *N*,*N*-Diisopropylethylamine
DMF: Dimethylformamide
DMSO: Dimethyl sulfoxide
EDC: 1-Ethyl-3-(3-dimethylaminopropyl)carbodiimide
FITC: Fluorescein isothiocyanate
HCl: Hydrochloric acid
PBS: Phosphate-buffered saline
SAR: Structure–activity relationship
TBTU: 2-(1*H*-Benzotriazole-1-yl)-1,1,3,3-tetramethylaminium tetrafluoroborate
TLC: Thin-layer chromatography
TNBC: Triple-negative breast cancer
TPSA: Total polar surface area

## Supplementary Material

RA-OLF-D6RA03896D-s001

## Data Availability

The datasets generated and/or analyzed during the current study are available in the Worldwide Protein Data Bank (wwPDB) repository (https://www.rcsb.org/structure/4O2B). Supplementary information (SI): containing ^1^H NMR, ^13^C NMR spectra, and *in silico* studies. See DOI: https://doi.org/10.1039/d6ra03896d.
