# Supplementary material for: Design and synthesis of 3-(1H-indol-3-yl)-N-(3,4,5-trimethoxyphenyl) acrylamide derivatives as anticancer agents
Source: RSC Adv. 2026 Oct 22. Online ahead of print. doi: 10.1039/d6ra03896d (PMC13688103; doi:10.1039/d6ra03896d)
Supplement: Supplementary file 1 [file RA-OLF-D6RA03896D-s001.pdf]

Supplementary Information (SI) for RSC Advances.
This journal is © The Royal Society of Chemistry

Design and synthesis of 3-(1H-indol-3-yl)-N-(3,4,5-trimethoxyphenyl) acrylamide derivatives as anticancer

Seyedeh Niloufar Ghafouri[1], Parviz Rashidi Ranjbar[1*], Mohammad Mahdavi[2], Aida Iraji[3,4*]

1. School of Chemistry, College of Science, University of Tehran, Tehran, Iran
2. Endocrinology and Metabolism Research Center, Endocrinology and Metabolism Clinical Sciences Institute, Tehran University of Medical Sciences, Tehran, Iran.
3. Stem Cells Technology Research Center, Shiraz University of Medical Sciences, Shiraz, Iran
4. Pharmaceutical Sciences Research Center, Shiraz University of Medical Sciences, Shiraz, Iran

*Figure S1.[1] H NMR (500 MHz, DMSO-d_6) spectrum of **10a***

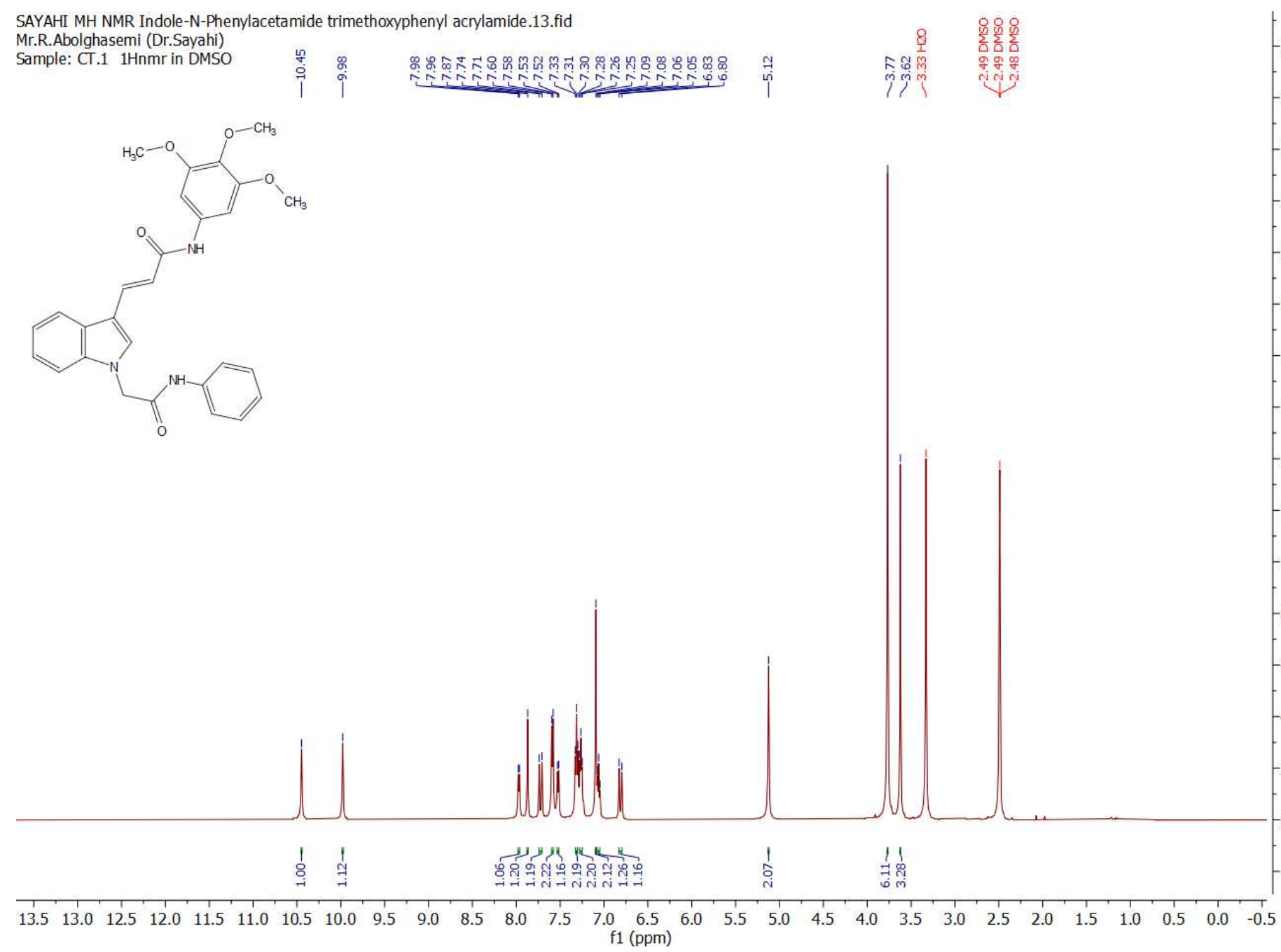

*Figure S2.[13] C{[1] H} NMR (126 MHz, DMSO-d_6) spectrum of **10a***

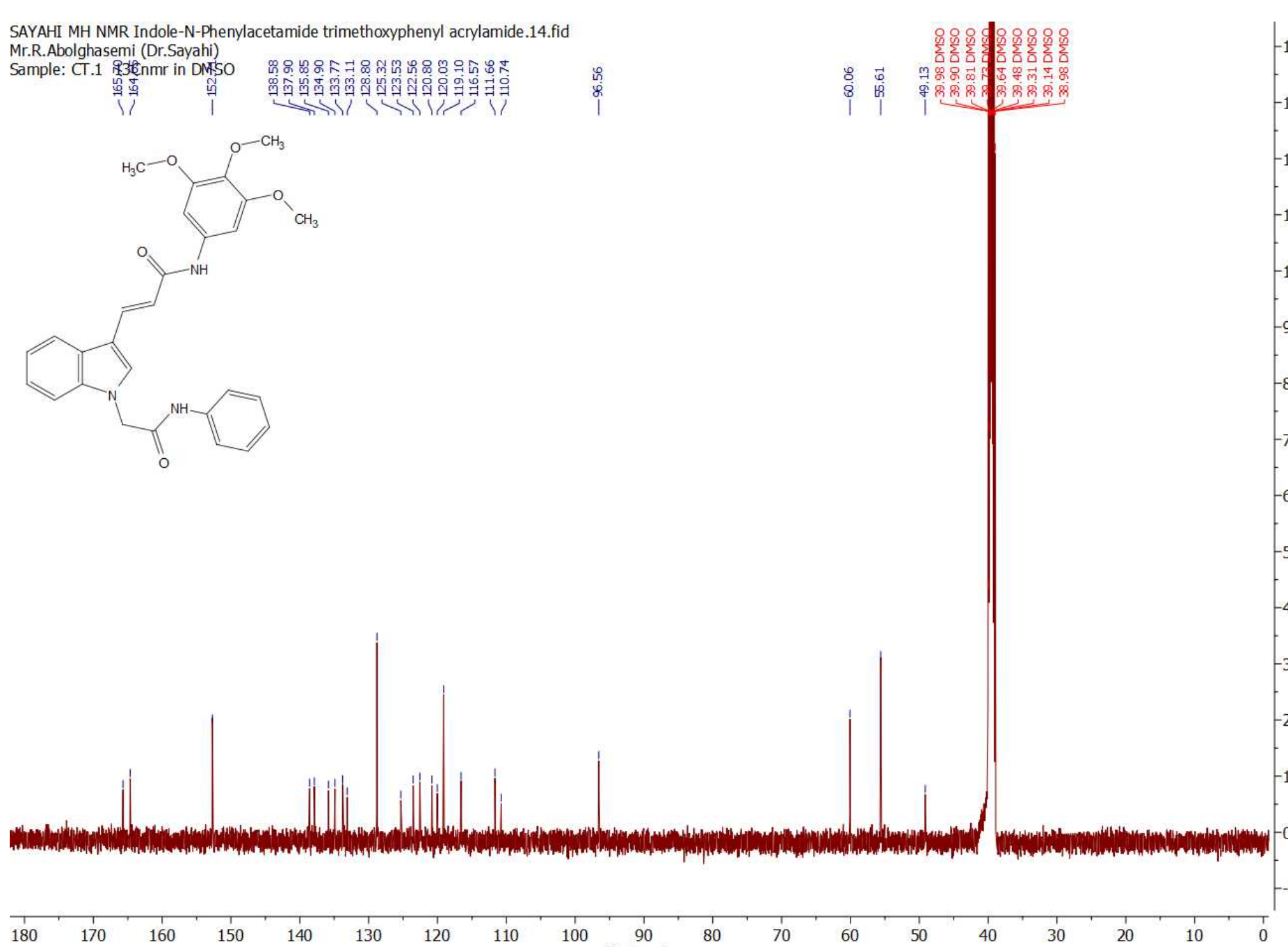

*Figure S3. IR Spectral Data (ATR-FTIR) of **10a***

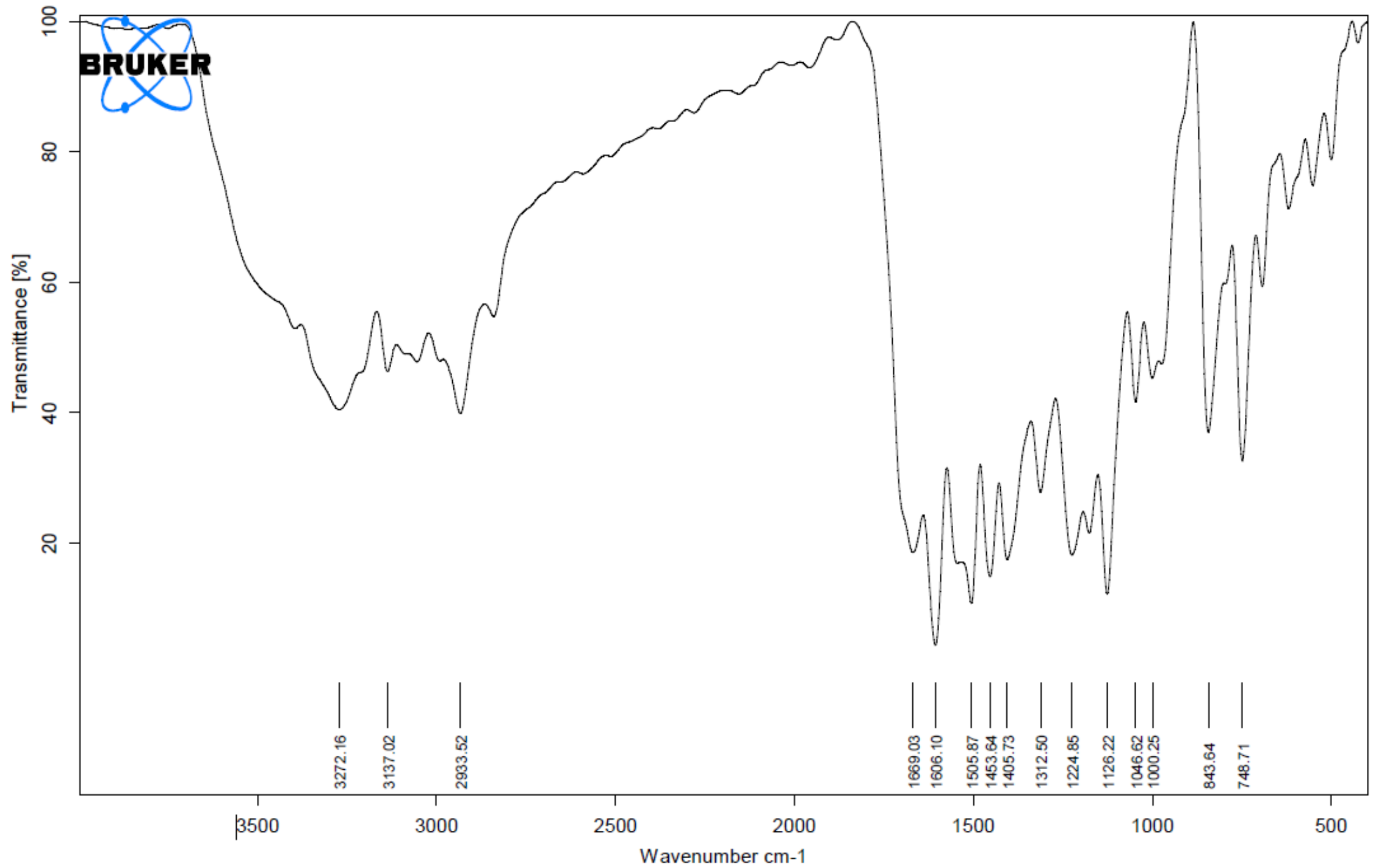

*Figure S4. HPLC Purity Analysis of **10a***

Empower 3

CT Related

SAMPLE INFORMATION

Sample Name:	CT1	Acquired By:	System
Sample Type:	Control	Sample Set Name:	CT
Vial:	12	Acq. Method Set:	CT
Injection #:	1	Processing Method:	1
Injection Volume:	20.00 ul	Channel Name:	W2489 ChA
Run Time:	37.0 Minutes	Proc. Chnl. Descr.:	W2489 ChA 230nm
Date Acquired:	1/18/2026 8:02:06 PM GST		
Date Processed:	6/21/2026 1:53:36 PM GST		

Auto-Scaled Chromatogram

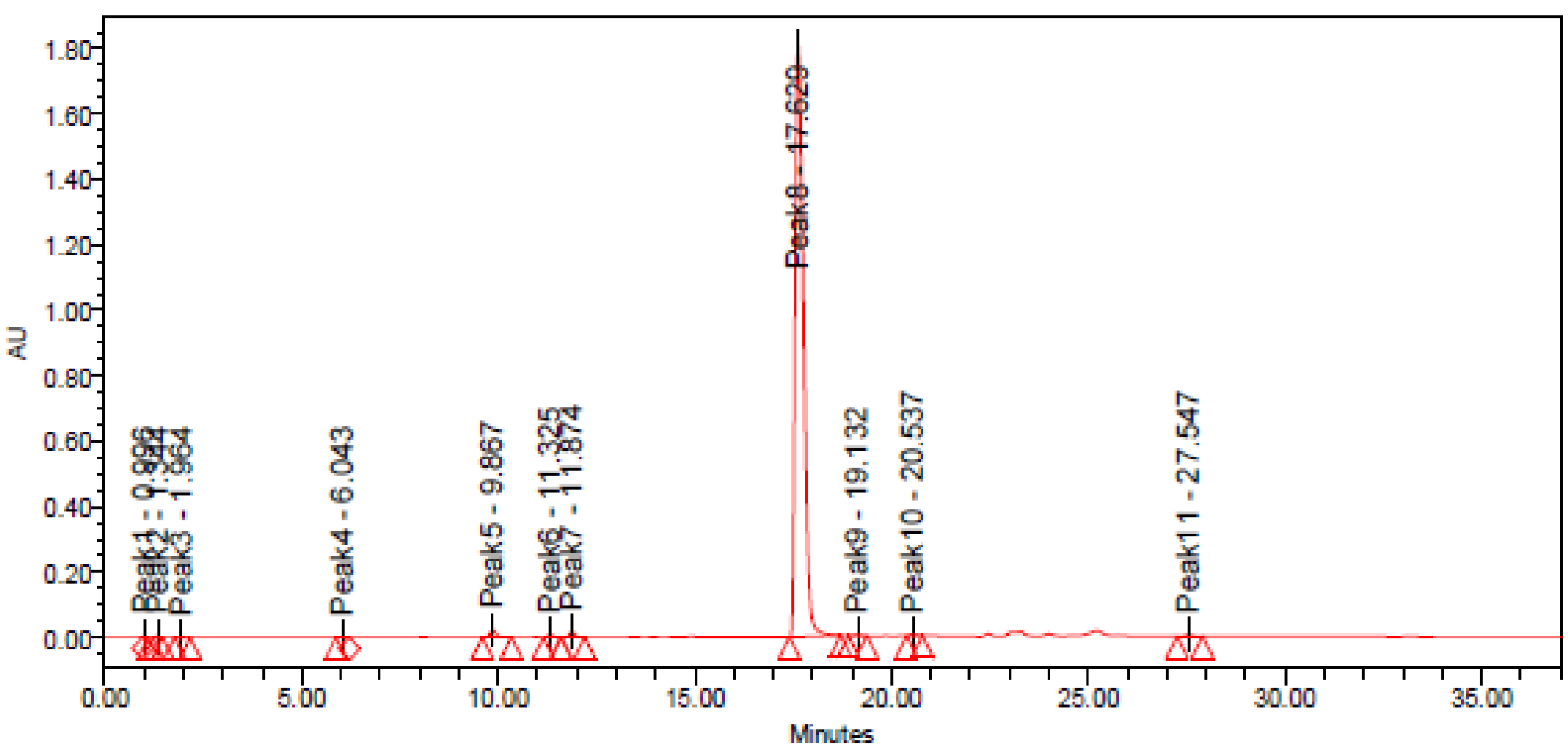

	Peak Name	Retention Time (min)	Height	Area	% Area
1	Peak1	0.996	2748	14566	0.05
2	Peak2	1.344	4693	25550	0.09
3	Peak3	1.964	877	7625	0.03
4	Peak4	6.043	1364	13771	0.05
5	Peak5	9.867	22184	243673	0.90
6	Peak6	11.325	8315	75721	0.28
7	Peak7	11.874	17430	163688	0.60
8	Peak8	17.629	1807324	26495617	97.44
9	Peak9	19.132	2509	32209	0.12
10	Peak10	20.537	5053	51973	0.19
11	Peak11	27.547	5339	68916	0.25

Reported by User: System
Report Method: Chemi
Report Method ID: 1 11636
Page: 1 of 1

Project Name: CT
Date Printed:
6/21/2026
1:54:56 PM Asia/Dubai

Figure S5. ^{1}H NMR (500 MHz, DMSO-d_6) spectrum of ***10b***

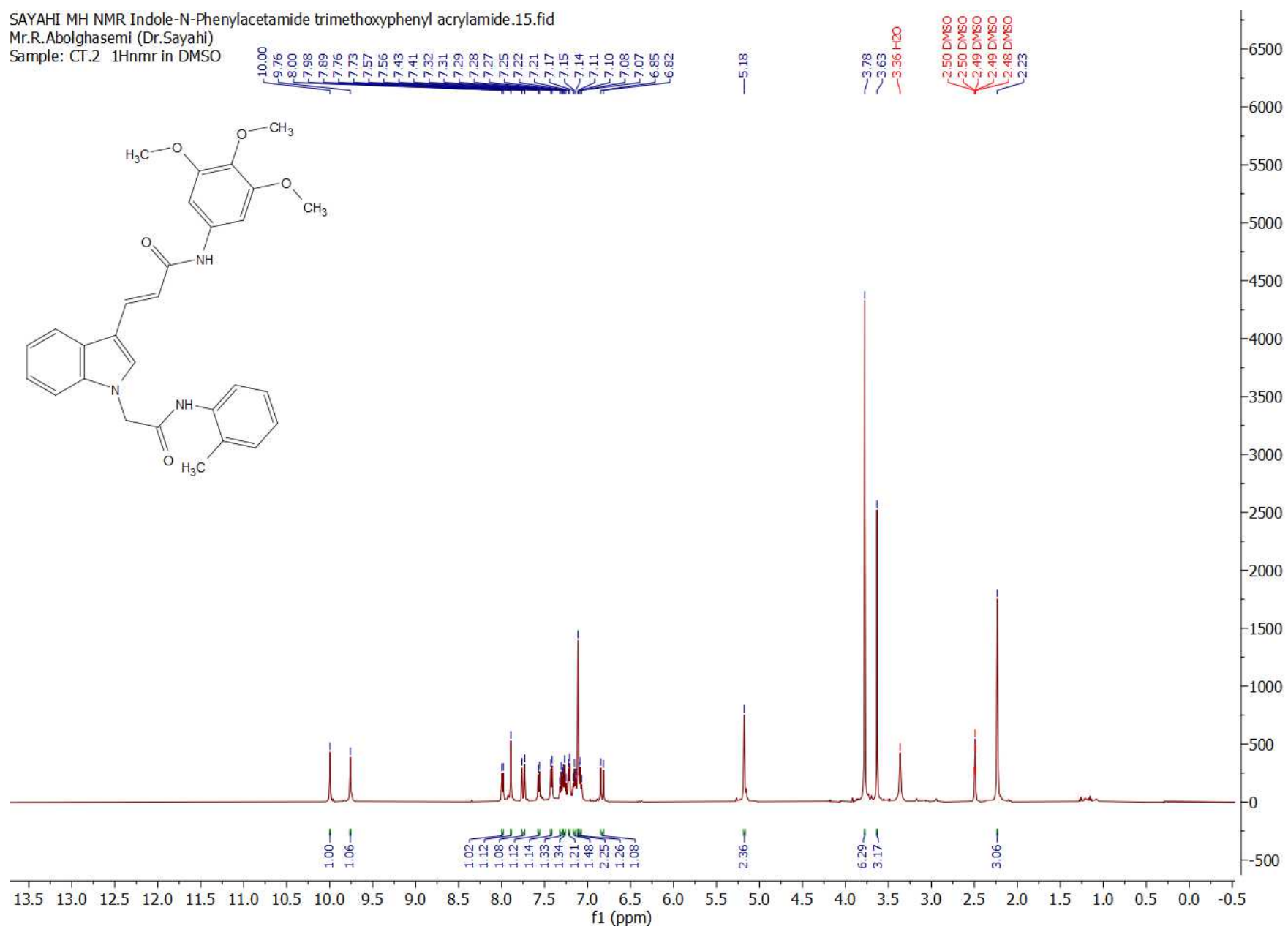

Figure S6.^{13}C{^{1}H} NMR (126 MHz, DMSO-d_6) spectrum of ***10b***

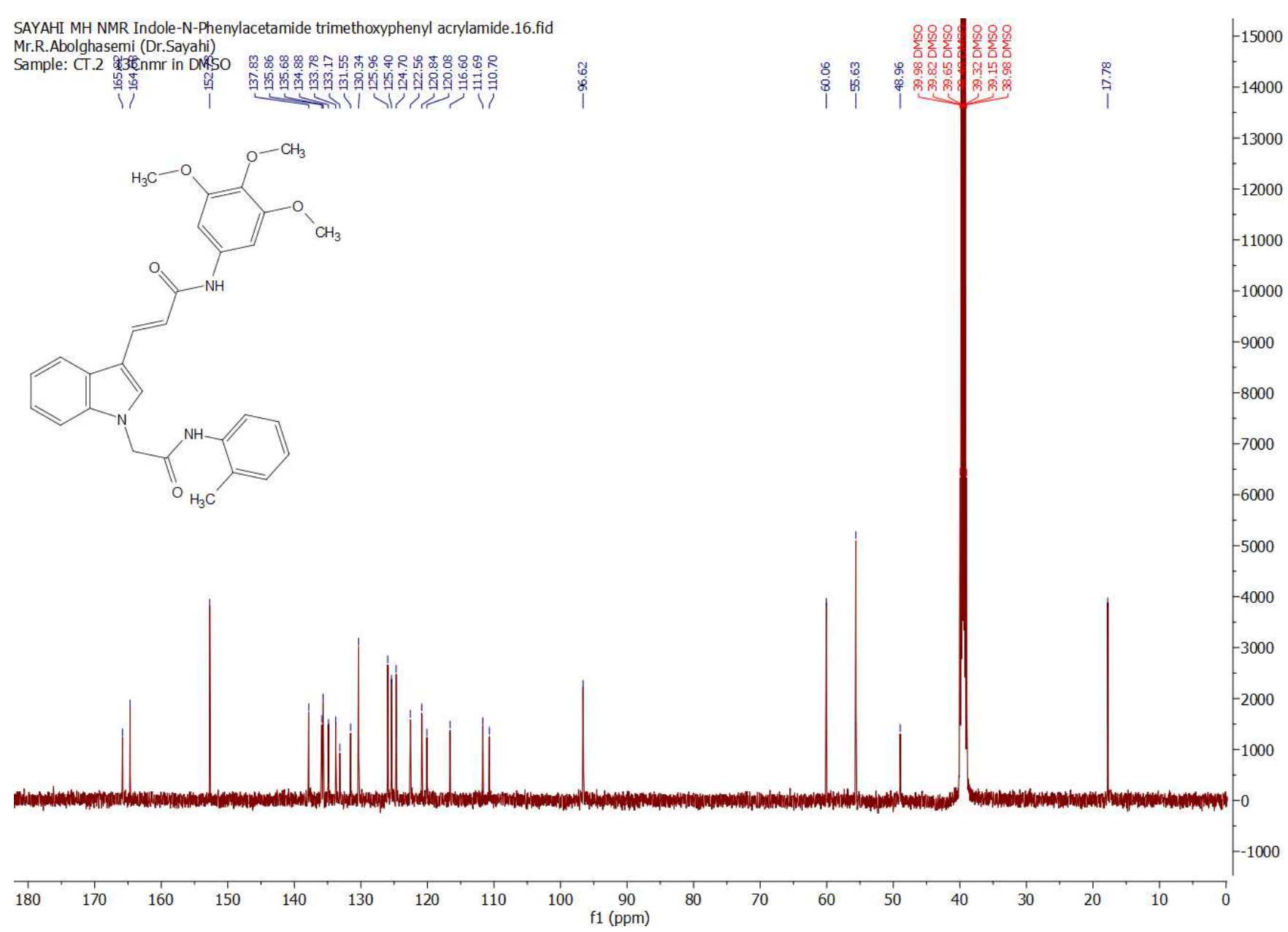

Figure S7. IR Spectral Data (ATR-FTIR) of ***10b***

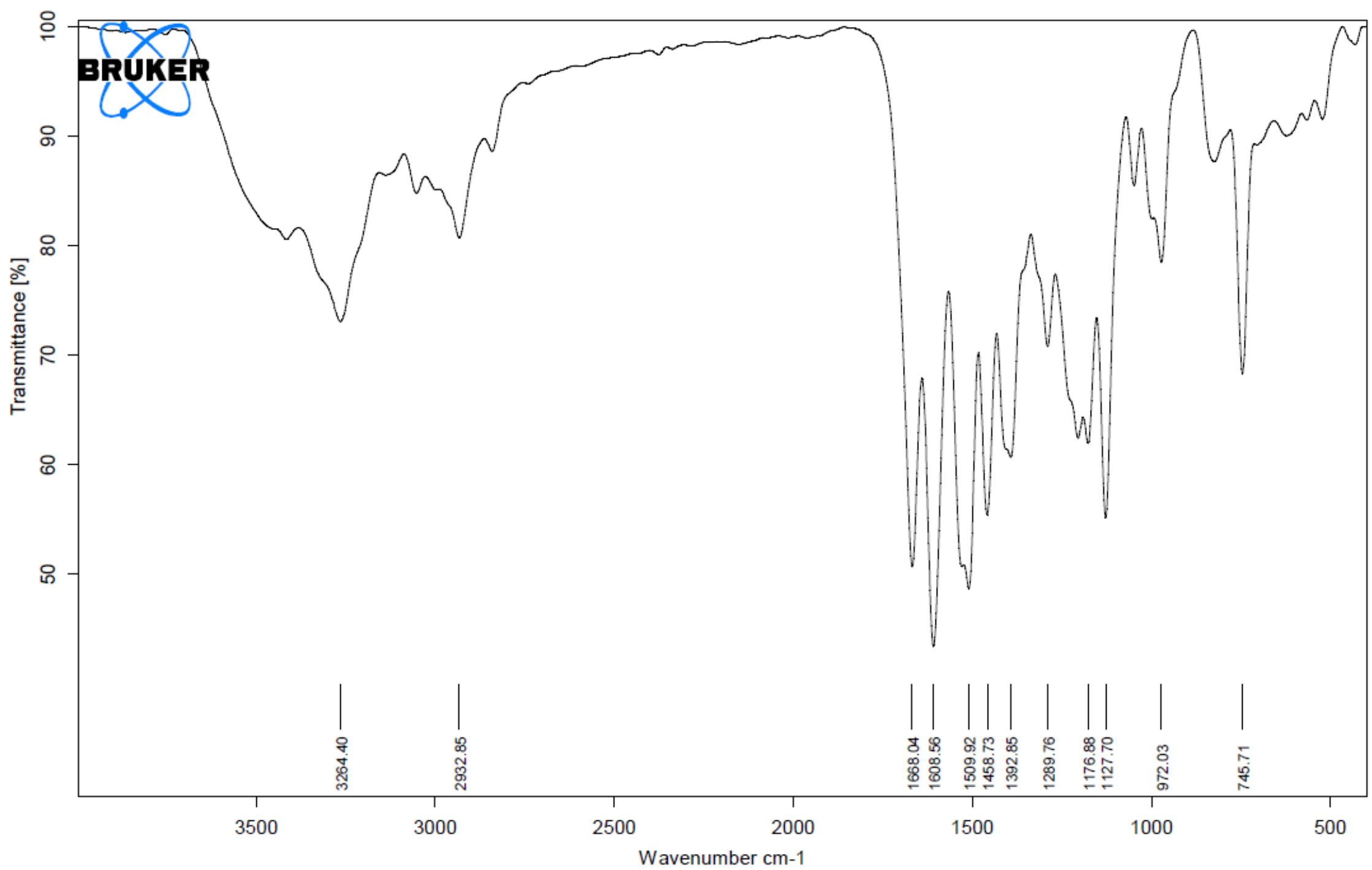

*Figure S8. HPLC Purity Analysis of **10b***

Empower 3 SOFTWARE

CT - Related

SAMPLE INFORMATION

Sample Name:	CT2	Acquired By:	System
Sample Type:	Control	Sample Set Name:	CT
Vial:	13	Acq. Method Set:	CT
Injection #:	1	Processing Method:	1
Injection Volume:	20.00 ul	Channel Name:	W2489 ChA
Run Time:	37.0 Minutes	Proc. Chnl. Descr.:	W2489 ChA 230nm
Date Acquired:	1/18/2026 8:39:59 PM GST		
Date Processed:	6/21/2026 1:51:09 PM GST		

Auto-Scaled Chromatogram

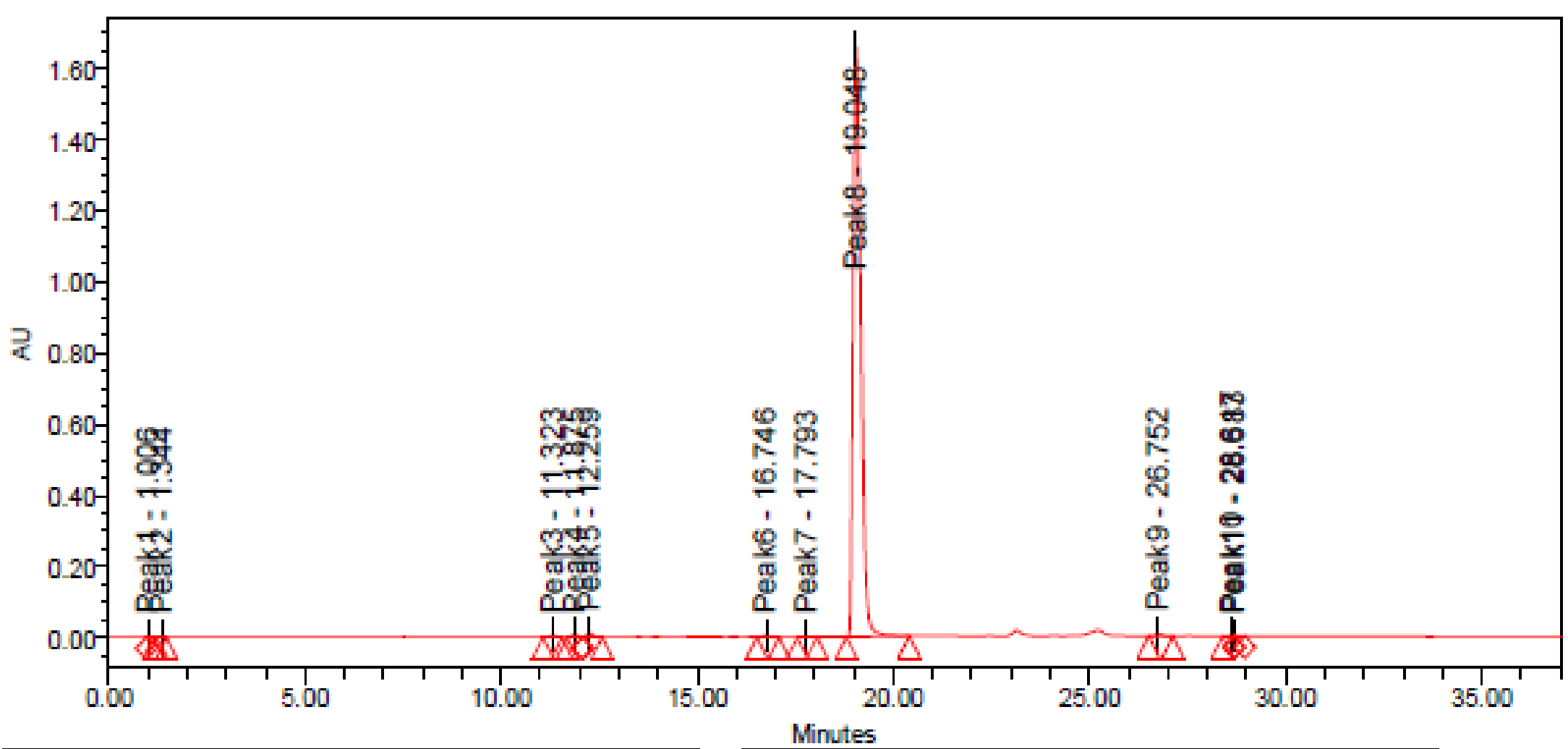

	Peak Name	Retention Time (min)	Height	Area	% Area
1	Peak1	1.006	2631	17314	0.07
2	Peak2	1.344	2995	16686	0.07
3	Peak3	11.323	6407	58102	0.25
4	Peak4	11.875	8707	79482	0.34
5	Peak5	12.259	11501	120939	0.51
6	Peak6	16.746	4696	55428	0.23
7	Peak7	17.793	1734	16410	0.07
8	Peak8	19.048	1657641	23155801	97.94
9	Peak9	26.752	7397	83225	0.35
10	Peak10	28.617	2363	22911	0.10
11	Peak11	28.683	2090	15832	0.07

Reported by User: System
Report Method: Chemi
Report Method ID: 1 11636
Page: 1 of 1

Project Name: CT
Date Printed:
6/21/2026
1:56:11 PM Asia/Dubai

Figure S9. HRMS Spectra of ***10b***

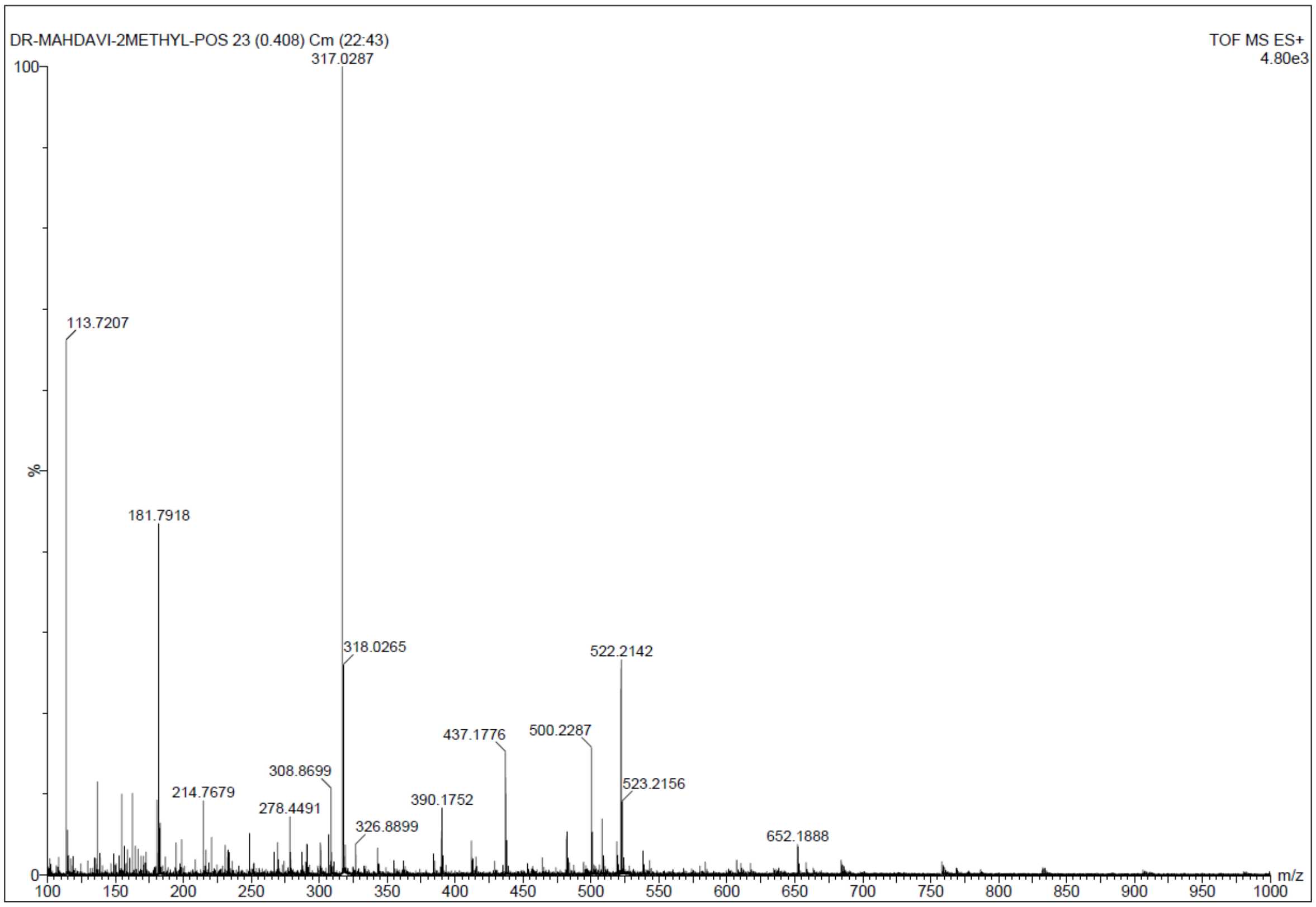

Figure S10.^{1}H NMR (500 MHz, DMSO-d_6) spectrum of ***10c***

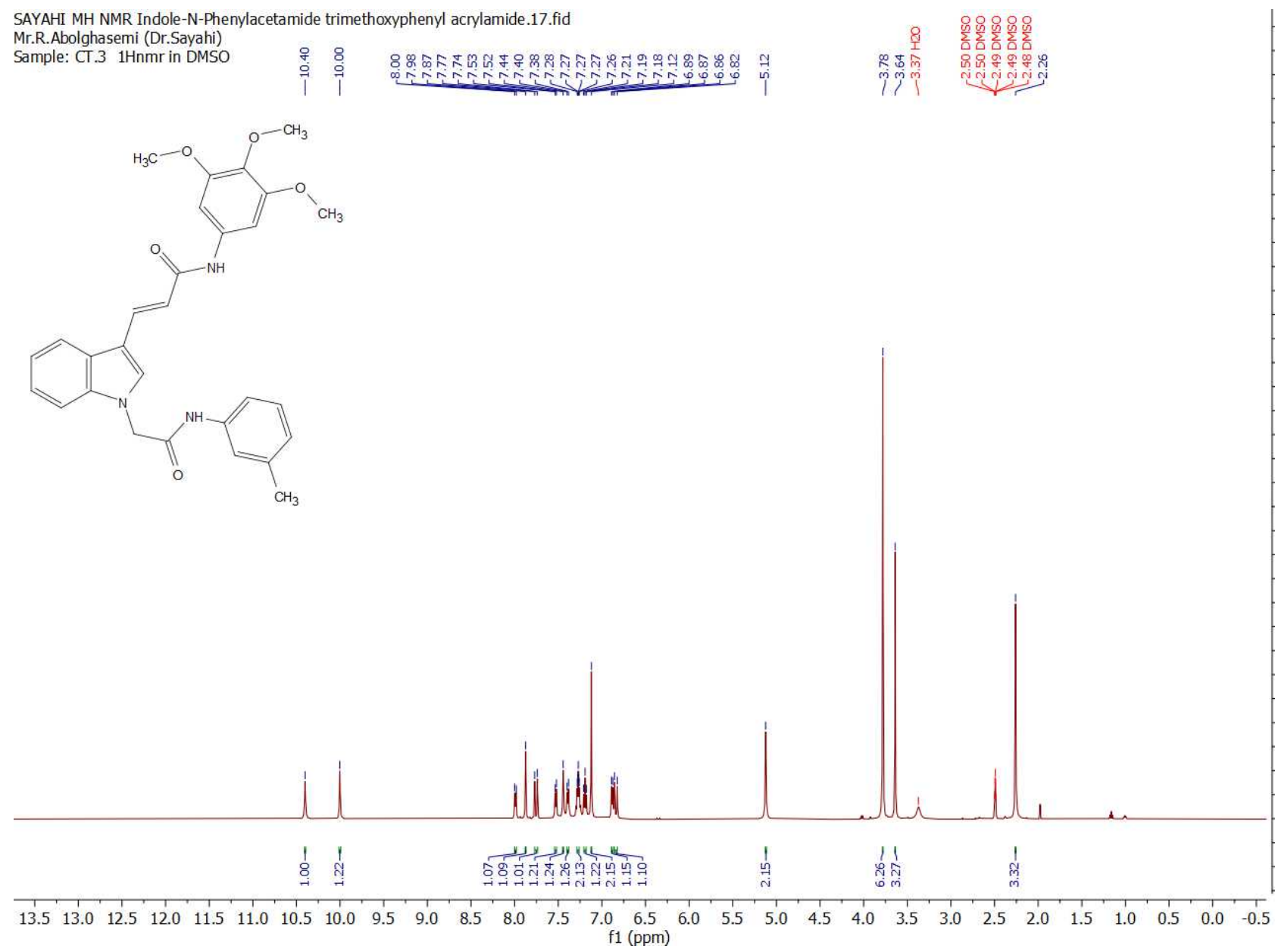

Figure S11.^{13}C{^{1}H} NMR (126 MHz, DMSO-d_6) spectrum of ***10c***

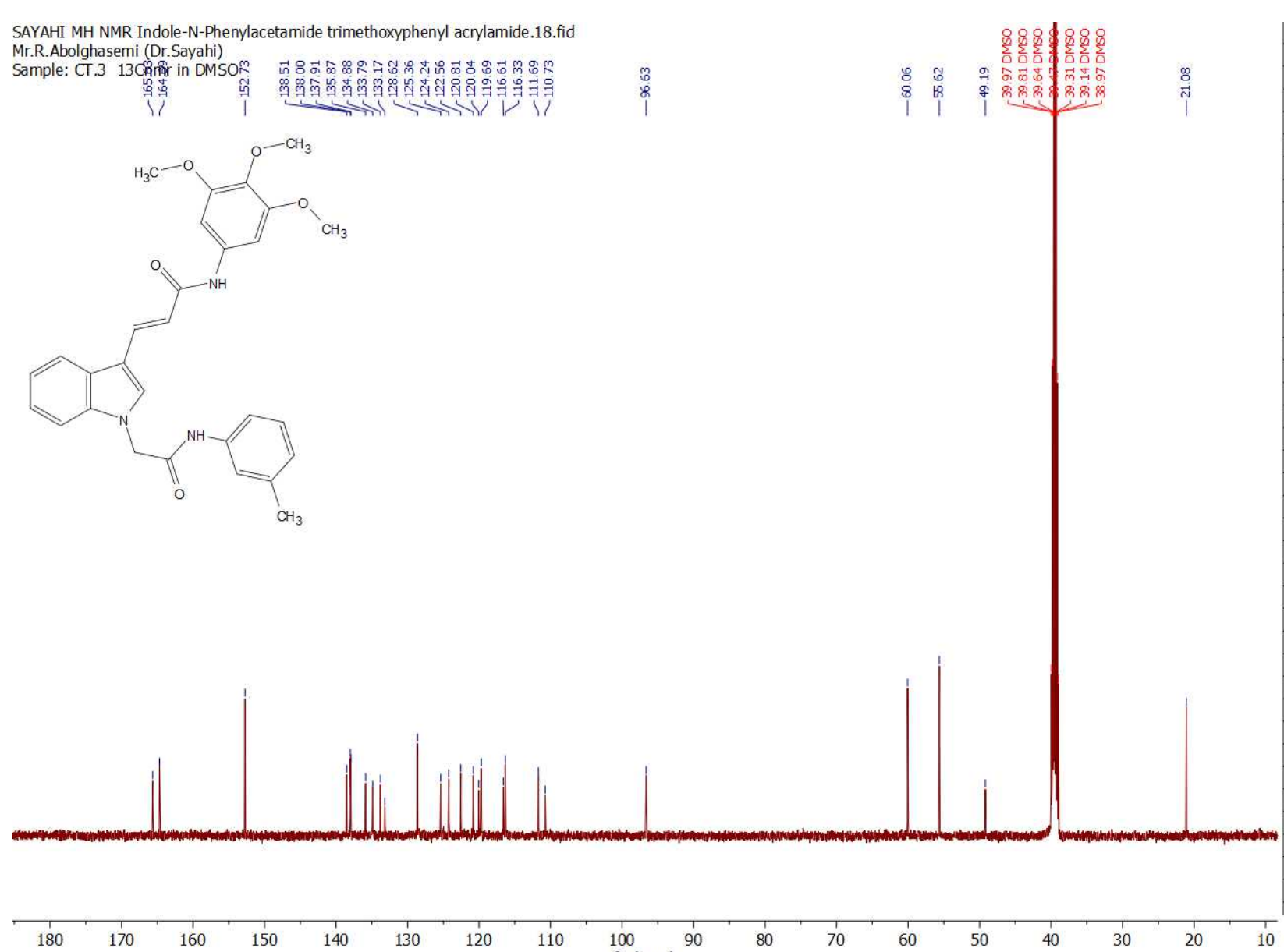

*Figure S12. IR Spectral Data (ATR-FTIR) of **10c***

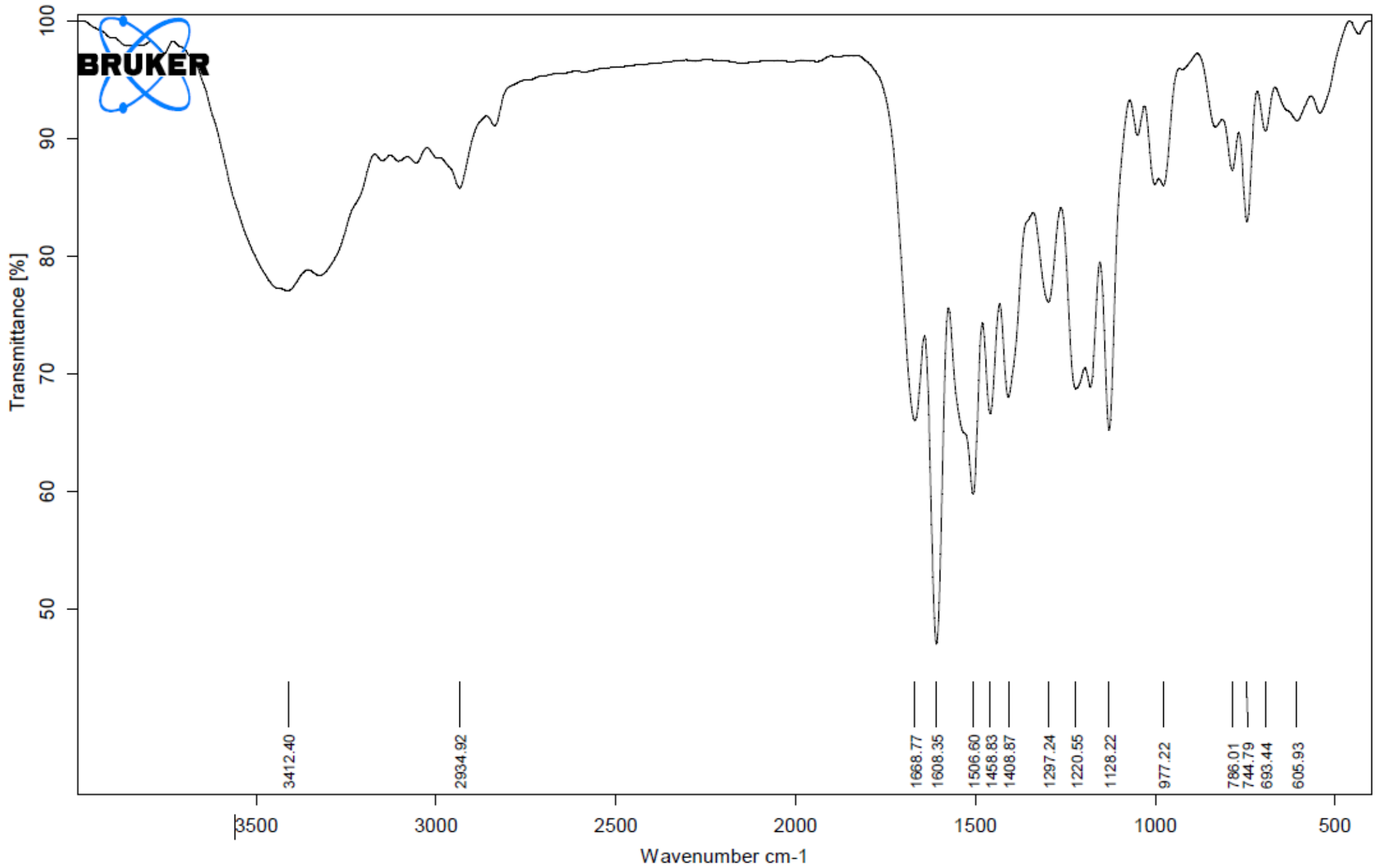

*Figure S13.^{1}H NMR (500 MHz, DMSO-d_6) spectrum of **10d***

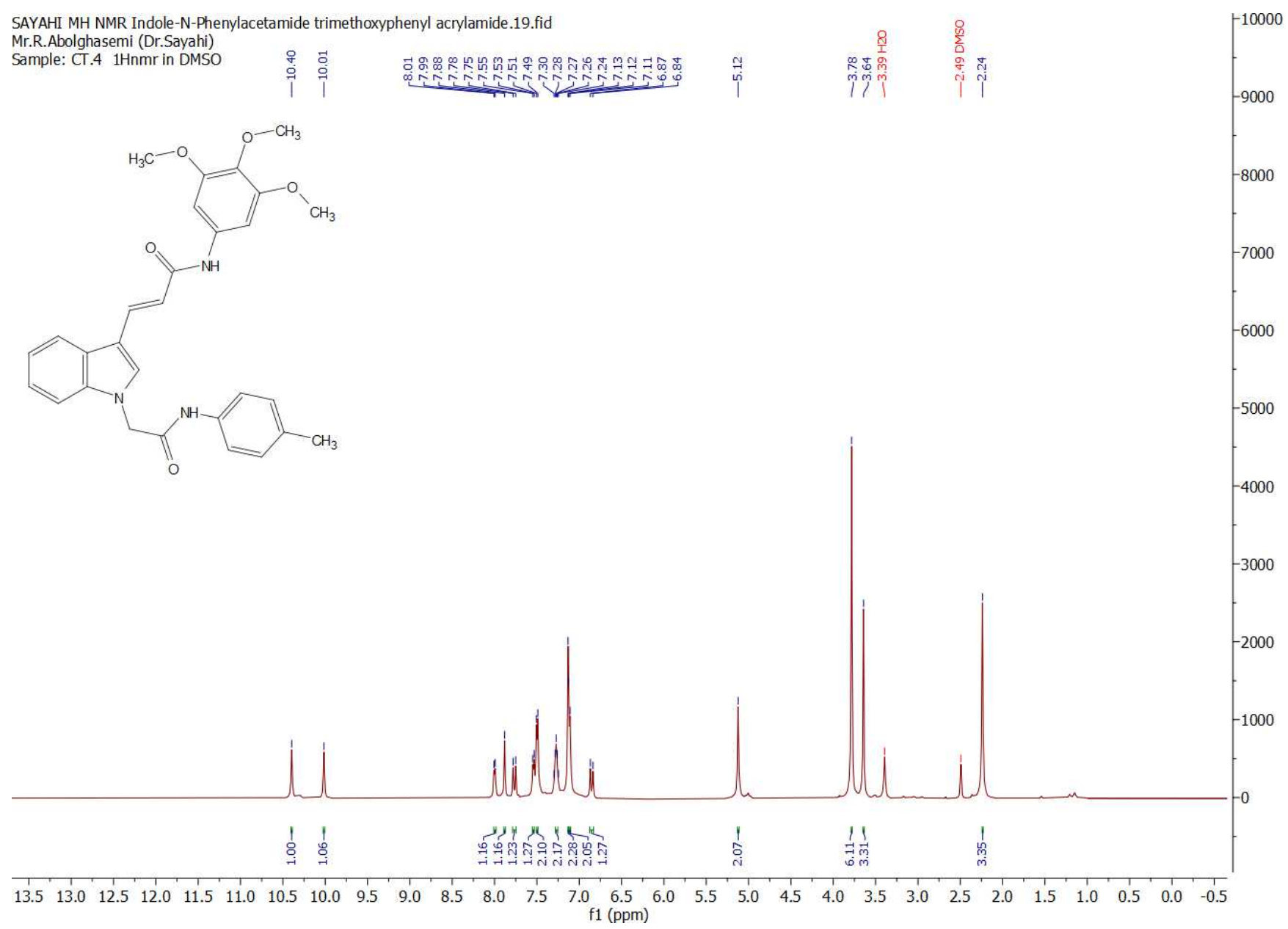

*Figure S14.^{13}C{^{1}H} NMR (126 MHz, DMSO-d_6) spectrum of **10d***

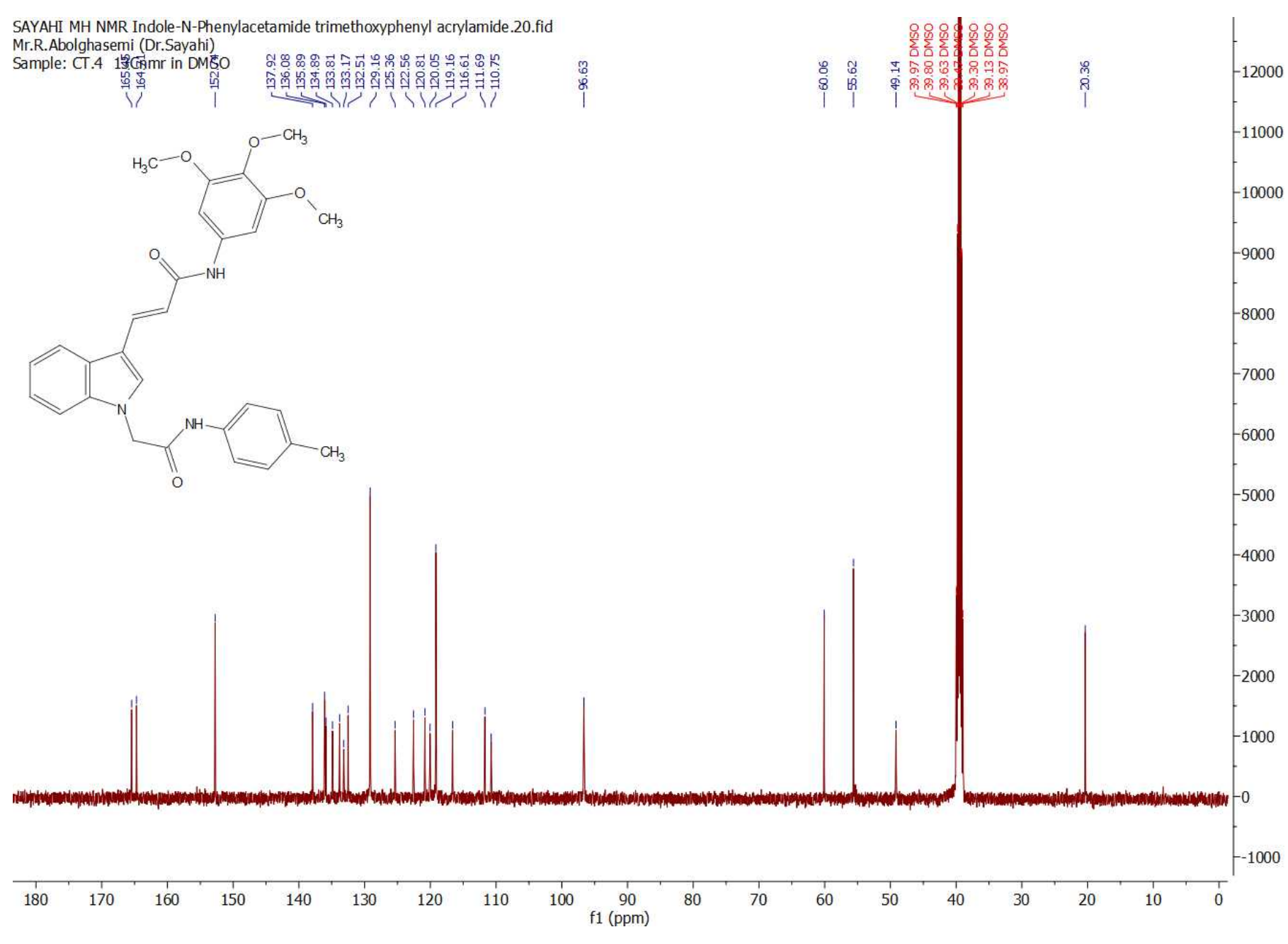

Figure S15. IR Spectral Data (ATR-FTIR) of ***10d***

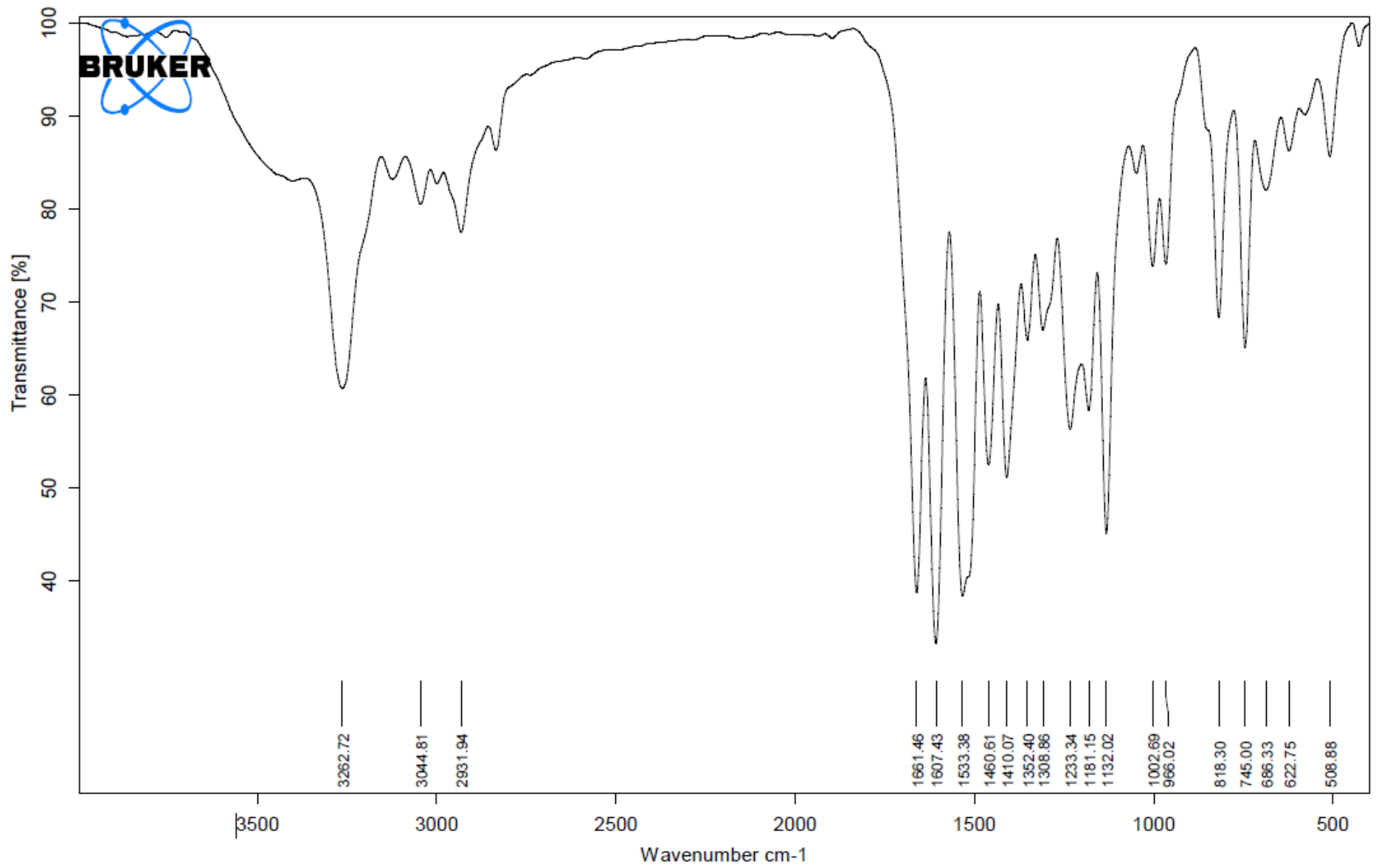

*Figure S16.^{1}H NMR (500 MHz, DMSO-d_6) spectrum of **10e***

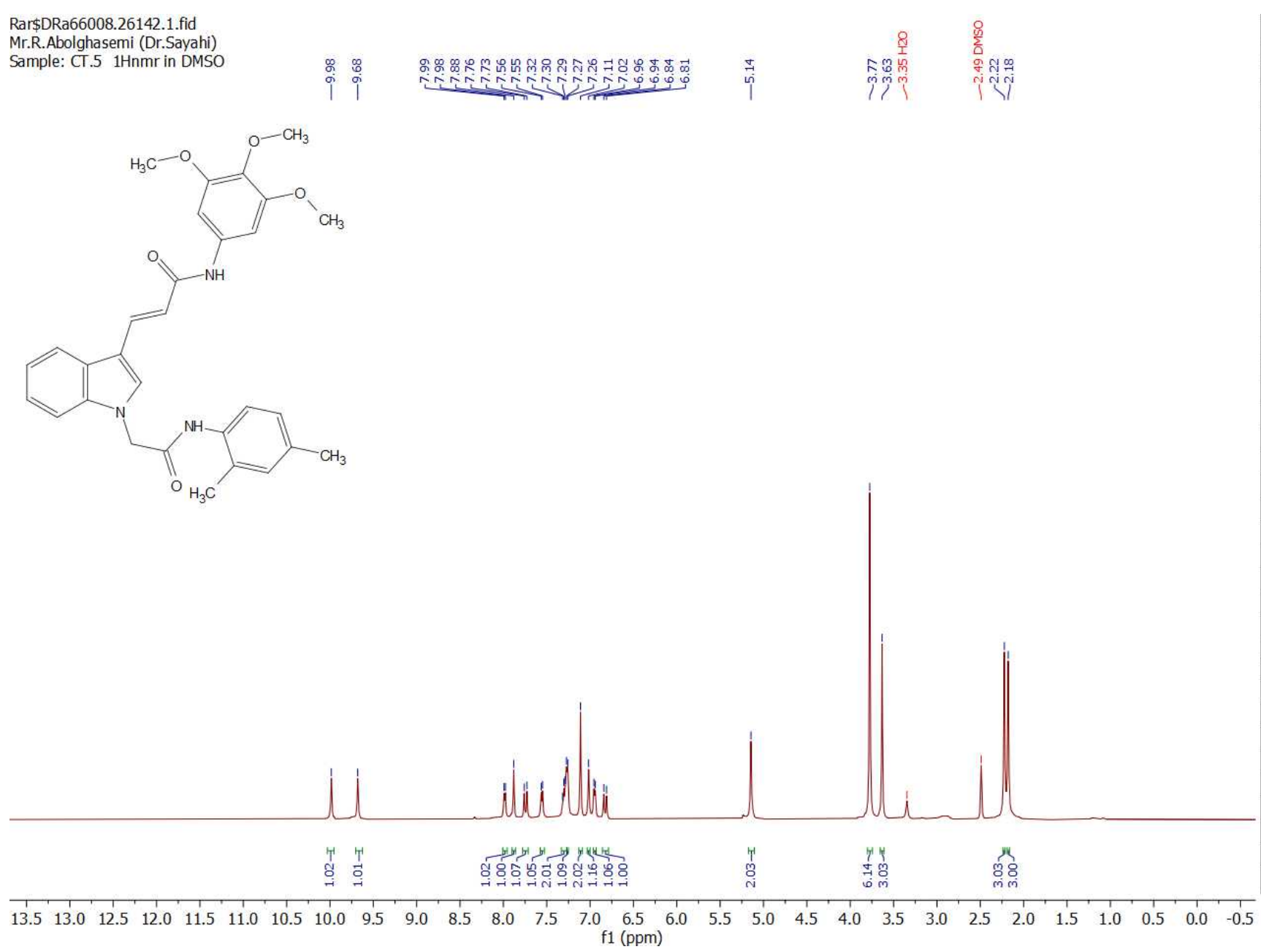

*Figure S17. $^{13}C\{^{1}H\}$ NMR (126 MHz, DMSO-d_6) spectrum of **10e***

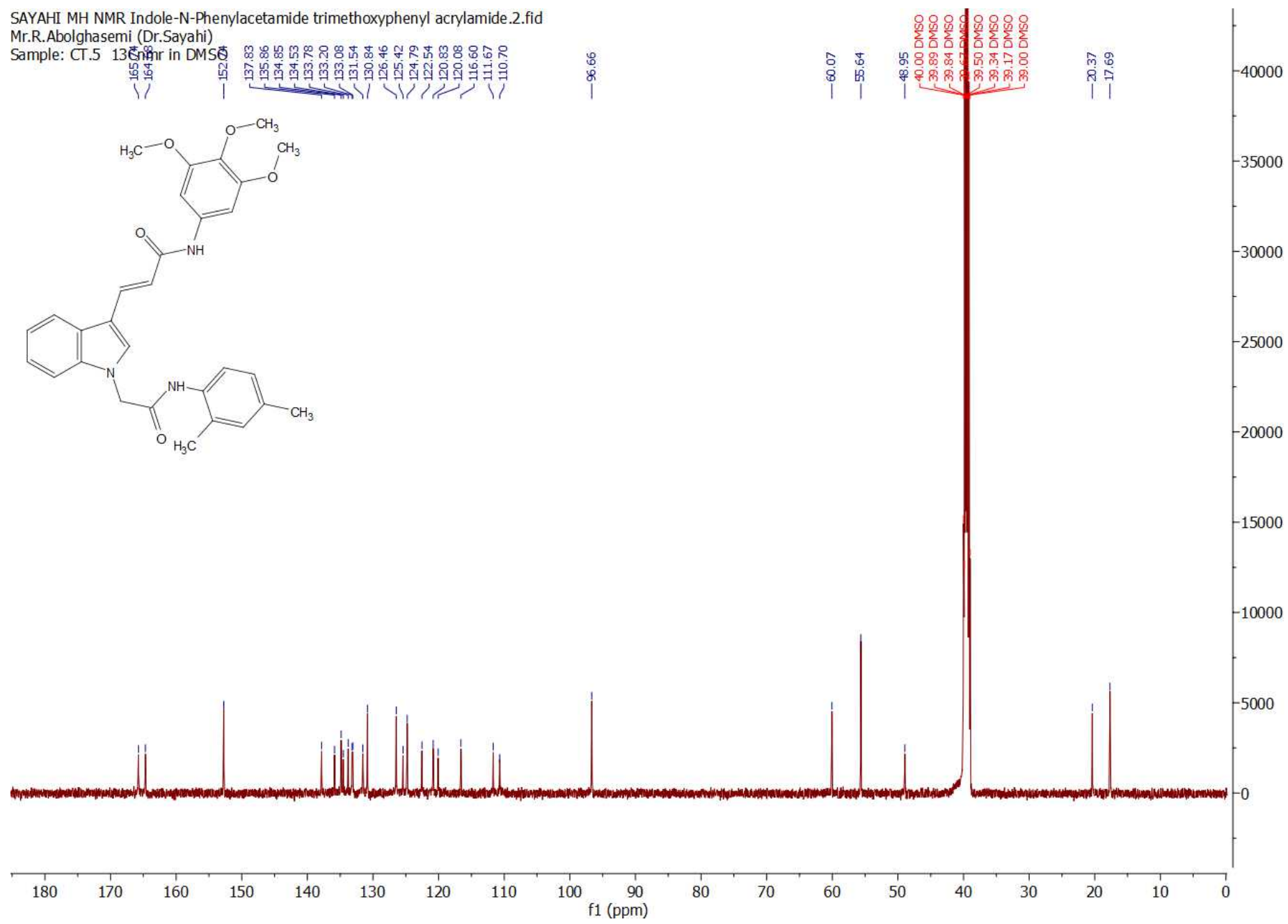

*Figure S18. IR Spectral Data (ATR-FTIR) of **10e***

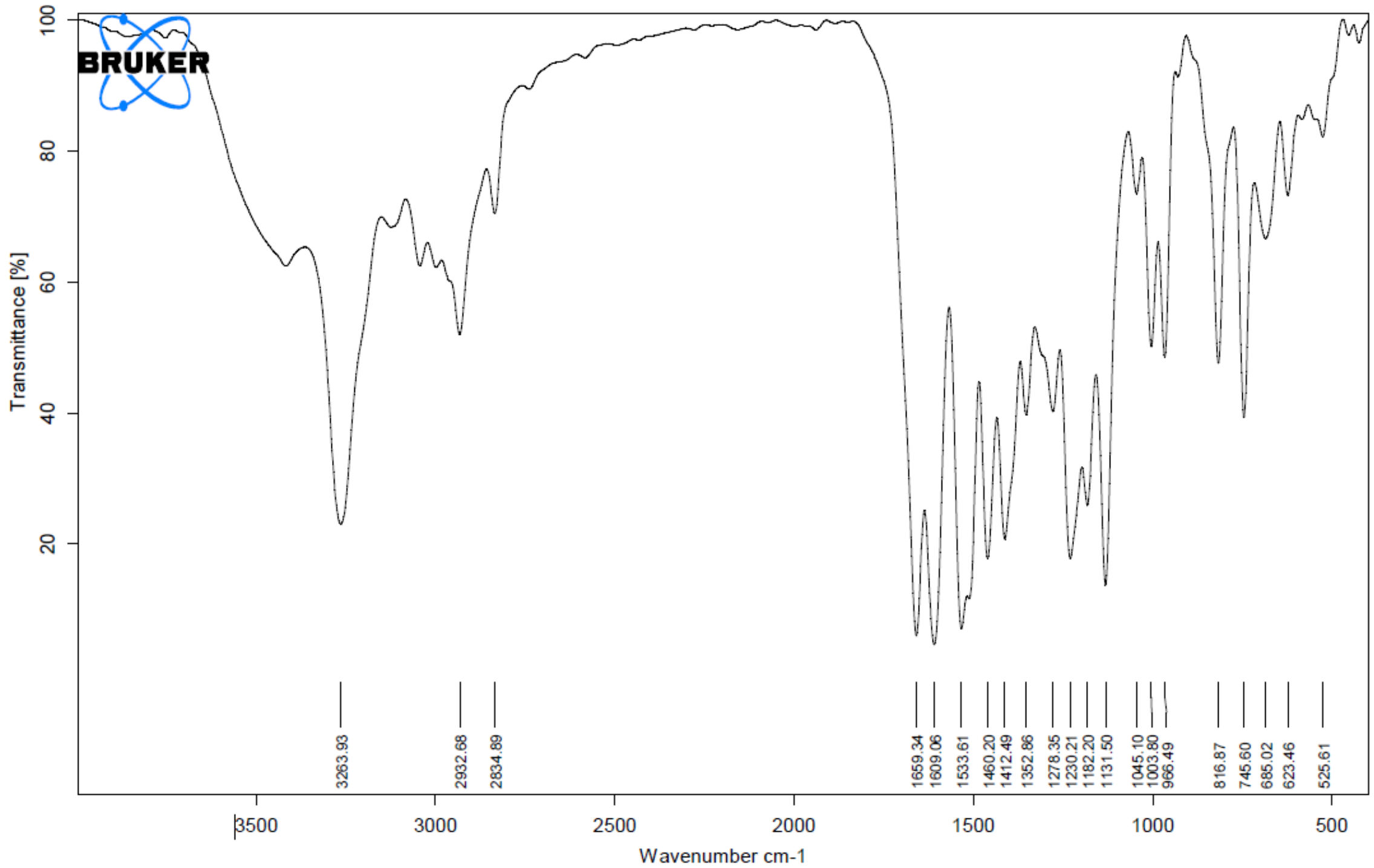

*Figure S1. [1] H NMR (500 MHz, DMSO-d_6) spectrum of **10f***

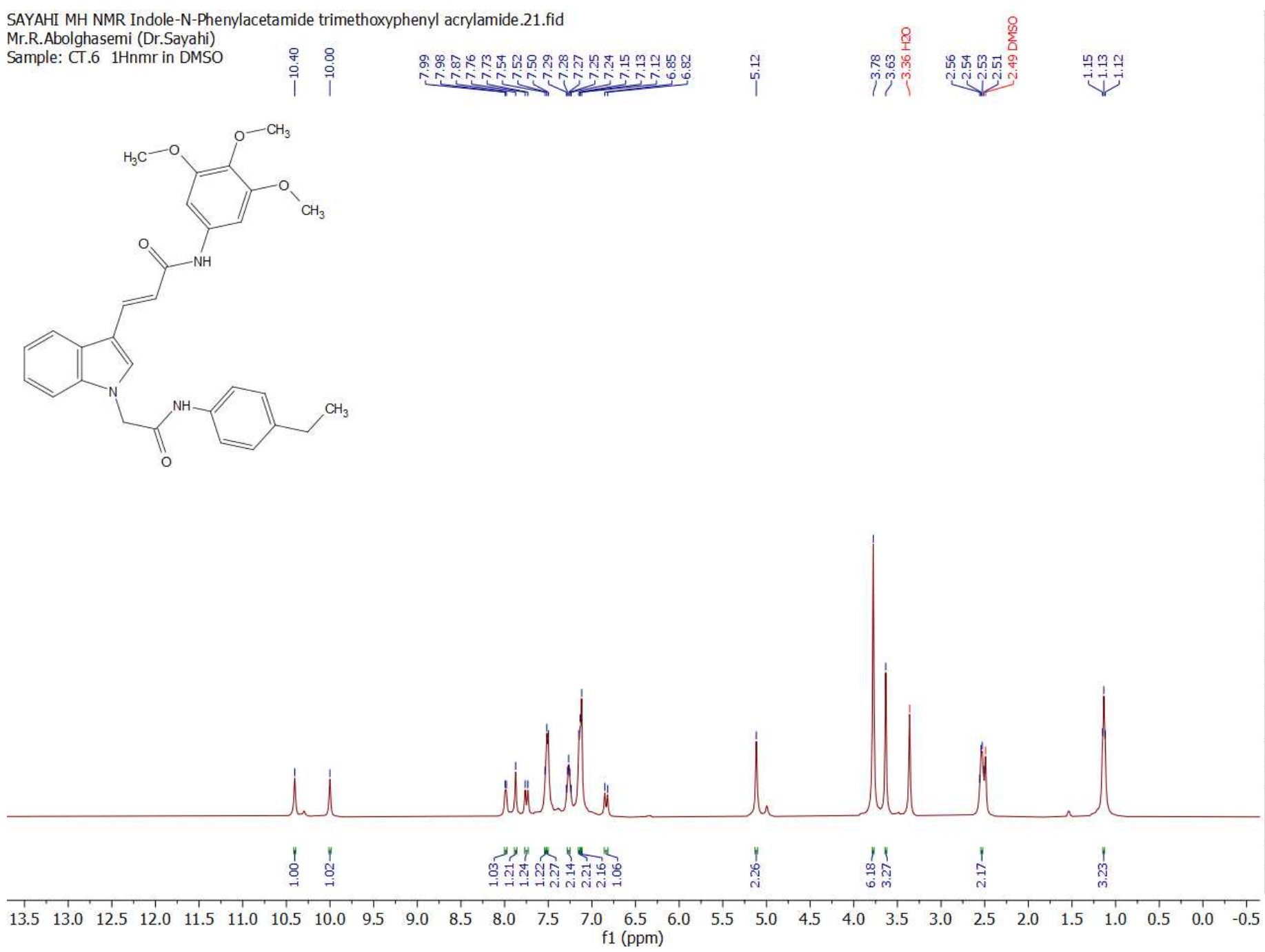

*Figure S20. ^{13}C{^{1}H} NMR (126 MHz, DMSO-d_6) spectrum of **10f***

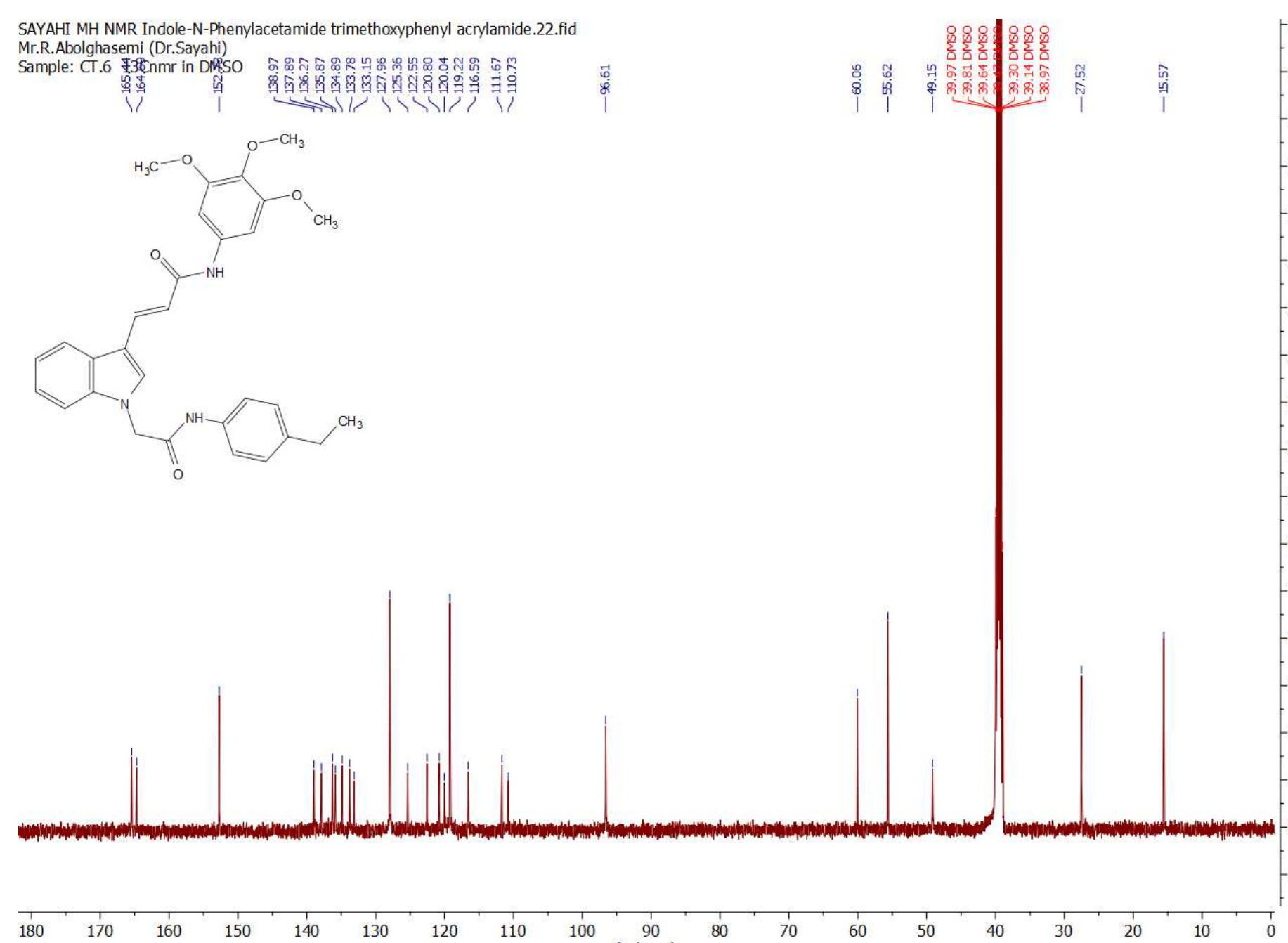

Figure S21. IR spectrum of ***10f***

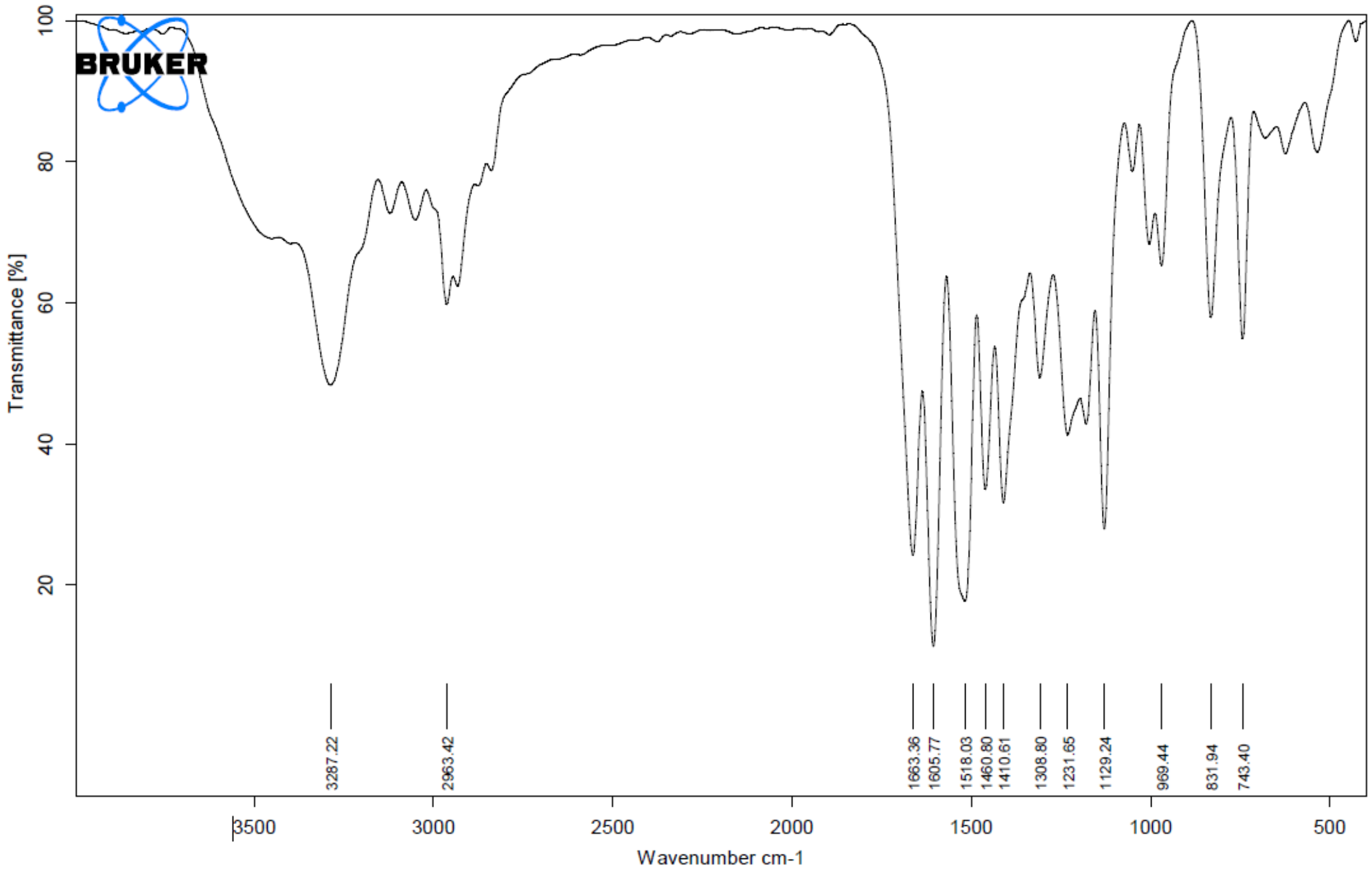

Figure S22. ^{1}H *NMR (500 MHz, DMSO-d_6) spectrum of* ***10g***

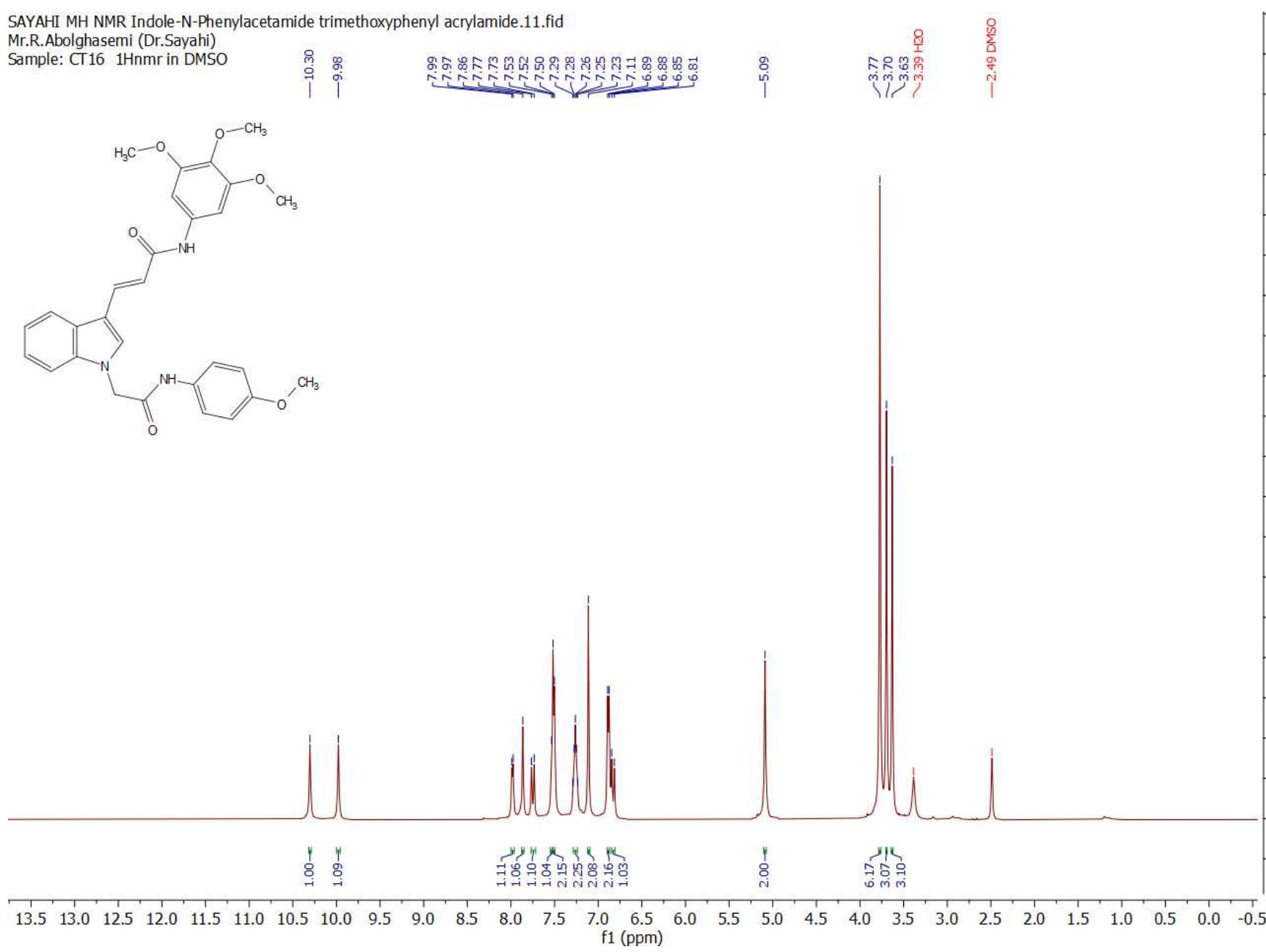

Figure S23. $^{13}C\{^{1}H\}$ *NMR (126 MHz, DMSO-d_6) spectrum of* ***10g***

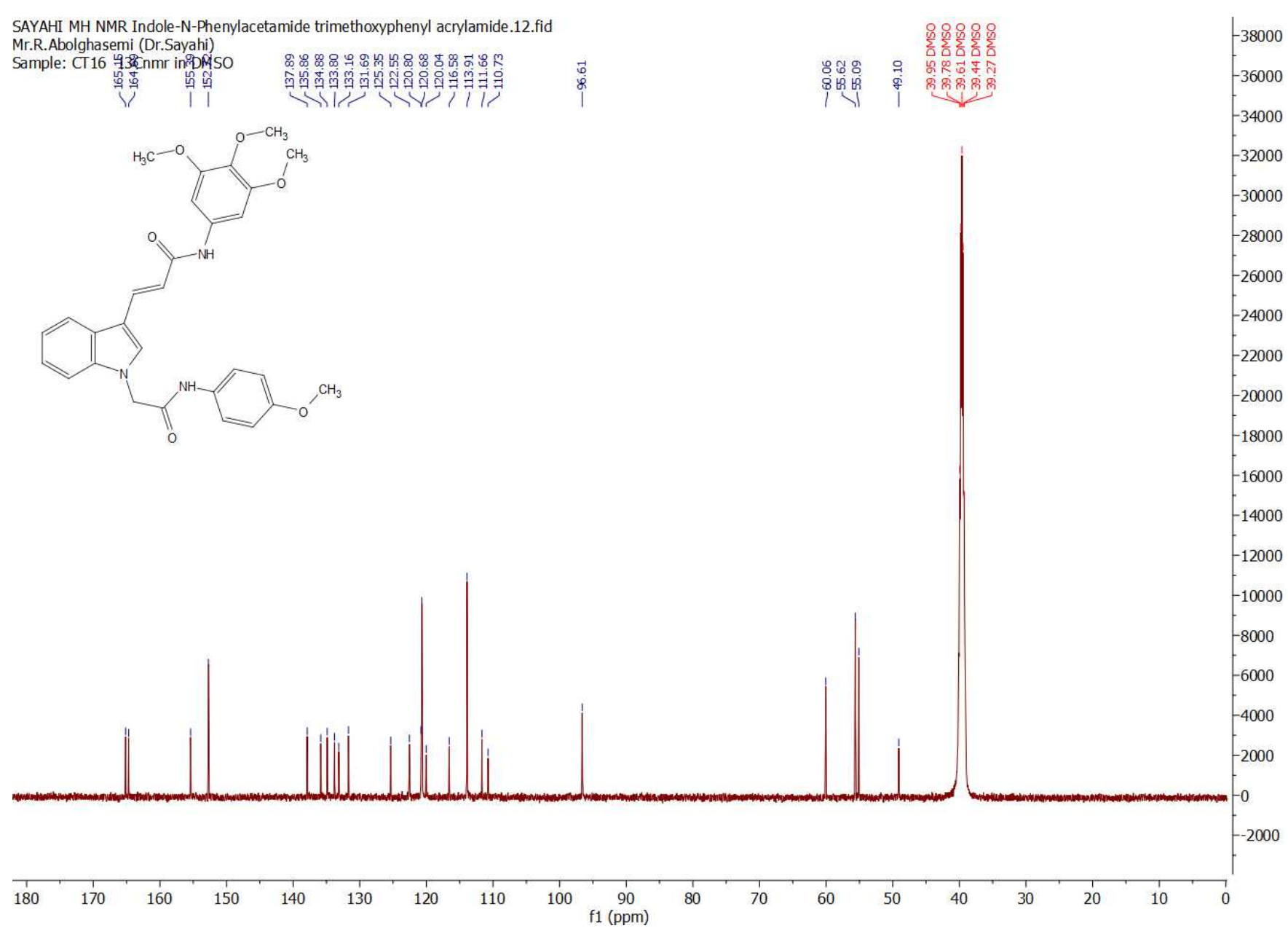

*Figure S24. IR Spectral Data (ATR-FTIR) of **10g***

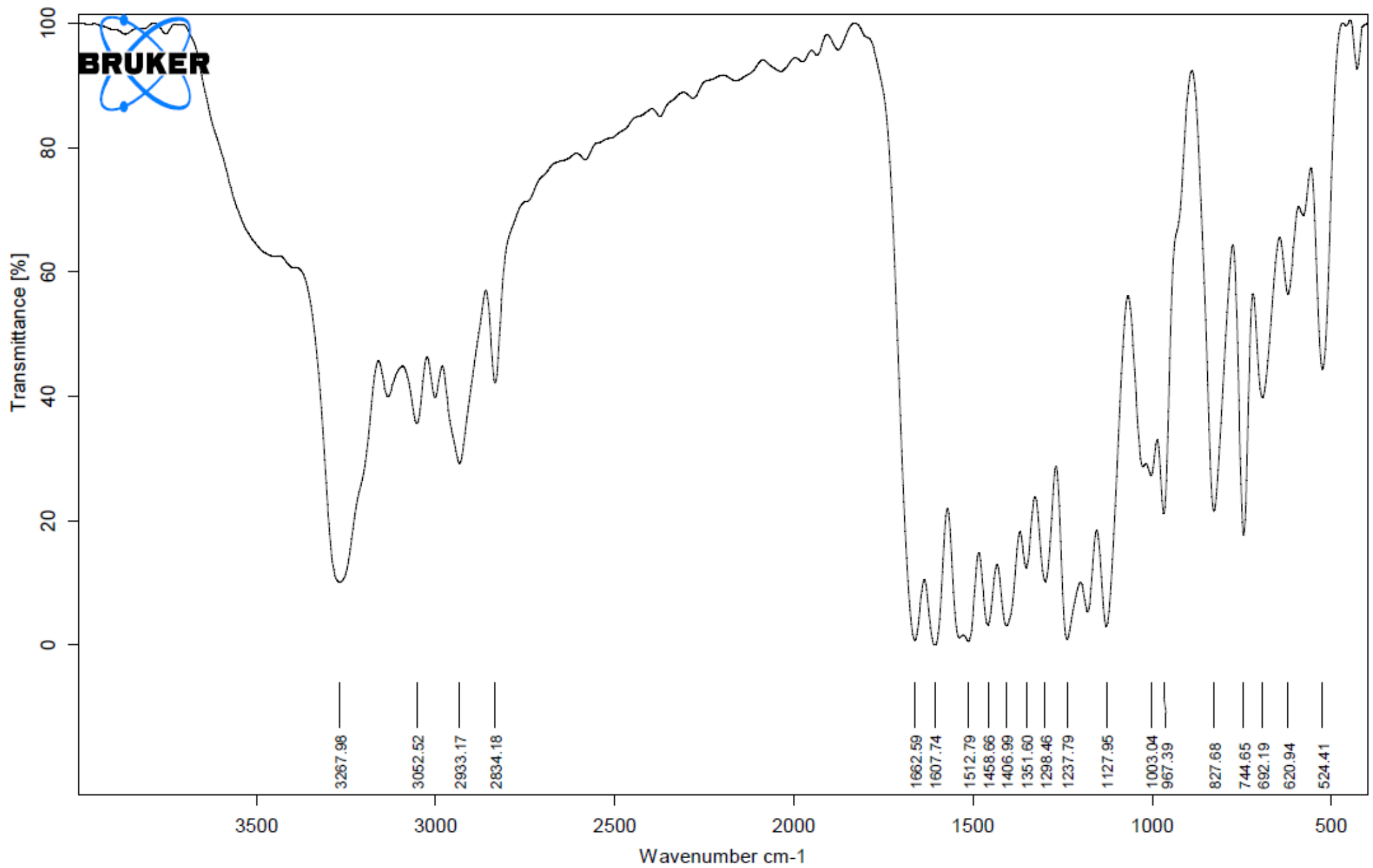

Figure S2. ¹H NMR (500 MHz, DMSO-d_6) spectrum of **10h**

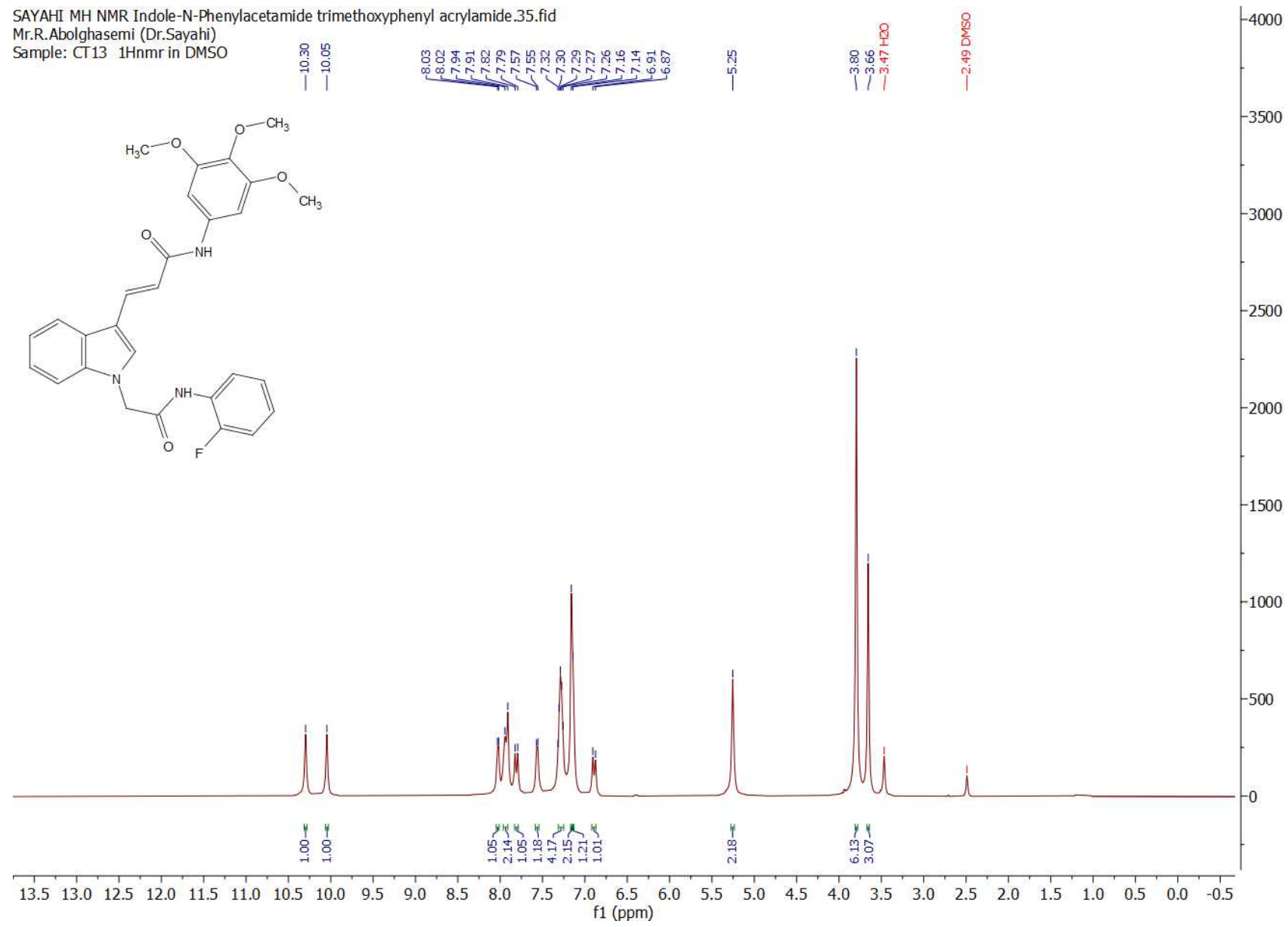

Figure S3. ¹³C{¹H} NMR (126 MHz, DMSO-d_6) spectrum of **10h**

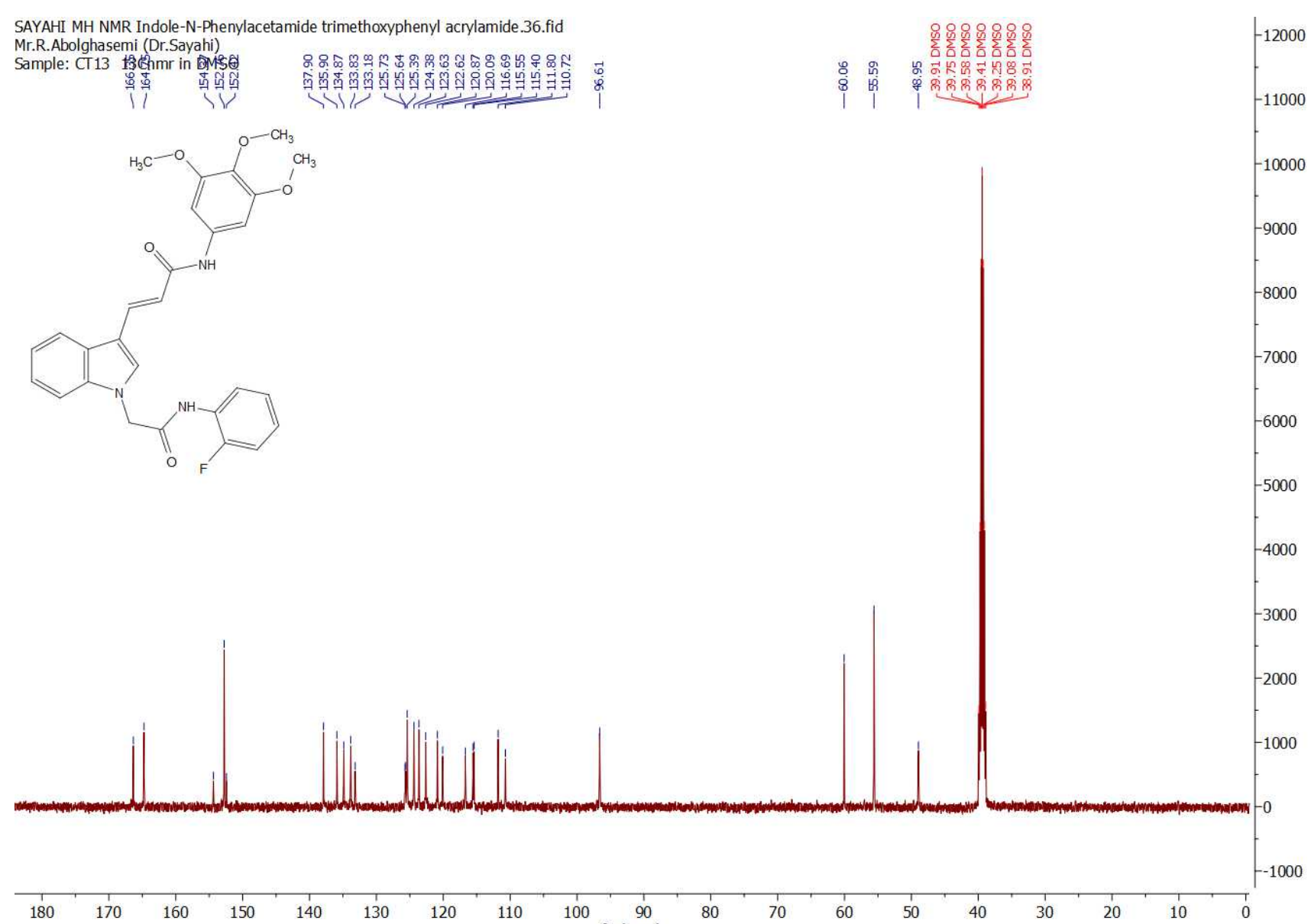

*Figure S27. IR Spectral Data (ATR-FTIR) of **10h***

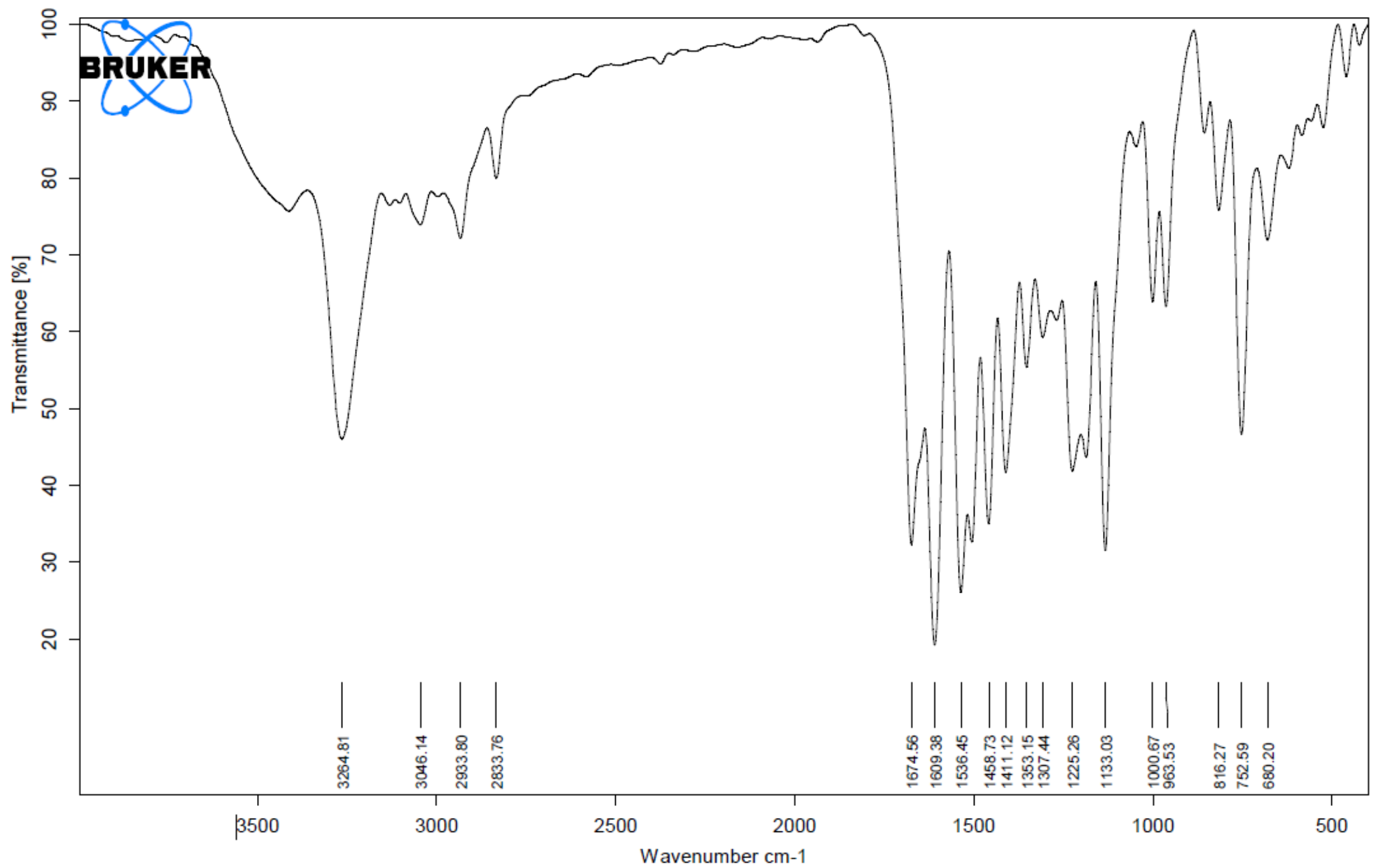

*Figure S28. HPLC Purity Analysis of **10h***

Empower 3 SOFTWARE

CT - Related

SAMPLE INFORMATION

Sample Name:	CT13	Acquired By:	System
Sample Type:	Control	Sample Set Name:	CT
Vial:	14	Acq. Method Set:	CT
Injection #:	1	Processing Method:	1
Injection Volume:	20.00 ul	Channel Name:	W2489 ChA
Run Time:	37.0 Minutes	Proc. Chnl. Descr.:	W2489 ChA 230nm
Date Acquired:	1/18/2026 9:17:53 PM GST		
Date Processed:	6/21/2026 1:51:28 PM GST		

Auto-Scaled Chromatogram

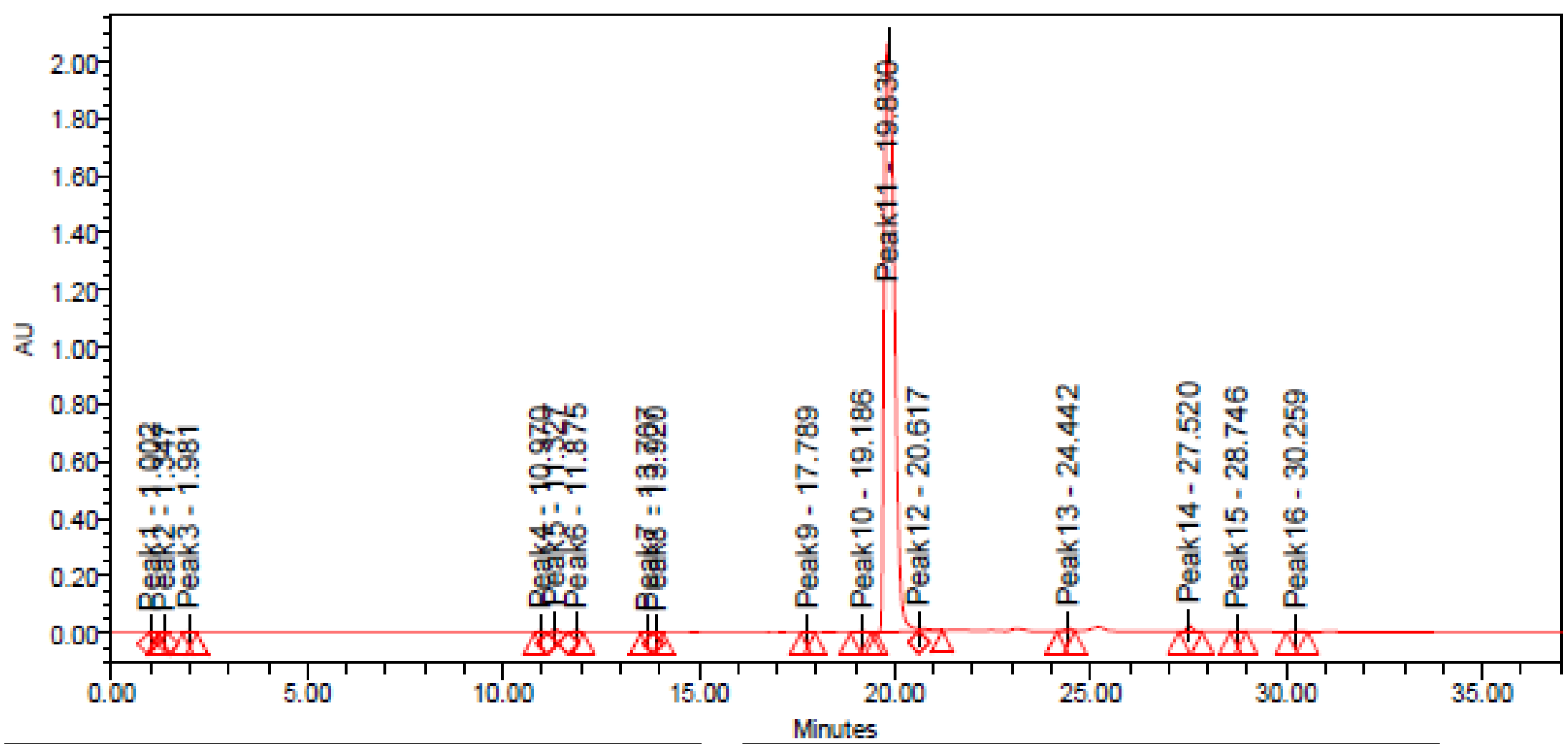

	Peak Name	Retention Time (min)	Height	Area	% Area
1	Peak1	1.002	3121	24207	0.07
2	Peak2	1.347	2992	17903	0.05
3	Peak3	1.981	4696	35735	0.10
4	Peak4	10.970	5357	46366	0.13
5	Peak5	11.327	13187	121927	0.33
6	Peak6	11.875	8106	72907	0.20
7	Peak7	13.707	2215	22042	0.06
8	Peak8	13.920	1050	9493	0.03
9	Peak9	17.789	1966	17909	0.05
10	Peak10	19.186	3349	35683	0.10
11	Peak11	19.830	2060799	36105448	97.45
12	Peak12	20.617	8751	151919	0.41
13	Peak13	24.442	9662	113064	0.31
14	Peak14	27.520	22023	228484	0.62
15	Peak15	28.746	2417	24513	0.07
16	Peak16	30.259	1626	21416	0.06

Reported by User: System
Report Method: Chemi
Report Method ID: 1 11636
Page: 1 of 1

Project Name: CT
Date Printed:
6/21/2026
1:55:37 PM Asia/Dubai

*Figure S29. HRMS Spectra of **10h***

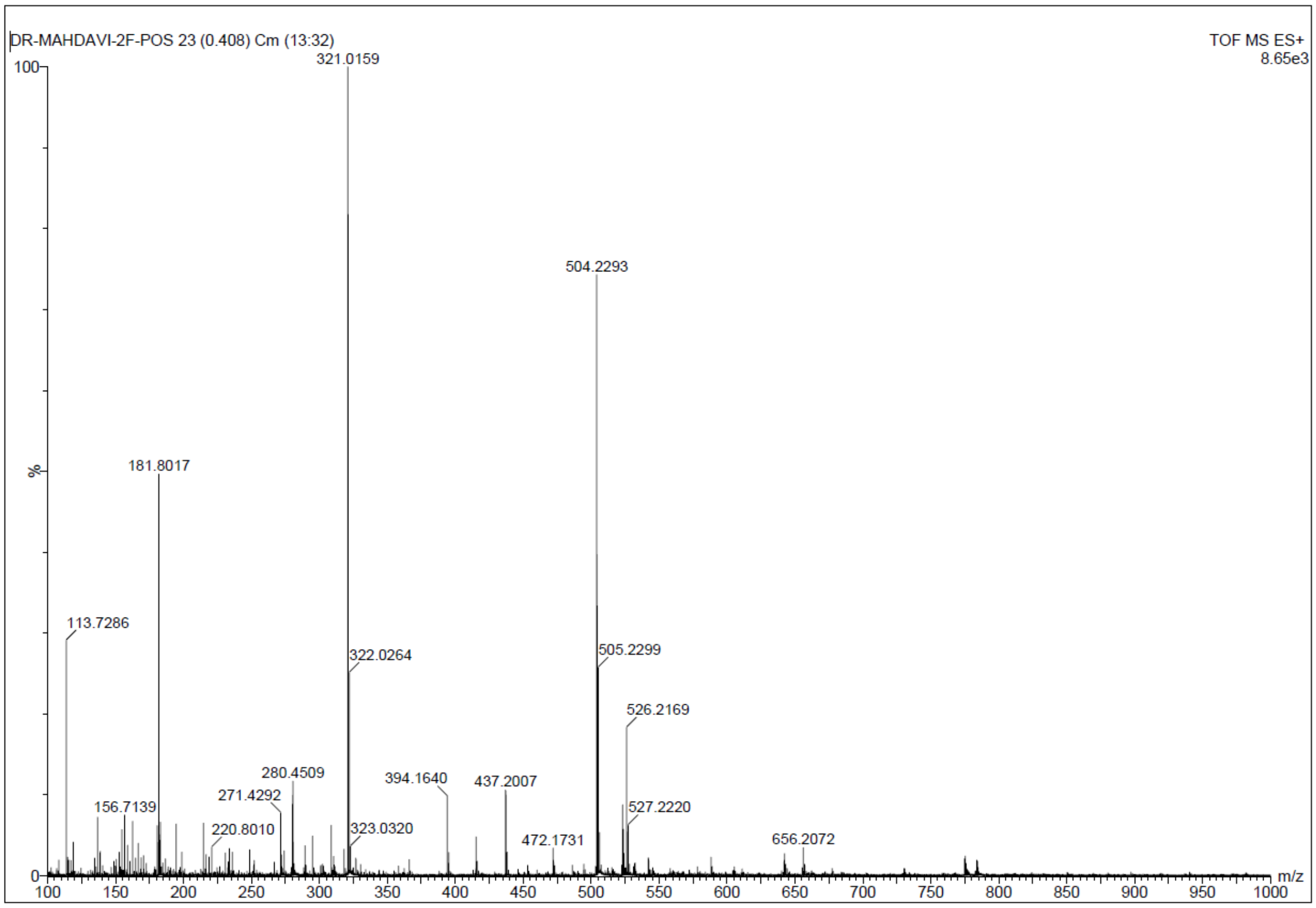

Figure S30. ^{1}H NMR (500 MHz, DMSO-d_6) spectrum of ***10i***

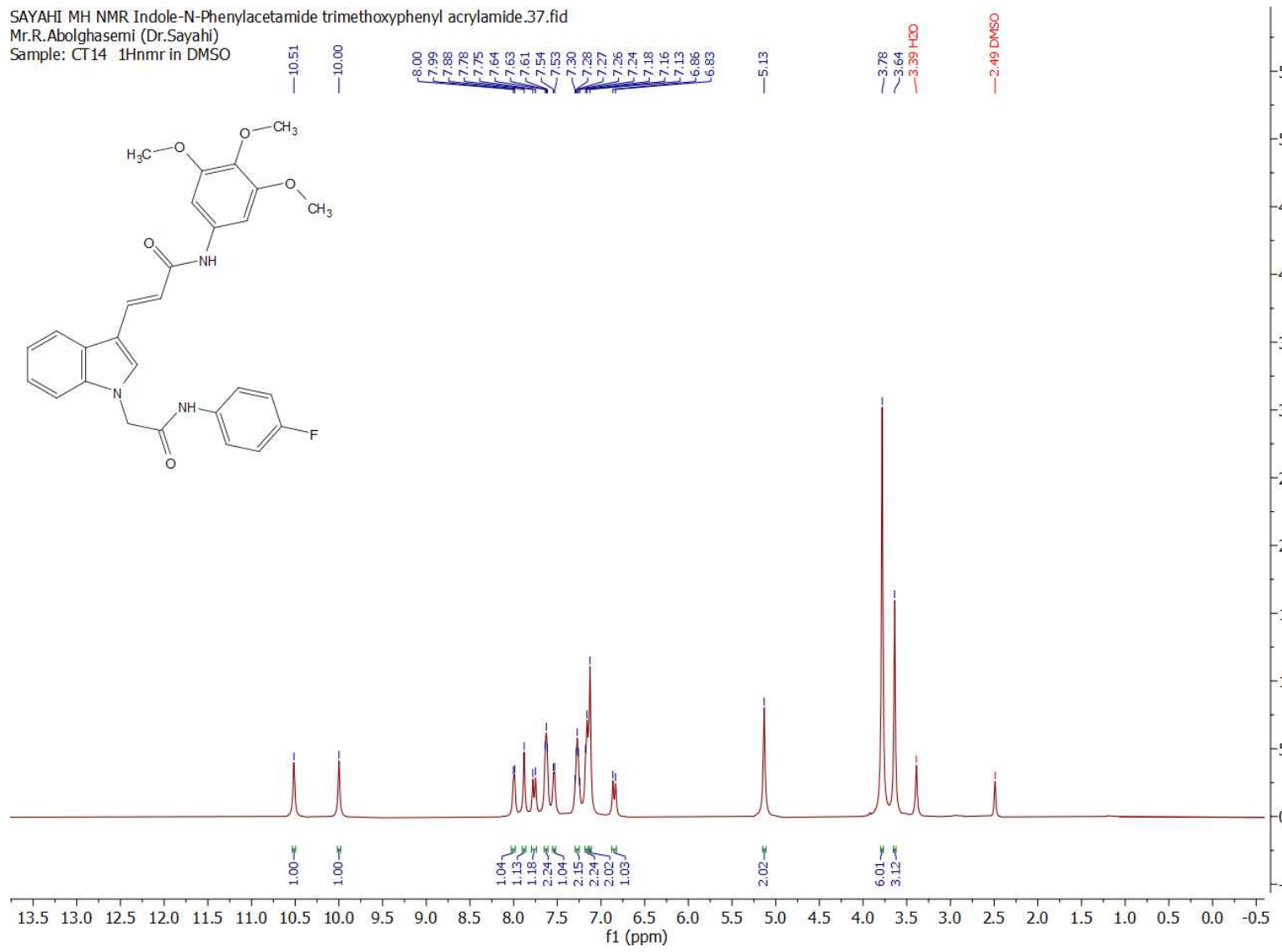

Figure S31. ^{13}C{^{1}H} NMR (126 MHz, DMSO-d_6) spectrum of ***10i***

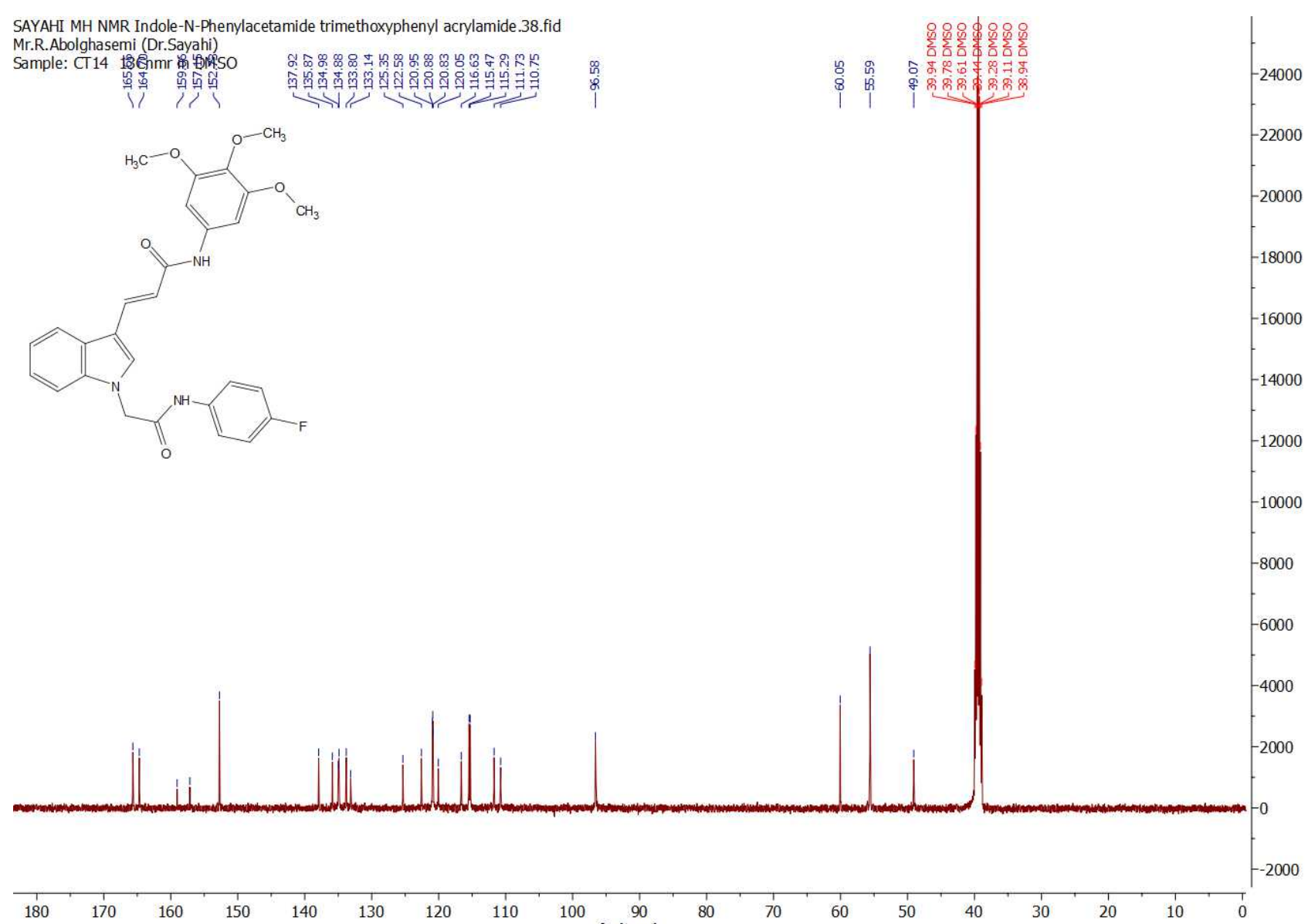

*Figure S32. IR Spectral Data (ATR-FTIR) of **10i***

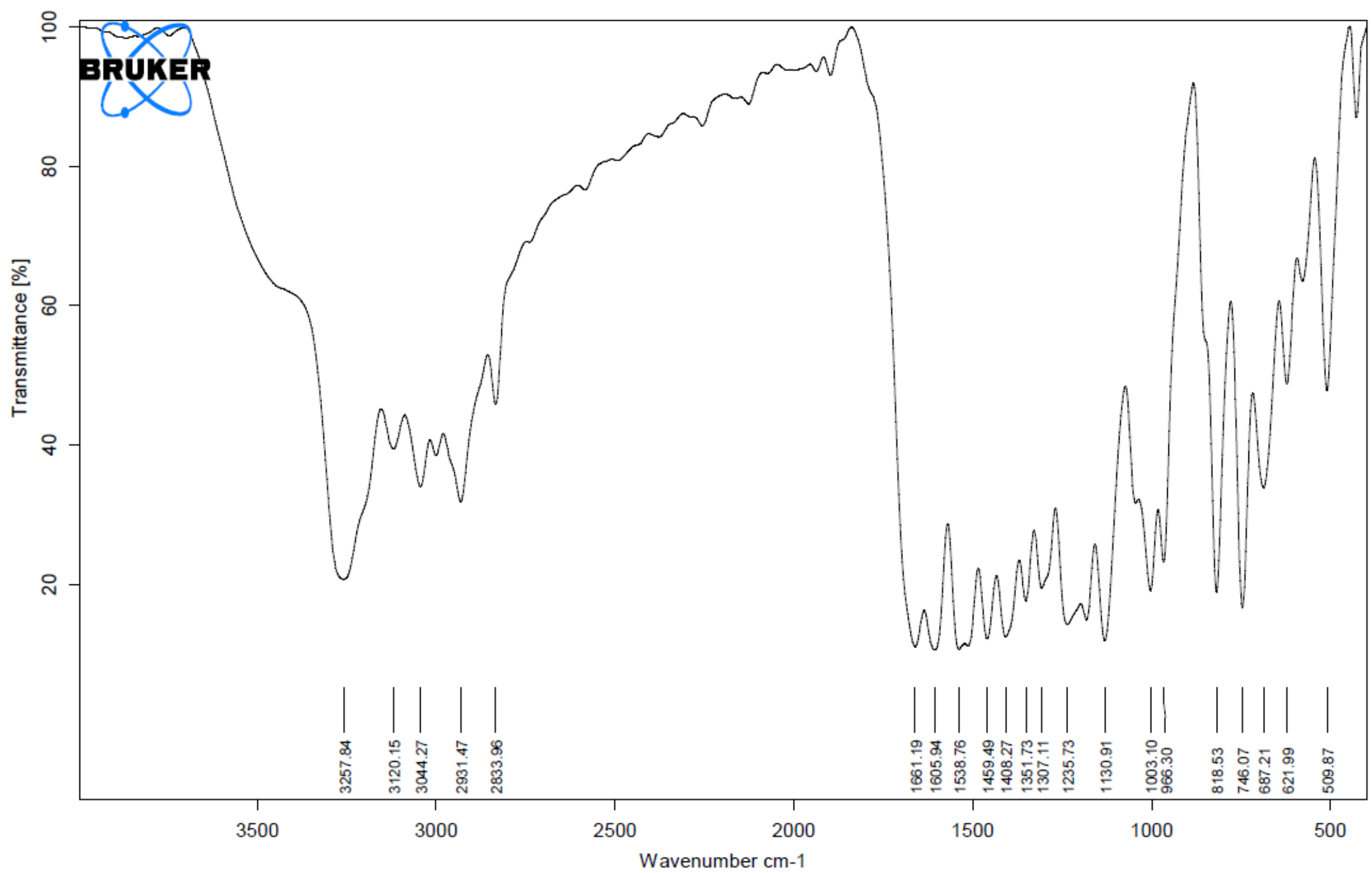

Figure S33. ^{1}H NMR (500 MHz, DMSO-d_6) spectrum of ***10j***

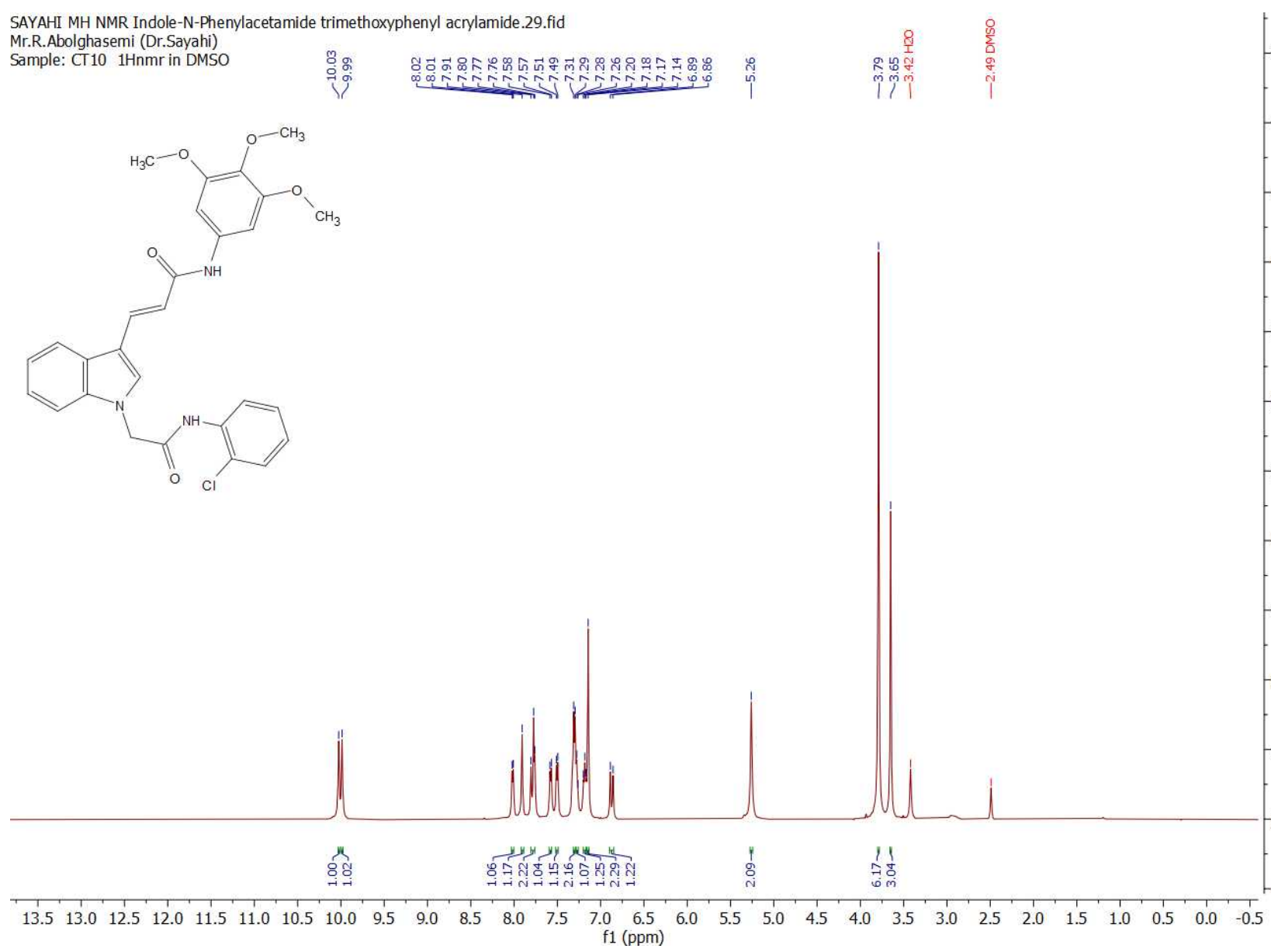

Figure S34. ^{13}C{^{1}H} NMR (126 MHz, DMSO-d_6) spectrum of ***10j***

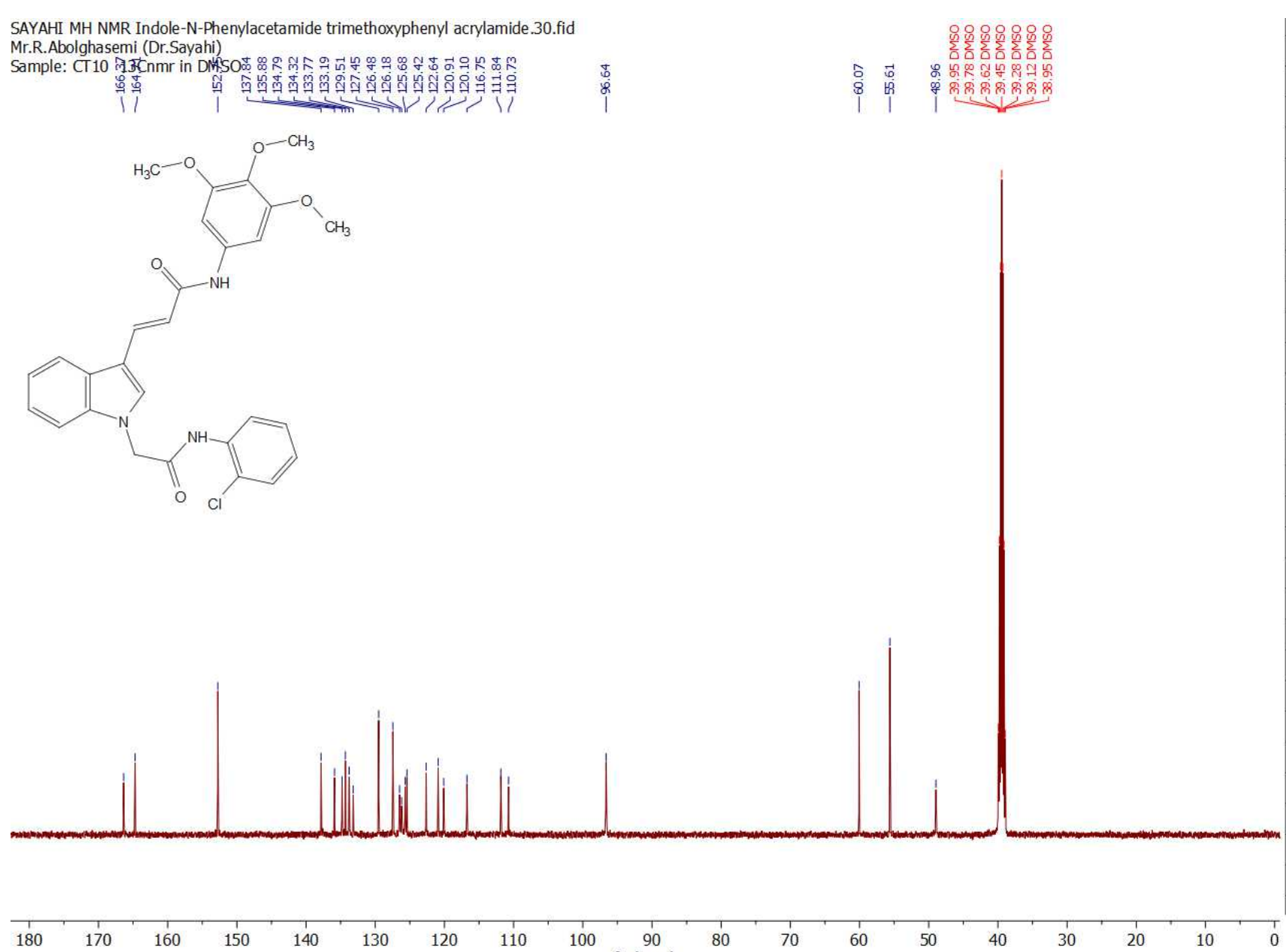

Figure S35. IR Spectral Data (ATR-FTIR) of ***10j***

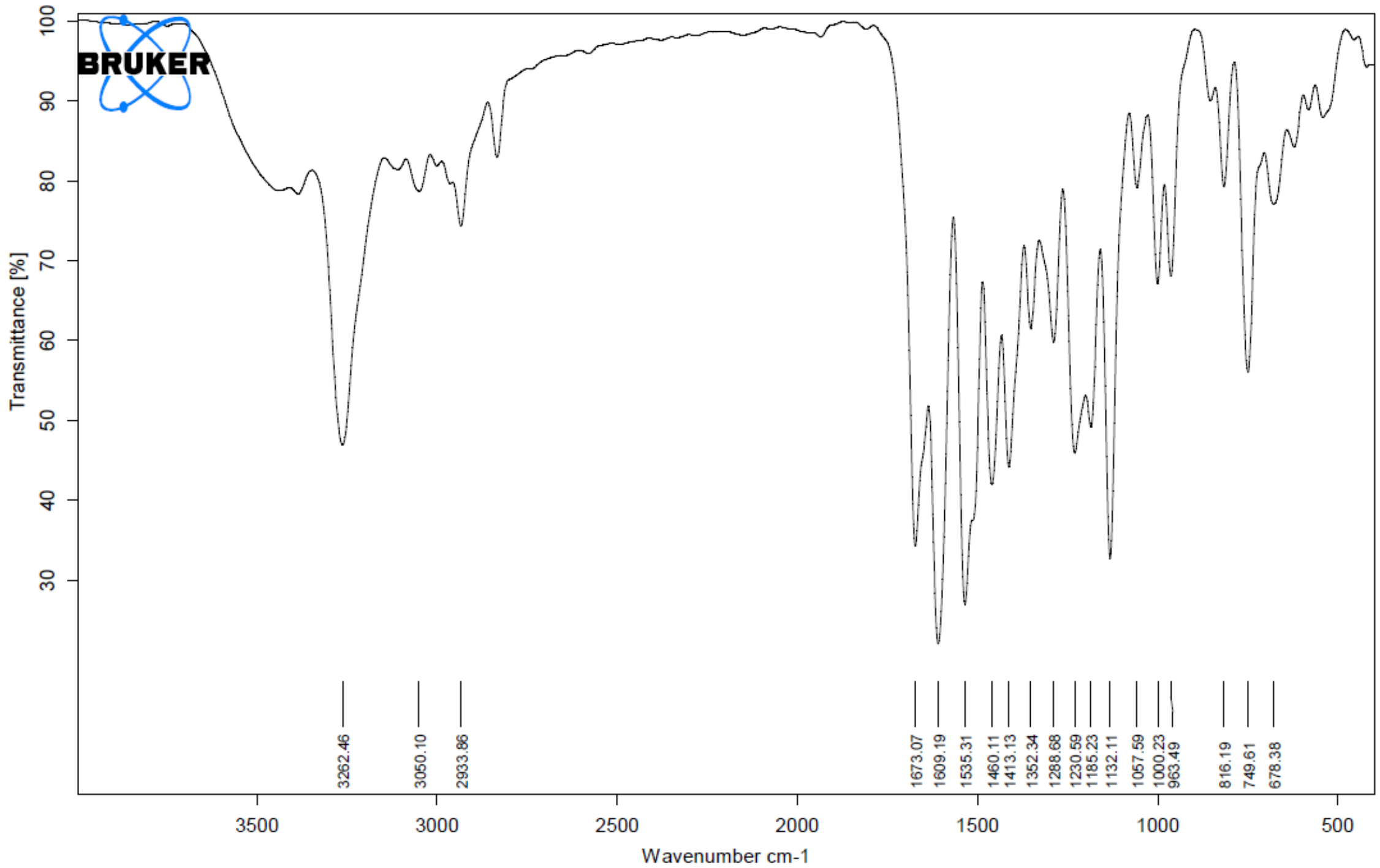

Figure S36. ^{1}H NMR (500 MHz, DMSO-d_6) spectrum of ***10k***

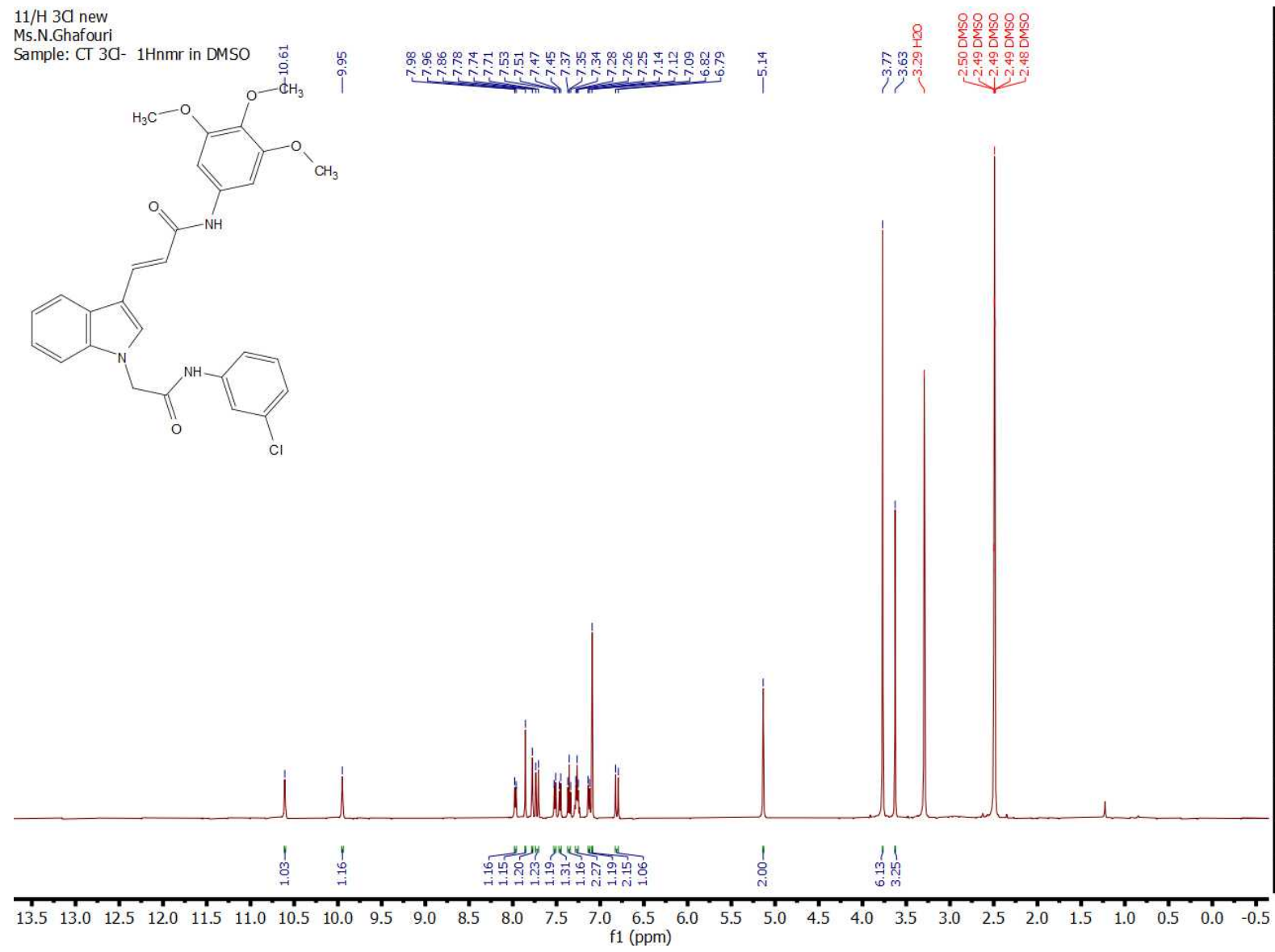

Figure S37. ^{13}C{^{1}H} NMR (126 MHz, DMSO-d_6) spectrum of ***10k***

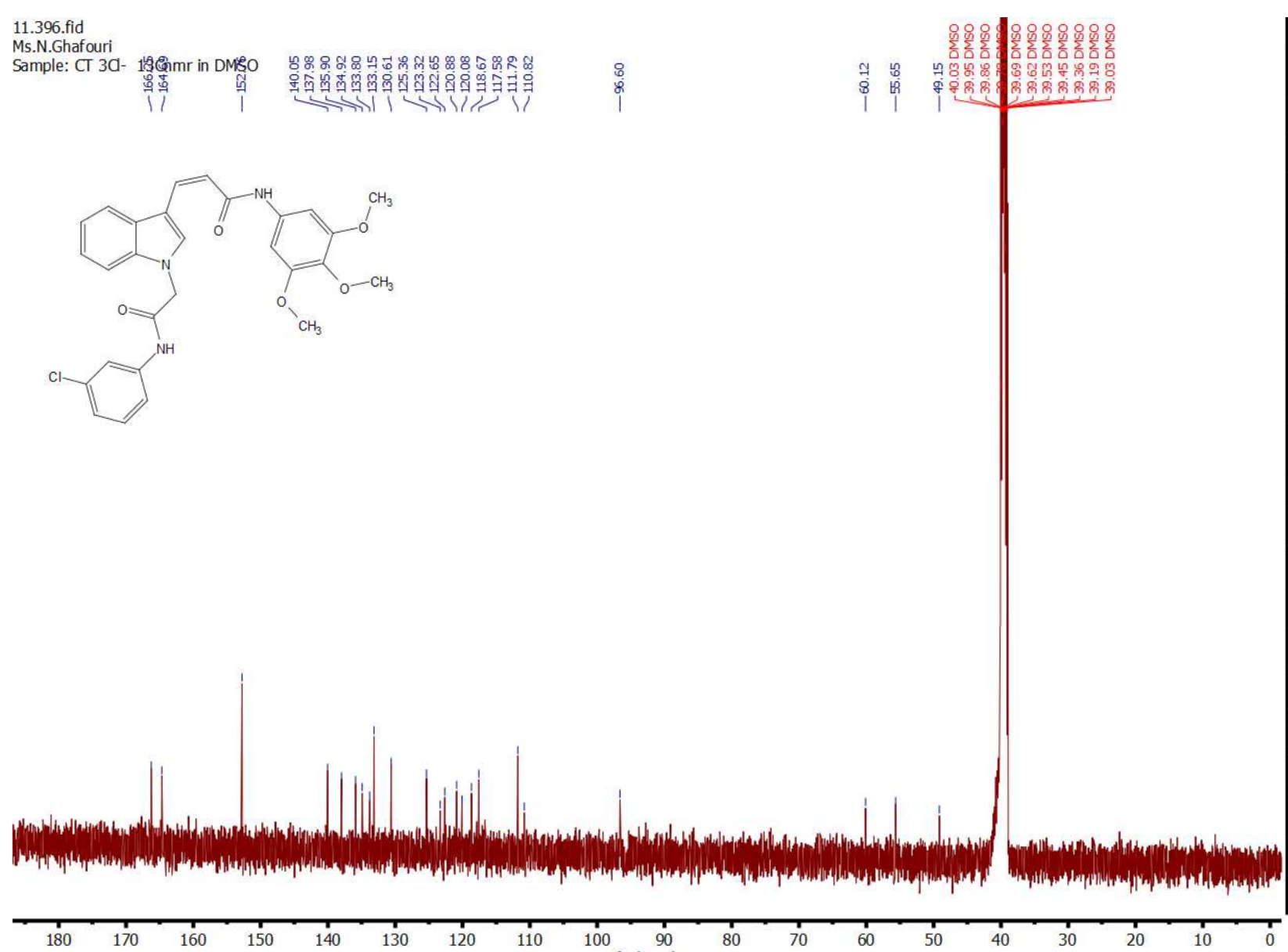

*Figure S38. IR Spectral Data (ATR-FTIR) of **10k***

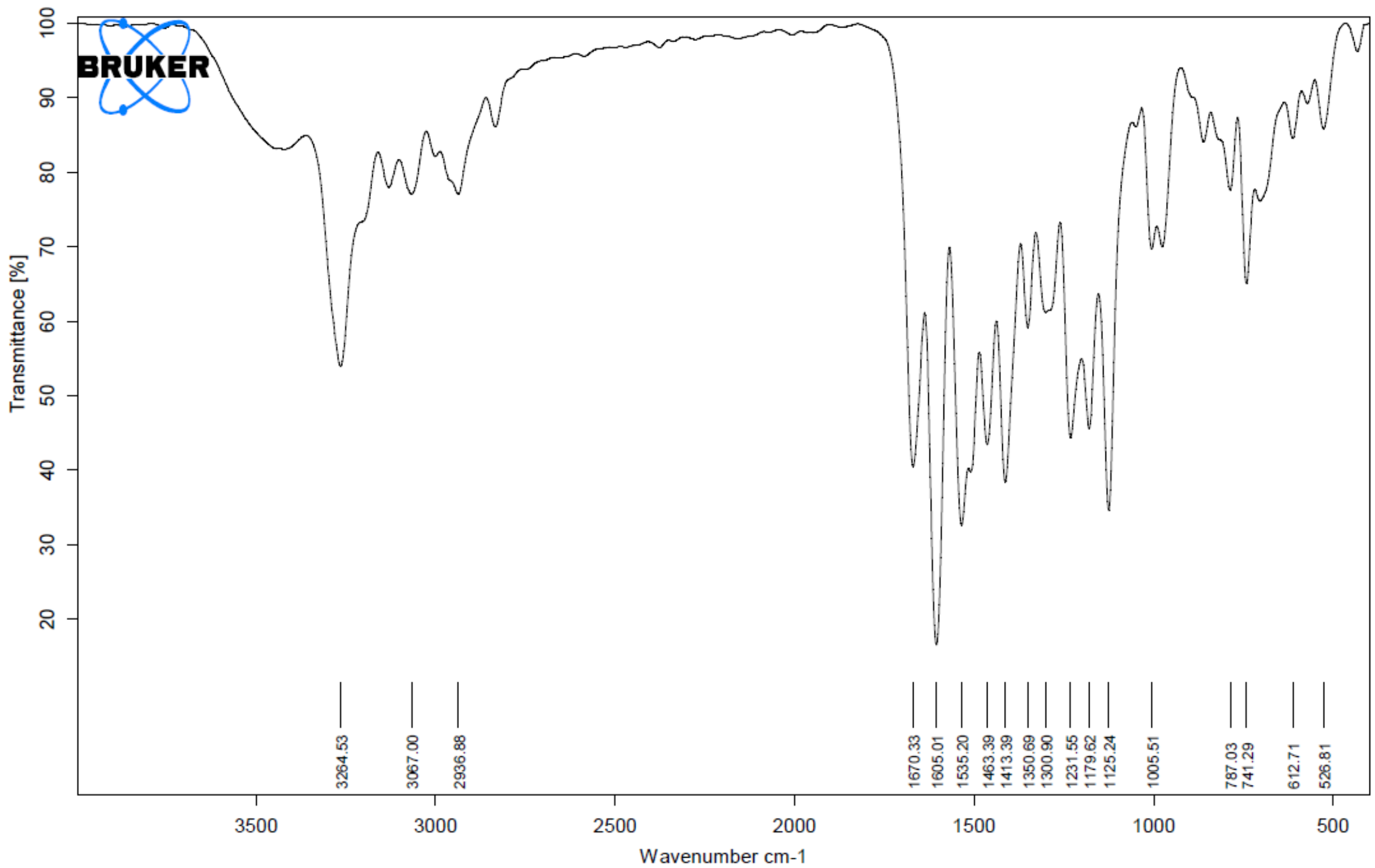

*Figure S39. ^{1}H NMR (500 MHz, DMSO-d_6) spectrum of **10l***

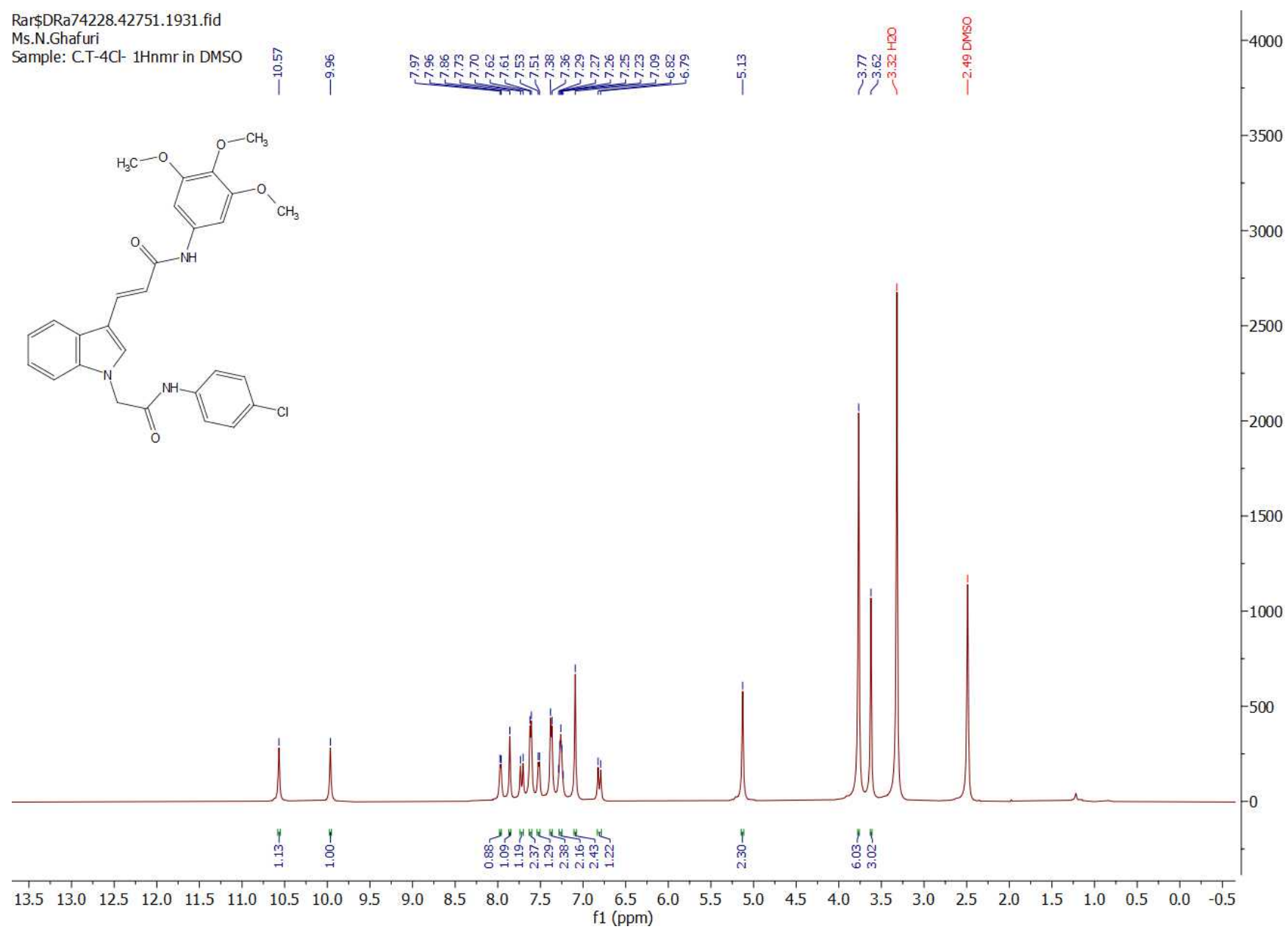

*Figure S40. ^{13}C{^{1}H} NMR (126 MHz, DMSO-d_6) spectrum of **10l***

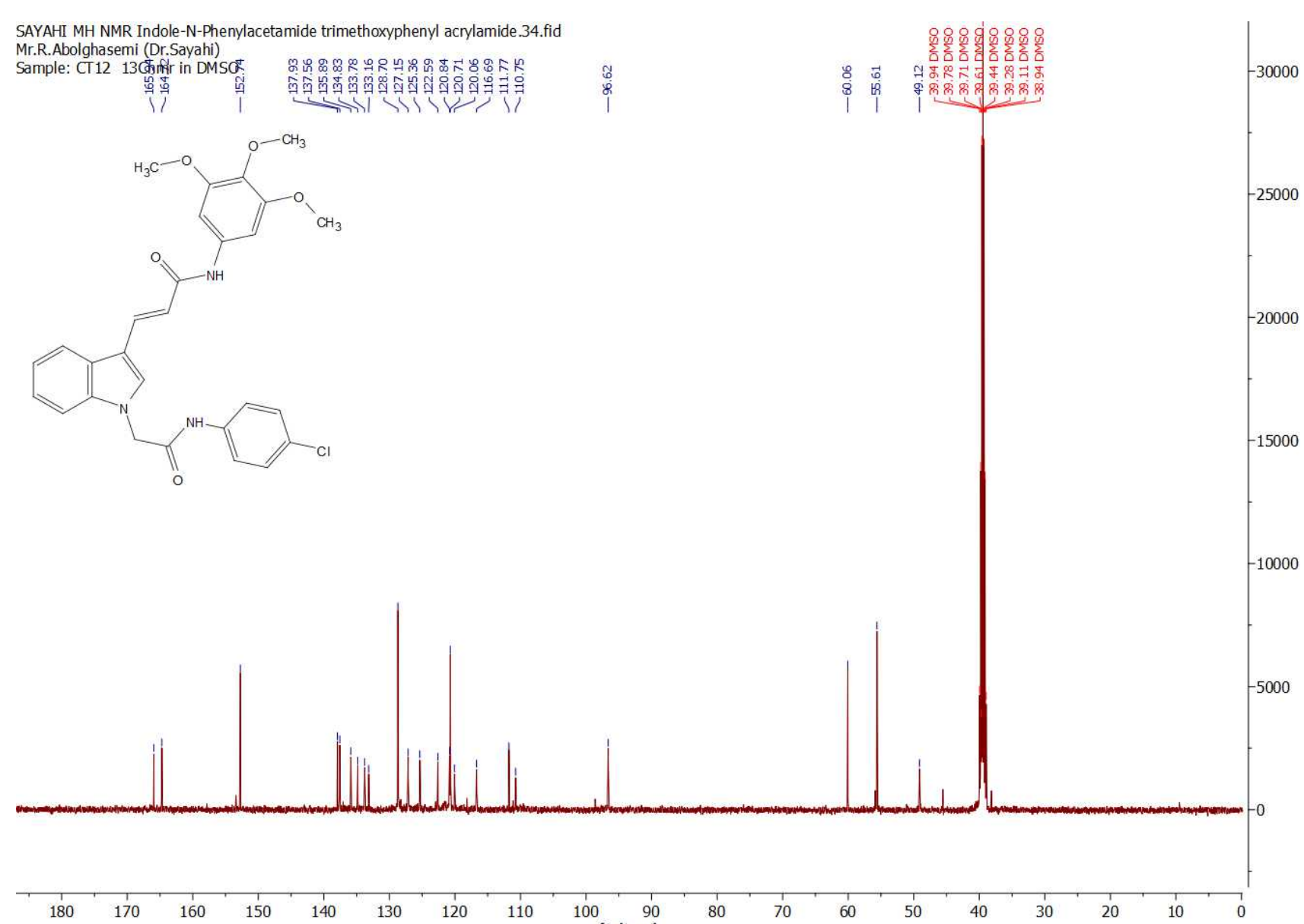

*Figure S41. IR spectrum of **10l***

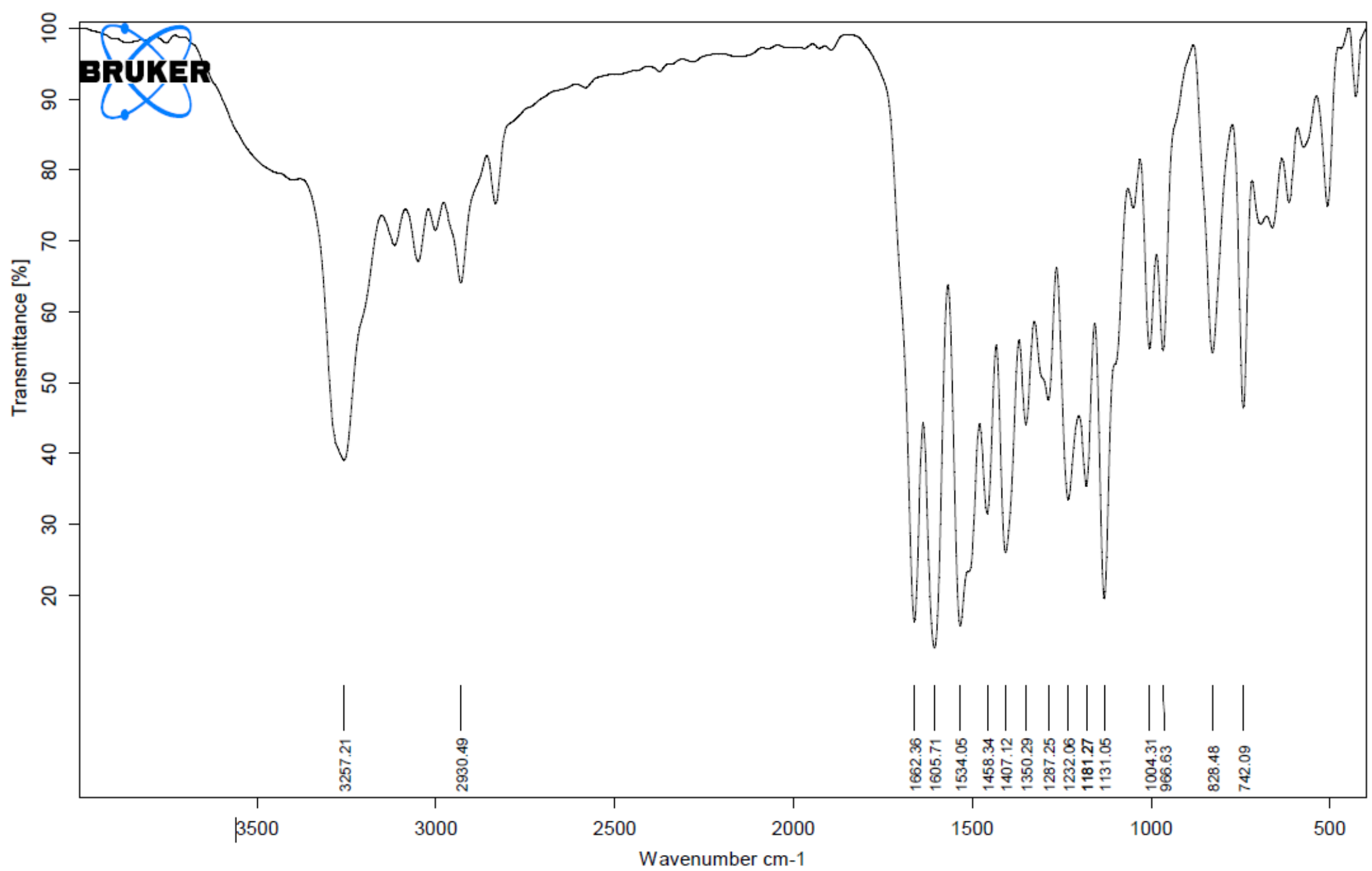

Figure S42. ^1H *NMR (500 MHz, DMSO-d_6) spectrum of* ***10m***

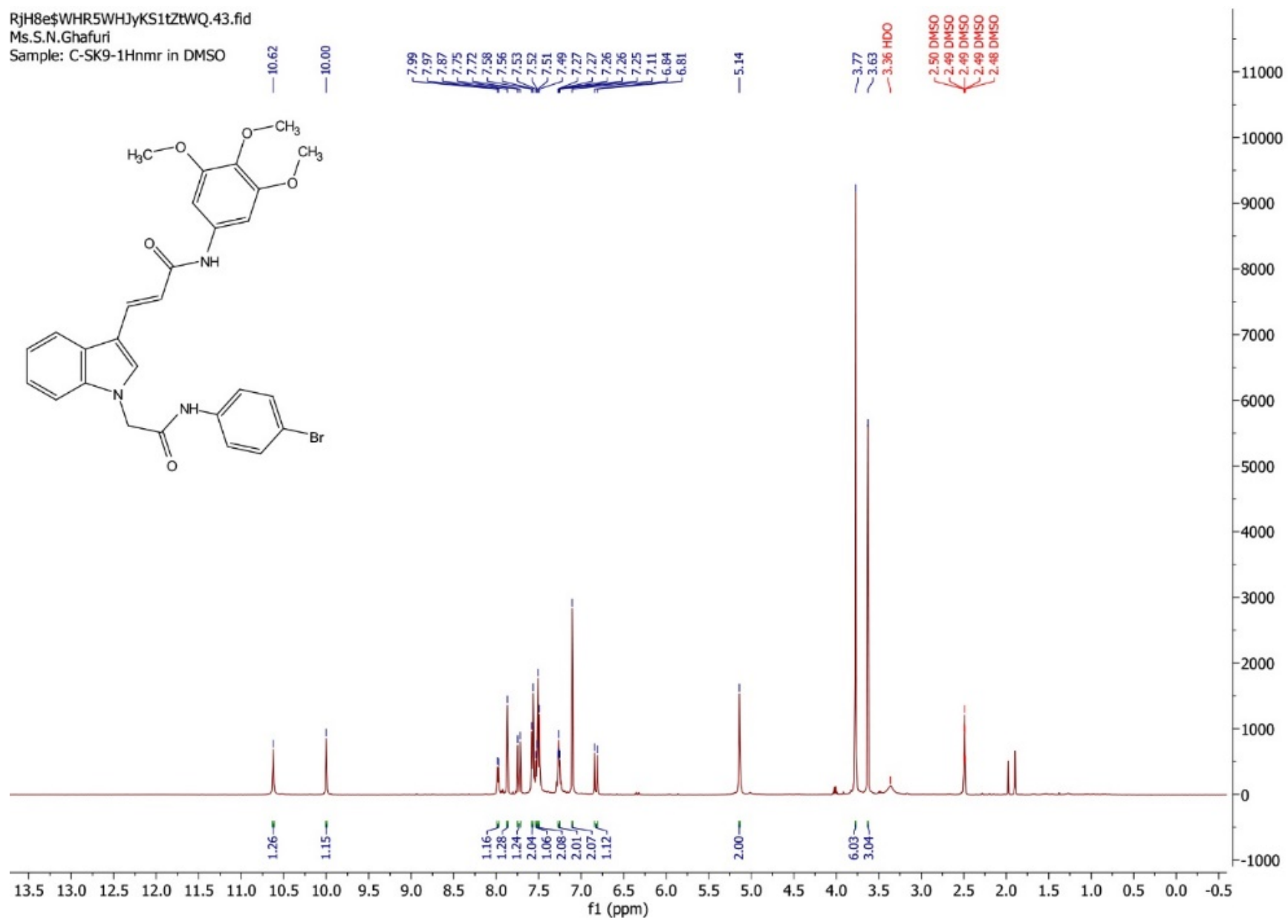

Figure S43. $^{13}C\{^1H\}$ *NMR (126 MHz, DMSO-d_6) spectrum of* ***10m***

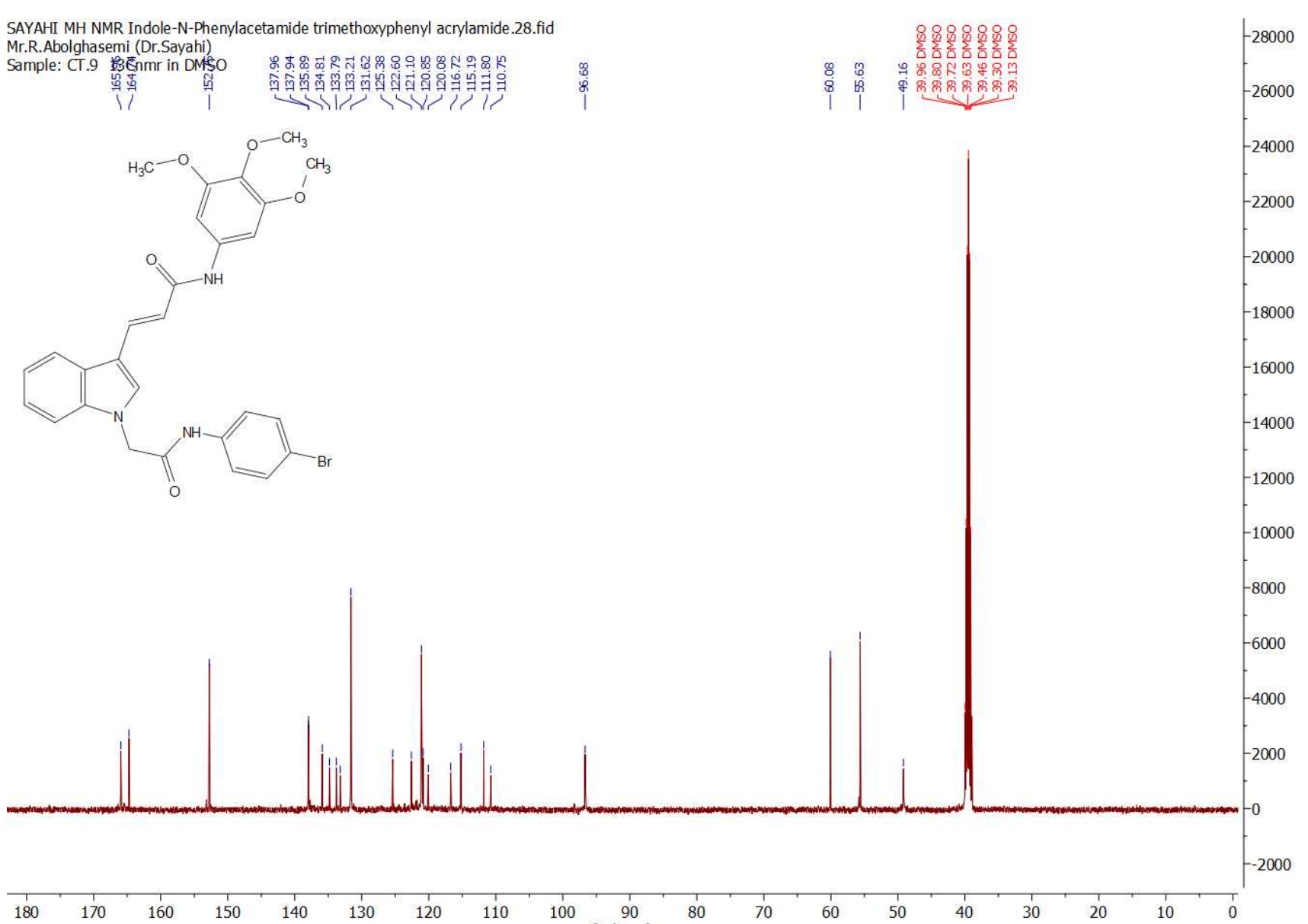

Figure S44. IR spectrum of ***10m***

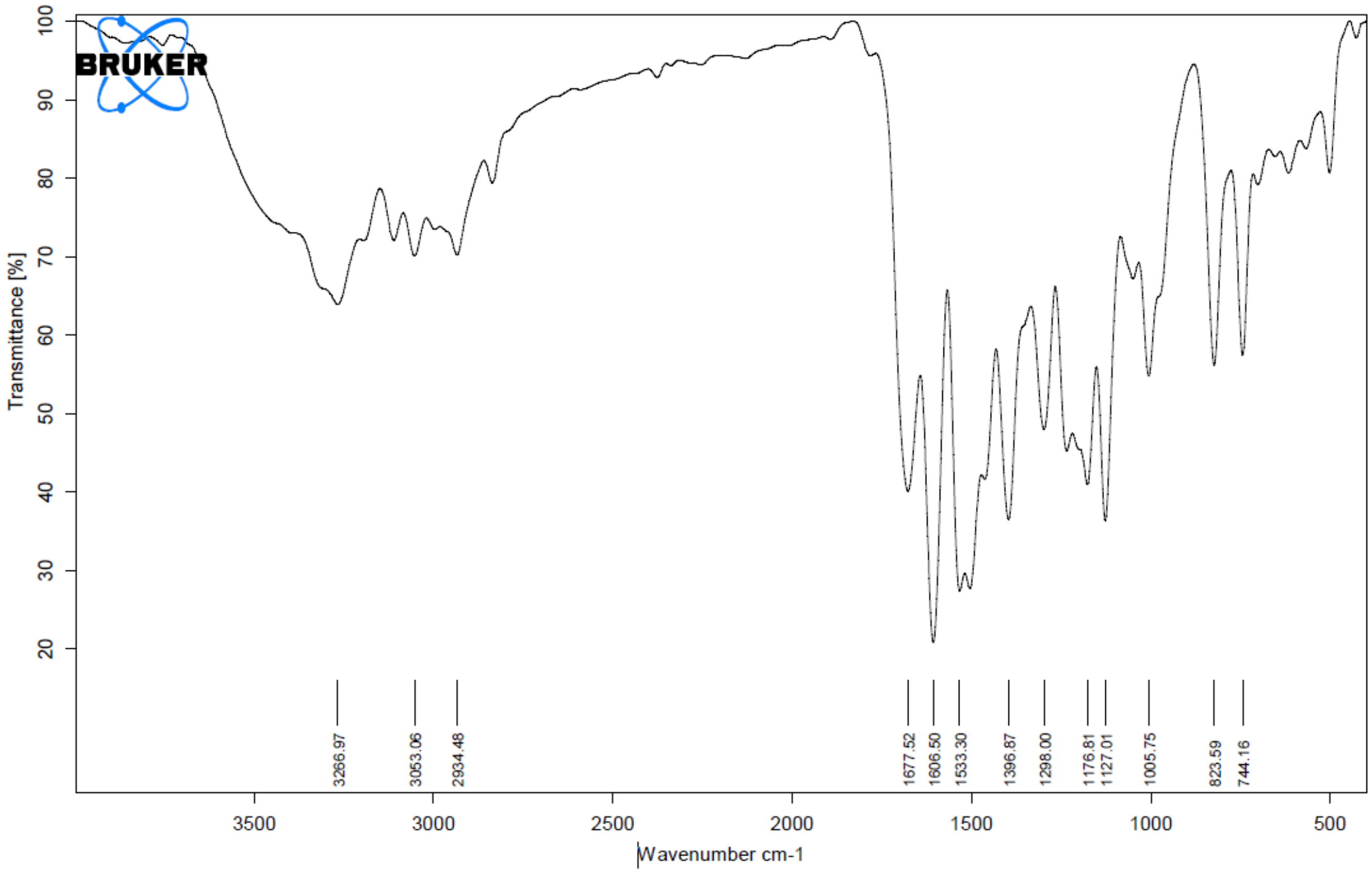

Figure S45. [1]*H NMR (500 MHz, DMSO-d*$_6$*) spectrum of* ***10n***

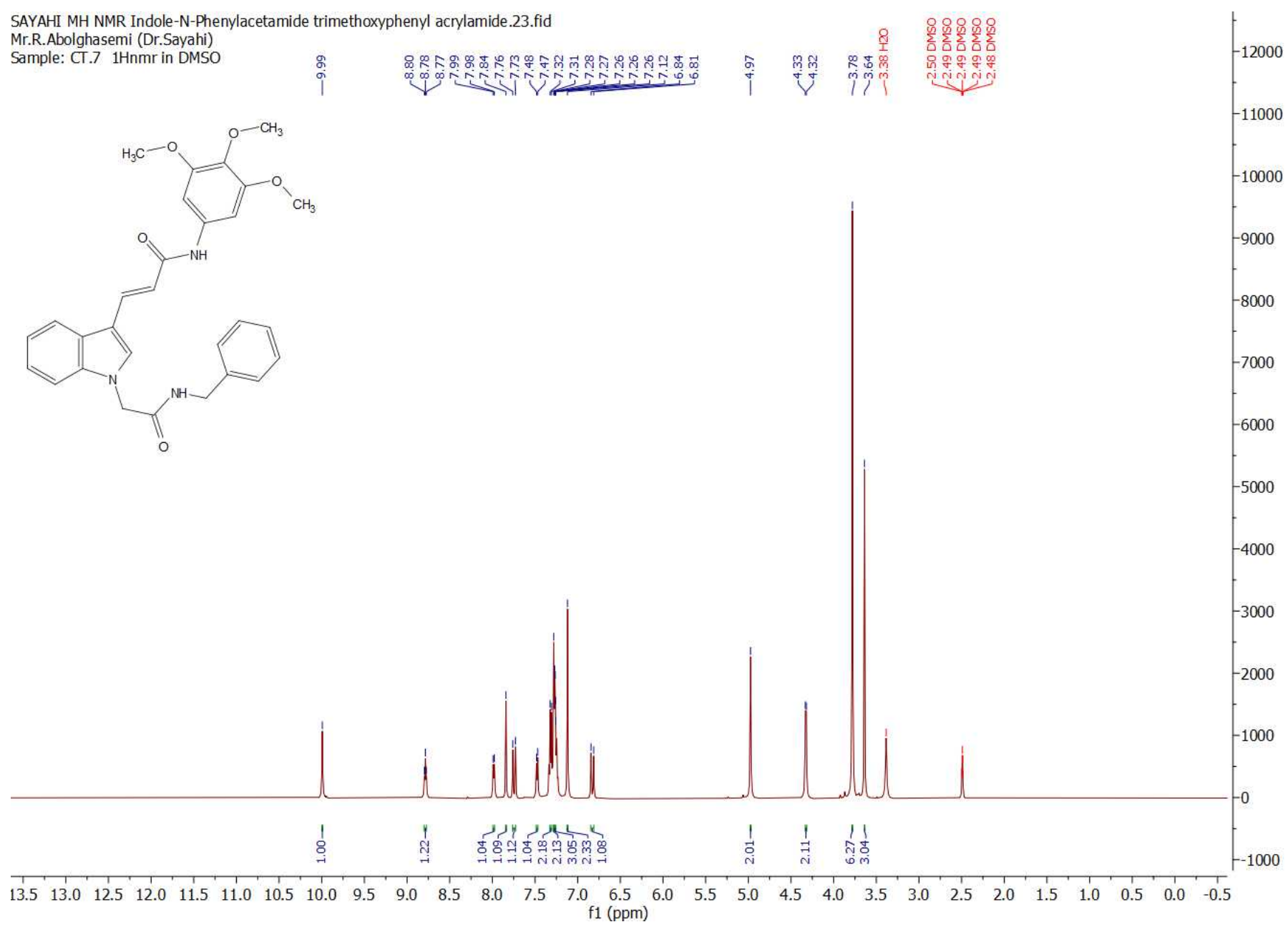

Figure S46. [13]*C{*[1]*H} NMR (126 MHz, DMSO-d*$_6$*) spectrum of* ***10n***

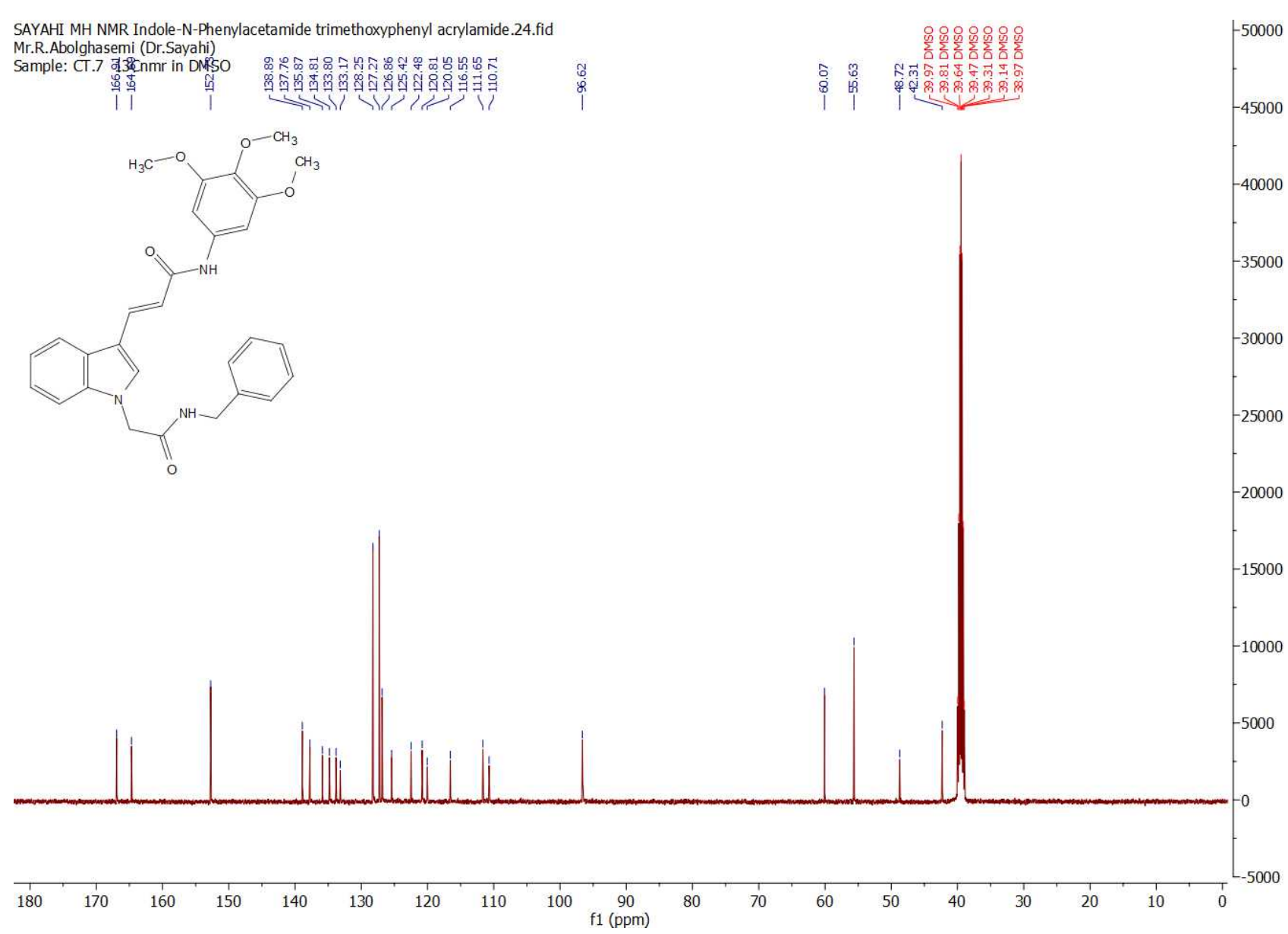

Figure S47. IR Spectral Data (ATR-FTIR) of ***10n***

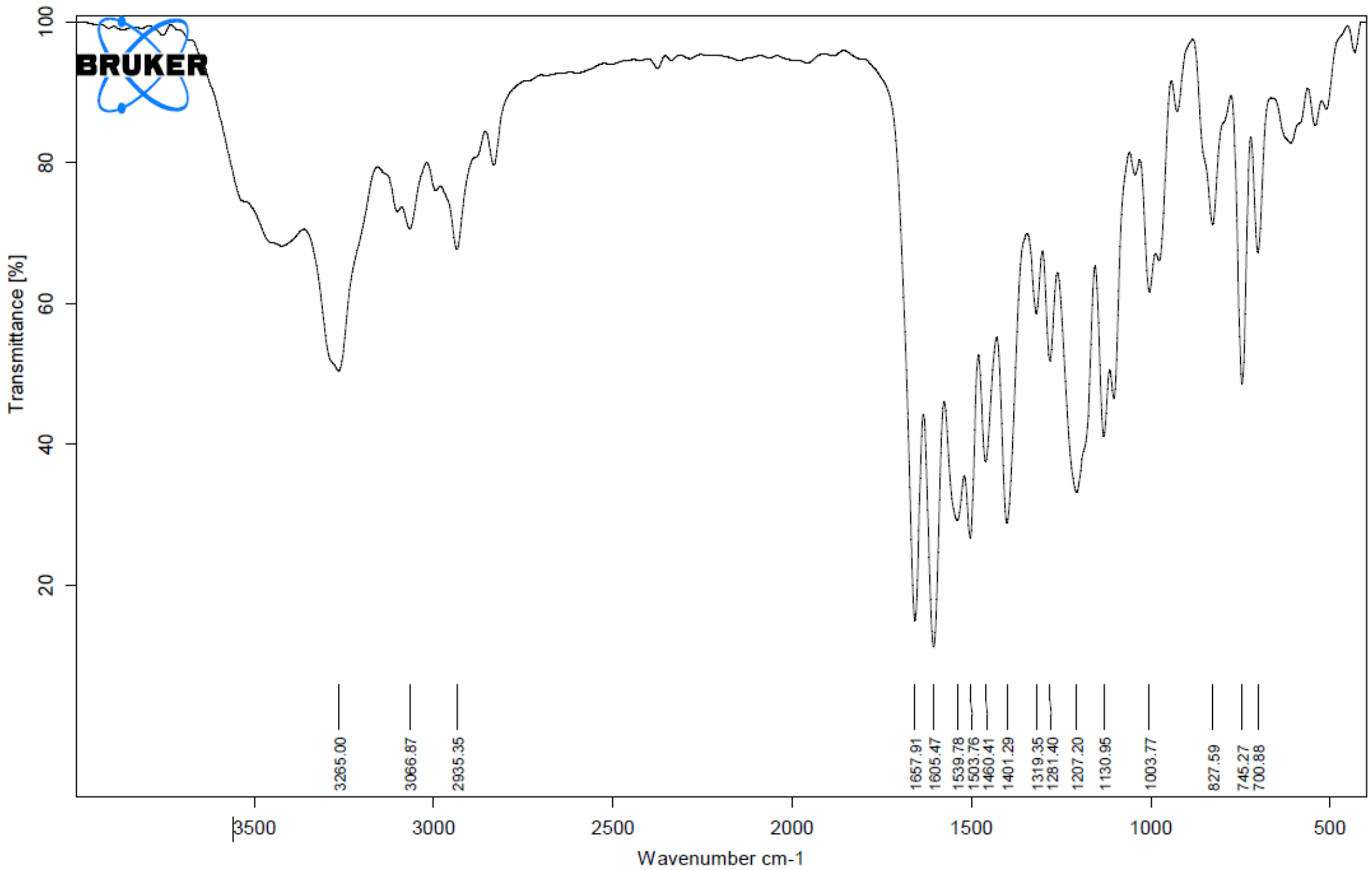

Figure S48. ¹H NMR (500 MHz, DMSO-d$_6$) spectrum of ***100***

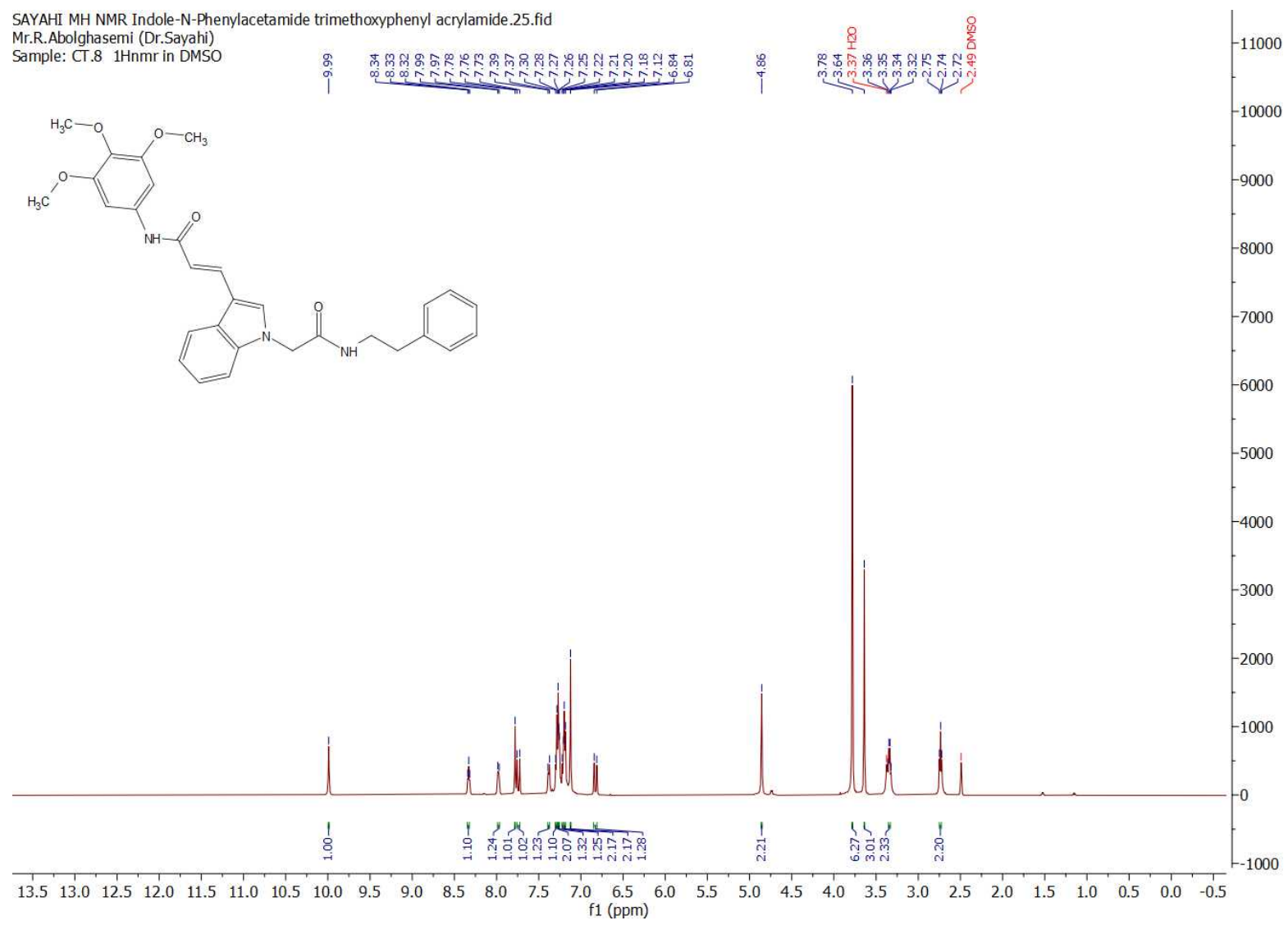

Figure S49. ¹³C{¹H} NMR (126 MHz, DMSO-d$_6$) spectrum of ***100***

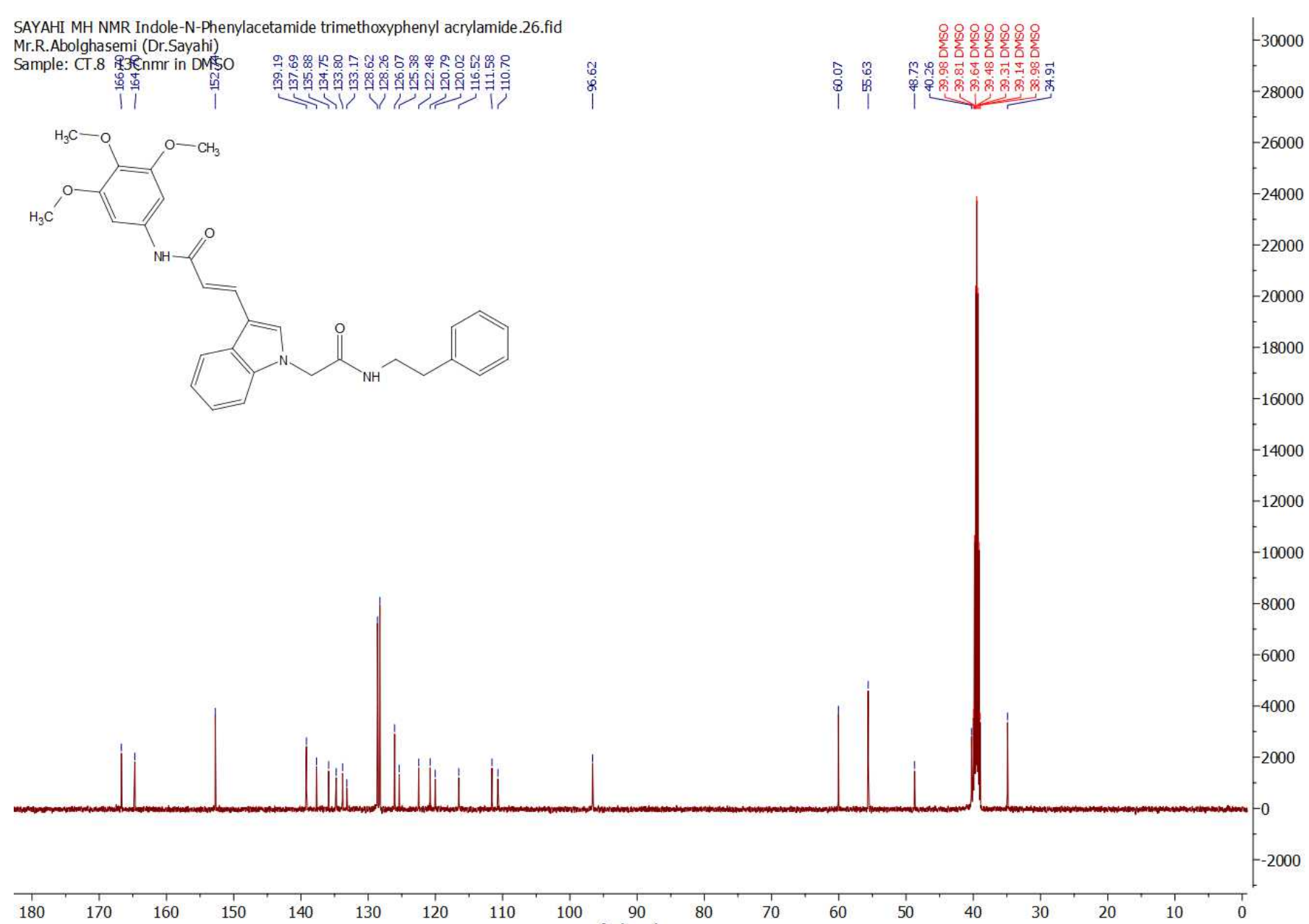

Figure S50. IR spectrum of ***100***

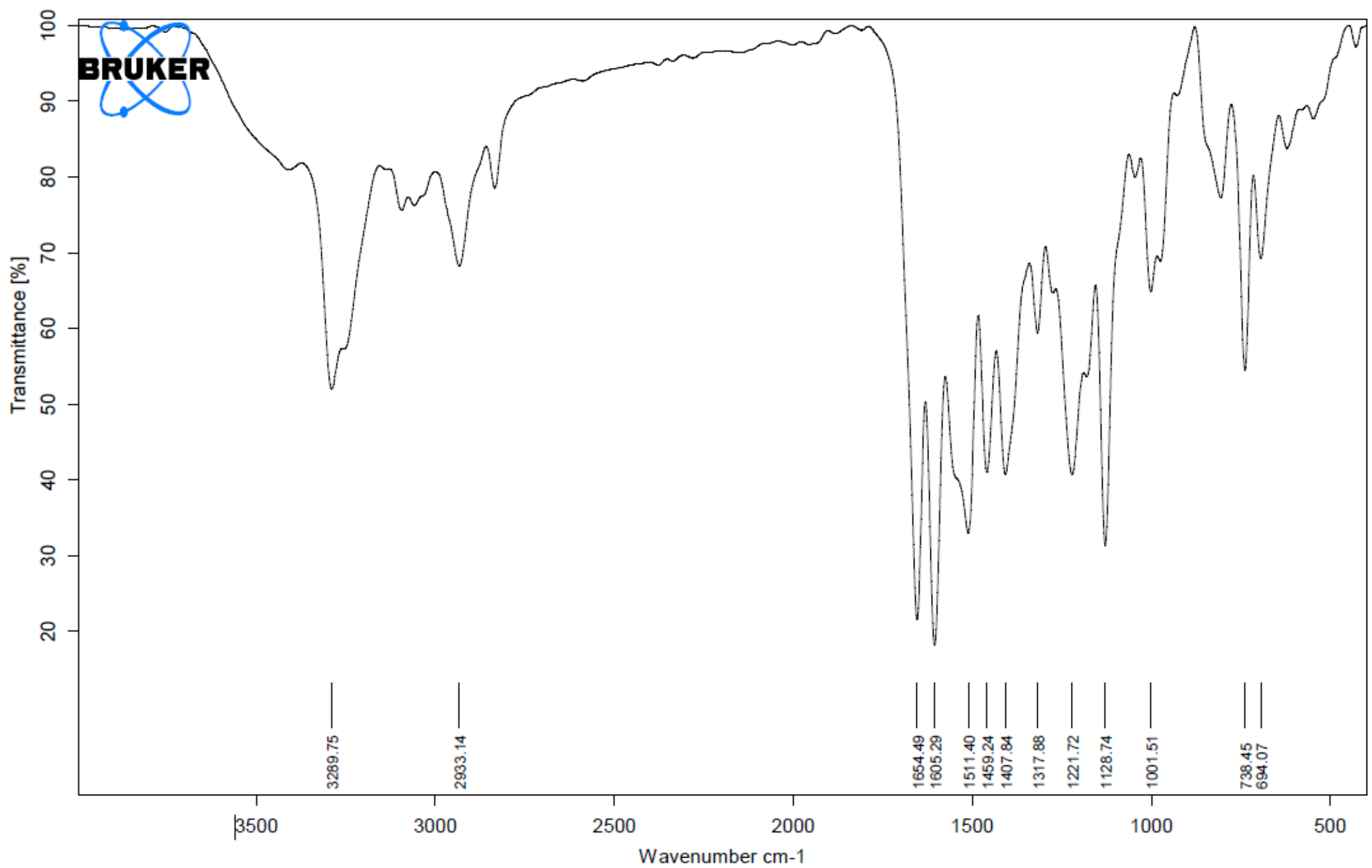

Fig. S51. Apoptosis induction in MCF-7 cancer cells by **10b** after 24 hours

Control **10b-15 μM**

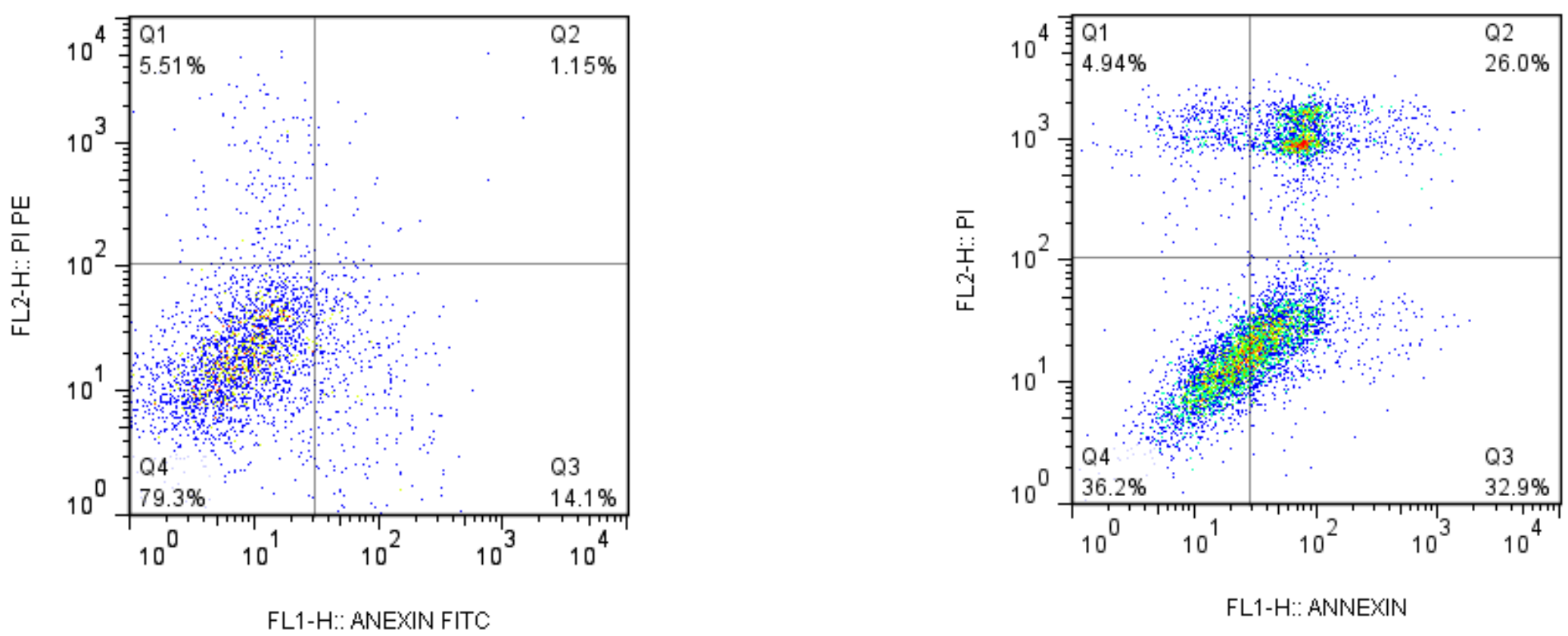

Fig. S52. Apoptosis induction in MCF-7 cancer cells by **10b** after 72 hours

10b-15 μM **10b-50 μM**

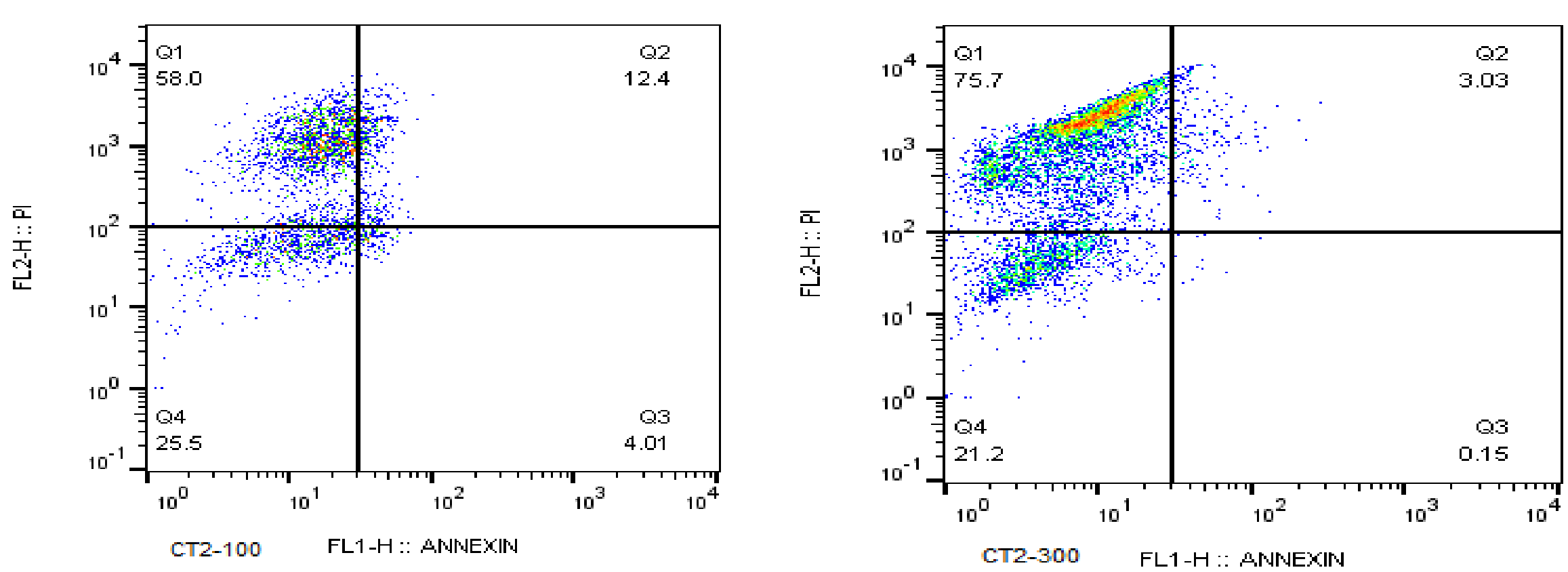

Results and discussion of *in silico* studies

Molecular docking was performed for the most potent analog against tubulin (PDB ID: 4O2B). This target selection correlates with the biological findings, as disruption of microtubule dynamics is known to induce cell-cycle alterations (indirect S/G2 phase effects), mitotic catastrophe, and, at higher concentrations, necrotic cell death. Figure S53 illustrates reported tubulin inhibitors, where the indole core and the trimethoxy aniline pendant contribute to binding at the colchicine site of tubulin [1-5]. Recently, new indole-based tubulin inhibitors have also been reported.[5, 6] The colchicine binding pocket is located at the intra-dimer interface of α- and β-tubulin and is functionally dominated by β-tubulin.

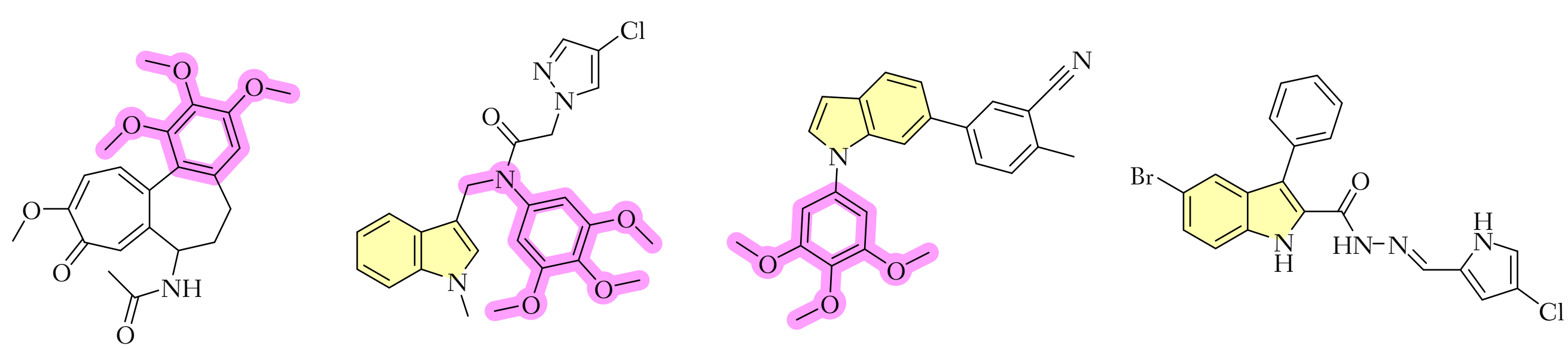

Colchicine | IC_{50}= 0.34 μM against MCF-7 | IC_{50}= 2.94 μM against MCF-7 | IC_{50}= 2.4 μM against T47D

Fig. S53. Recently reported tubulin inhibitors

To validate the reliability of the molecular docking protocol, redocking of the co-crystallized colchicine ligand into the colchicine-binding site of tubulin was performed. The reproduced binding pose showed a low root-mean-square deviation (RMSD = 1.27 Å) compared with the experimentally determined crystallographic conformation, confirming the accuracy and robustness of the docking procedure (Fig. S54).

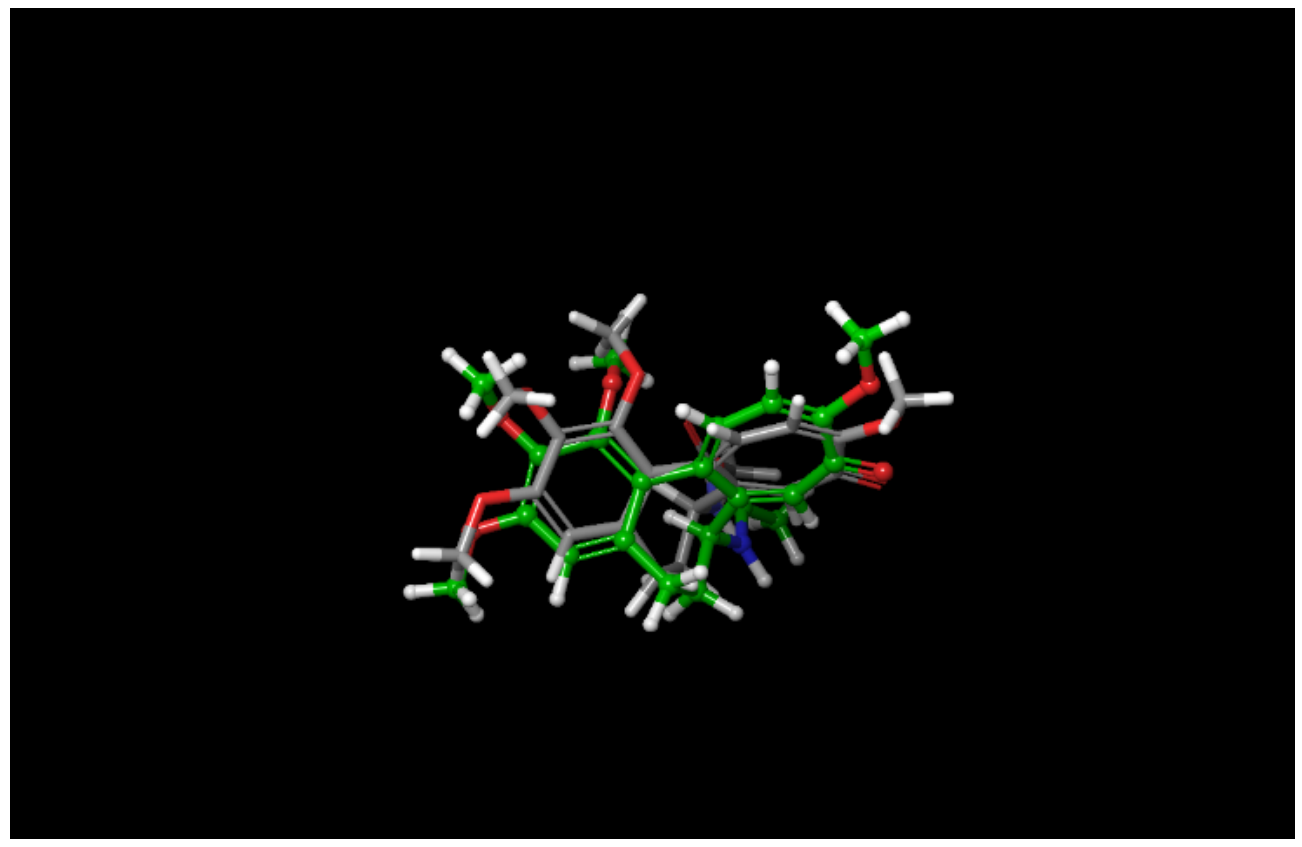

Fig. S54. Superposition of the crystallographic ligand and the docked ligand in the binding site.

The interaction pattern of the redocked colchicine was also consistent with the reported binding mode within the colchicine-binding pocket. Specifically, colchicine formed hydrogen-bond interactions with Val181 (2.02 Å, chain C) and Cys241 (3.19Å, chain D) residues, which are considered important residues within the colchicine-binding site. These results confirm that the docking protocol was suitable for subsequent evaluation of **10b (Fig. S55)**.

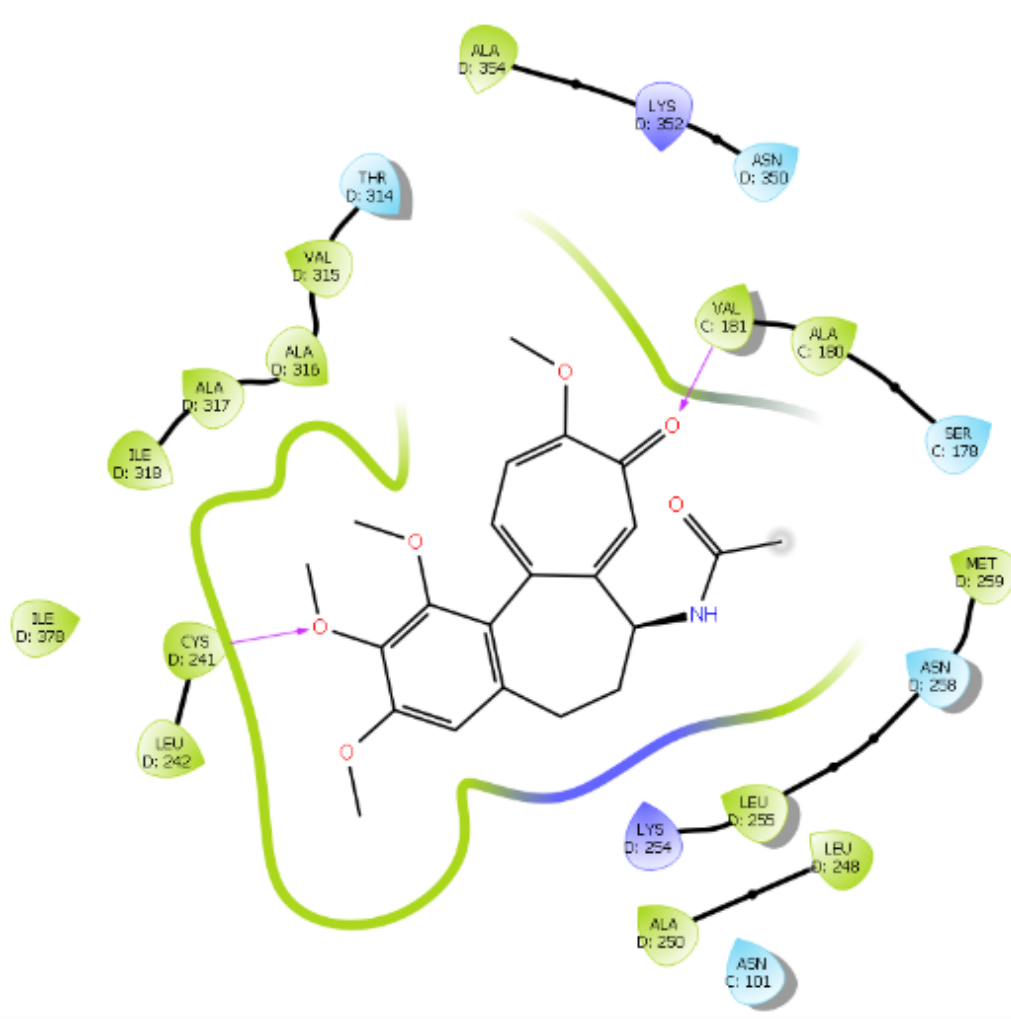

Fig. S55. 2D interaction of colchicine within the tubulin colchicine binding site (PDB ID: 4O2B).

Given the similarity of the synthesized derivatives to reported tubulin inhibitors, compound **10b** was docked into the tubulin-colchicine crystal structure as a suggested possible target, yielding a docking score of -8.8 kcal/mol. Detailed interaction analysis showed that the 3,4,5-trimethoxyphenyl moiety formed two H-bond interactions with Ser140. In addition, the amide linker attached to this aromatic ring established another H-bond with Glu183.

On the other side of the molecule, the *o*-tolyl acetamide group also participated in an H-bond interaction with Cys241, stabilizing the ligand within the colchicine binding pocket (Fig. S56).

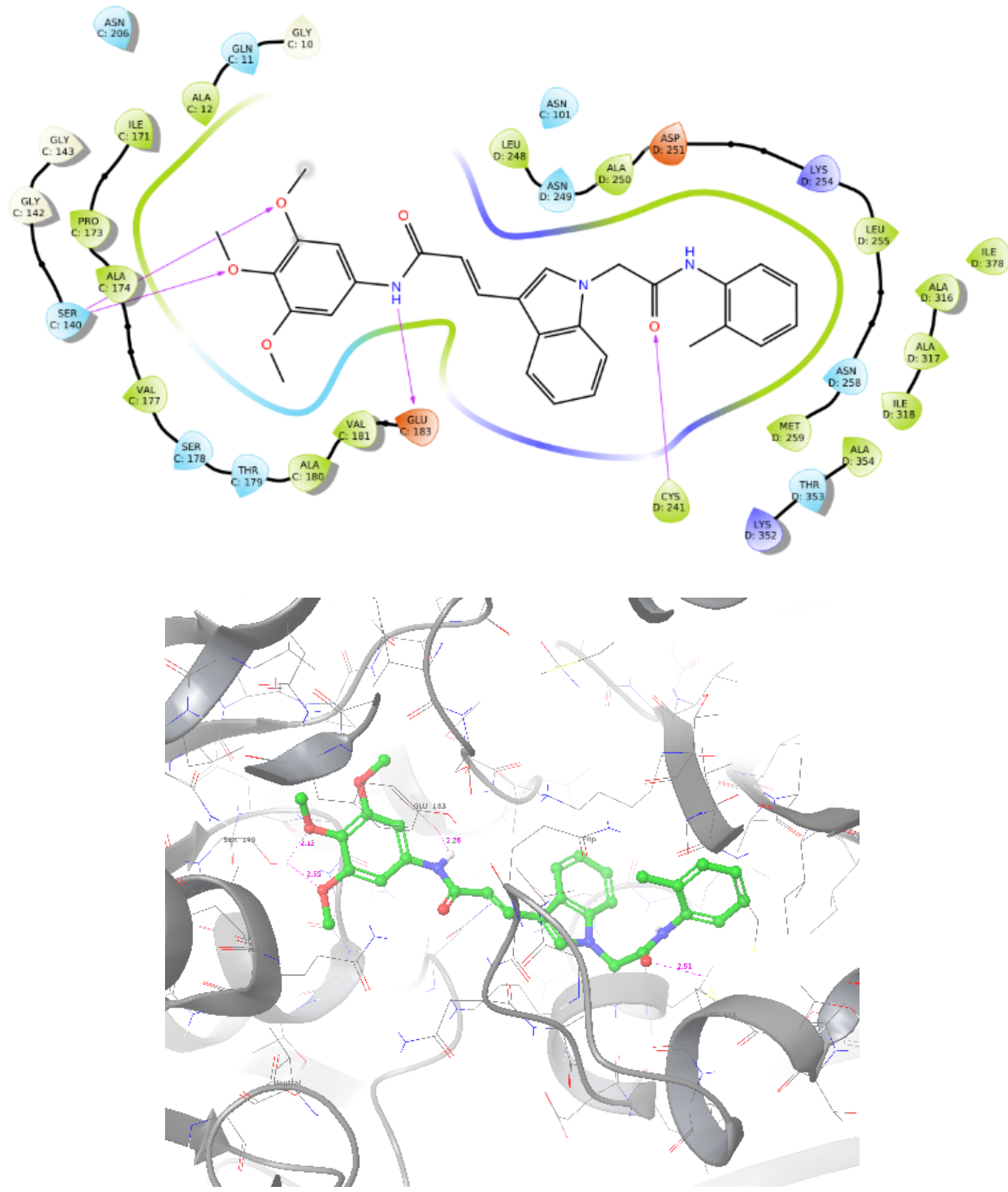

Fig. S56. 2D and 3D interaction diagrams of compound **10b** within the tubulin colchicine binding site (PDB ID: 4O2B).

Additionally, molecular docking analysis was performed for the less active analog **10i** to further investigate whether differences in biological activity could be explained by variations in the predicted binding interactions. The top-ranked docking pose of **10i** exhibited a predicted docking score of -6.58 kcal/mol within the colchicine-binding site. The 3,4,5-trimethoxyphenyl moiety established a hydrogen-bond interaction with Asn18, while the amide linker connecting the aromatic moiety to the benzimidazole scaffold formed an additional hydrogen bond with Thr225. Furthermore, the aromatic ring system participated in hydrophobic interactions with residues lining the binding cavity, contributing to stabilization of the ligand-protein complex. Although compound **10i** showed favorable predicted interactions, its weaker experimental cytotoxic activity relative to the lead compound suggests that docking scores and individual ligand-protein interactions alone are insufficient to fully explain biological

potency. These findings highlight the importance of considering additional factors, including cellular permeability, target engagement, and pharmacodynamic effects, when interpreting the relationship between docking predictions and biological activity(Fig. S57).

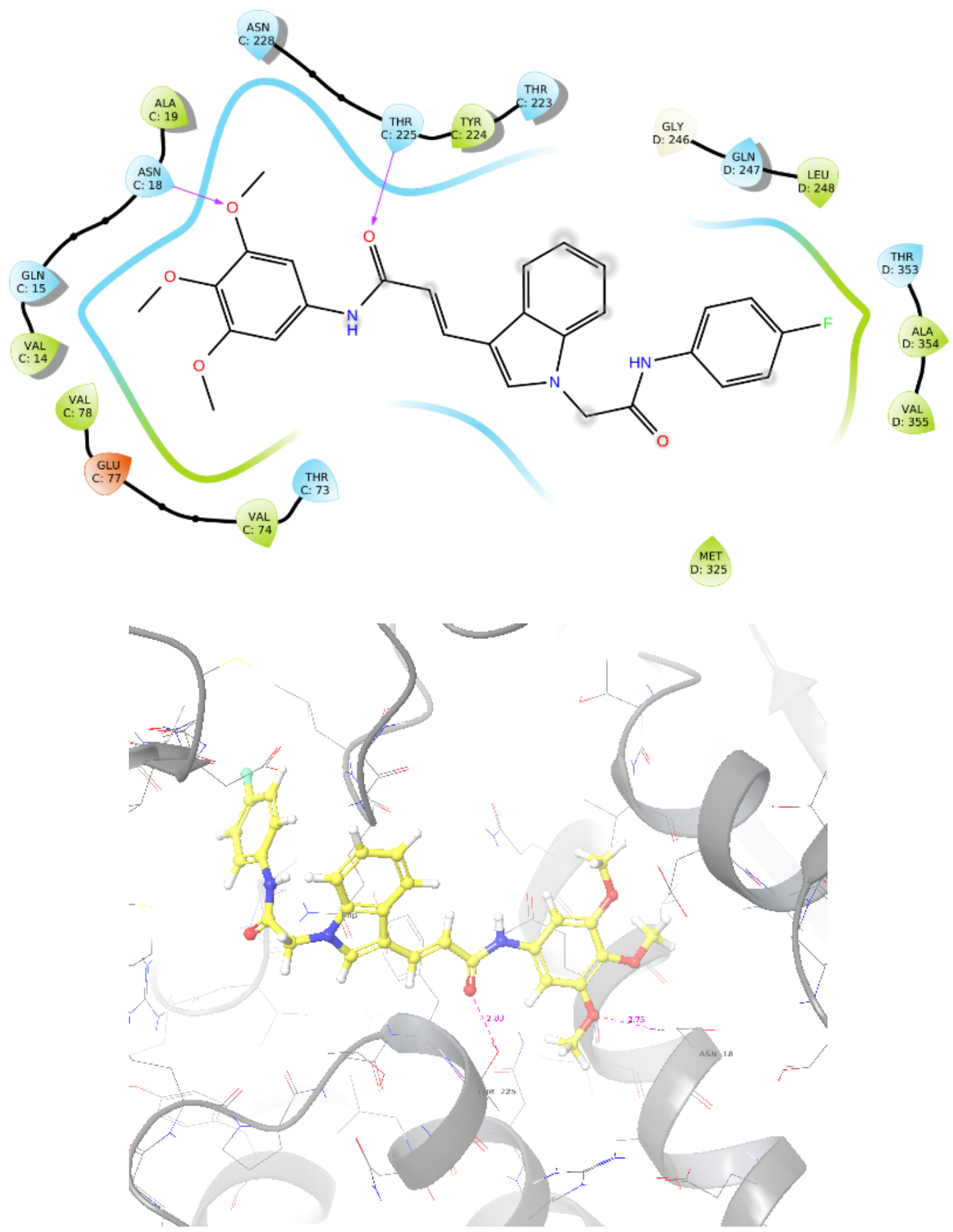

Fig. S57. 2D and 3D interaction diagrams of compound **10i** within the tubulin colchicine binding site (PDB ID: 4O2B).

To evaluate the binding stability of compound **10b** within the colchicine binding site of tubulin, a 100 ns molecular dynamics simulation was performed and compared with the apo tubulin system. The root mean square deviation (RMSD) profiles of the Cα atoms are presented in Fig. S58. The apo tubulin system reached fast equilibrium with an average RMSD of around 1.5 Å throughout the remainder of the simulation. Similarly, the tubulin-**10b** complex exhibited fast stability, reaching a maximum of 1.25 Å. These results indicate that ligand binding does not disrupt the global structural stability of tubulin and that the complex remains well equilibrated during the simulation (Fig. S58).

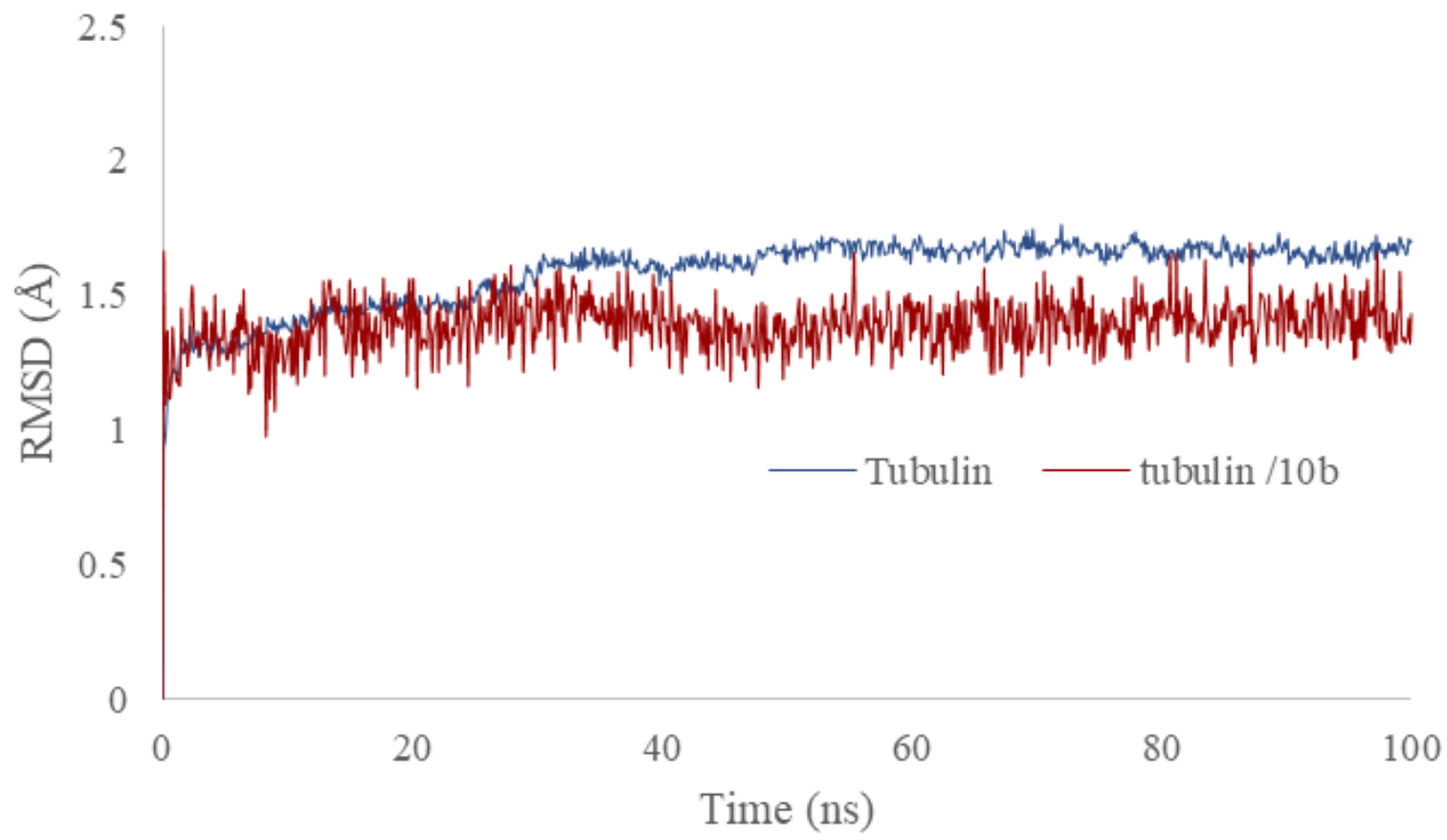

Fig. S58. The RMSD of the colchicine site of tubulin /**10b** (red), and the colchicine site of tubulin (blue) for over 100 ns MD simulation time

The RMSF analysis of the tubulin-**10b** complex is presented in Fig.S59 a. As shown, compound **10b** is well accommodated within the colchicine-binding pocket, where it is positioned between the α- and β-tubulin subunits and establishes stable interactions with residues from both chains throughout the simulation. Detailed interaction analysis (Fig.S59b) revealed that the indole acetamide moiety of **10b** formed two direct H-bonds with Asn249 and Lys254, along with an additional H-bond with Ser178. Additionally, the 3,4,5-trimethoxy aniline group formed two direct H-bond interactions with Gln11 and Ala12.

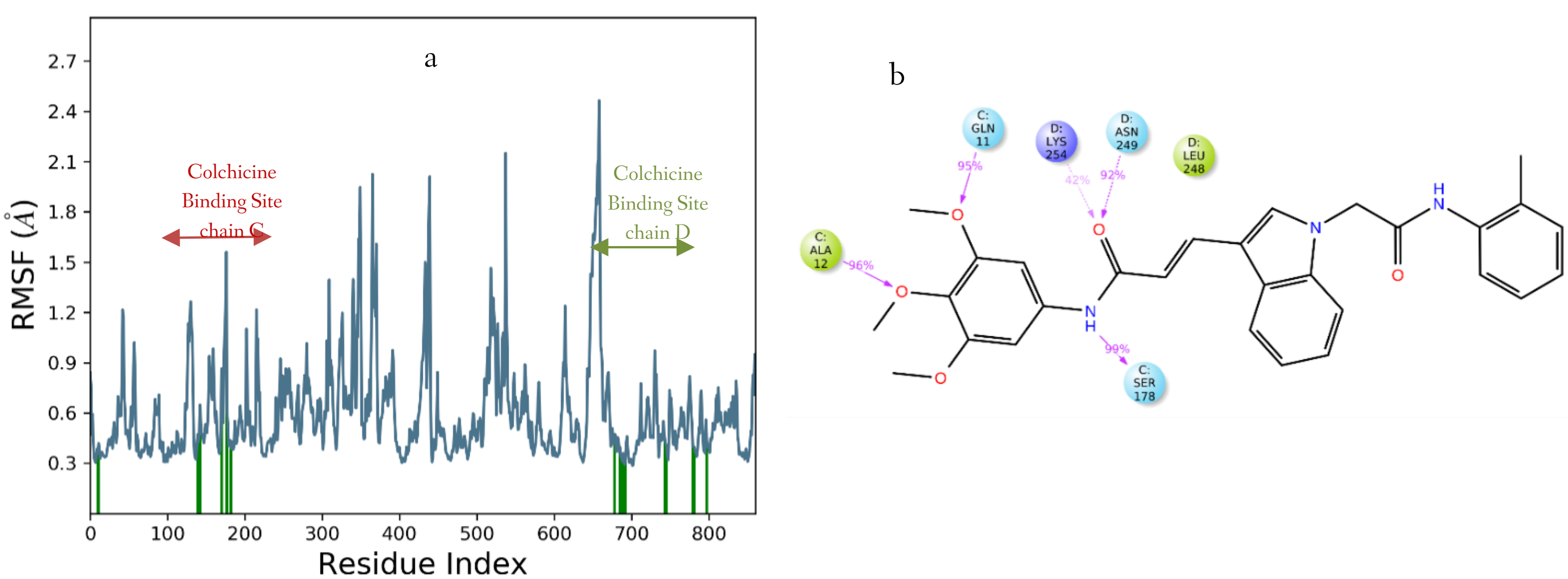

Fig. S59 a) RMSF graph of the Cα atoms of the colchicine site of tubulin /**10b** and b) representation of colchicine site of tubulin /**10b** interactions for over 40% of the simulation

Following this trend, the individual atomic fluctuations of the bound ligand were tracked over the 100 ns to further assess the stability of the ligand within the binding site. The ligand exhibited minimal fluctuations within the binding site, as indicated by RMSF values. All atoms showed RMSF values strictly at or below 1 Å. This restricted atomic fluctuation profile, together with hydrogen-bond interactions showing greater than 40% occupancy with residues α-Ser178, β-Asn249, and β-Lys254, supports the stability of **10b** within the colchicine-binding site during the simulation (Fig.S60).

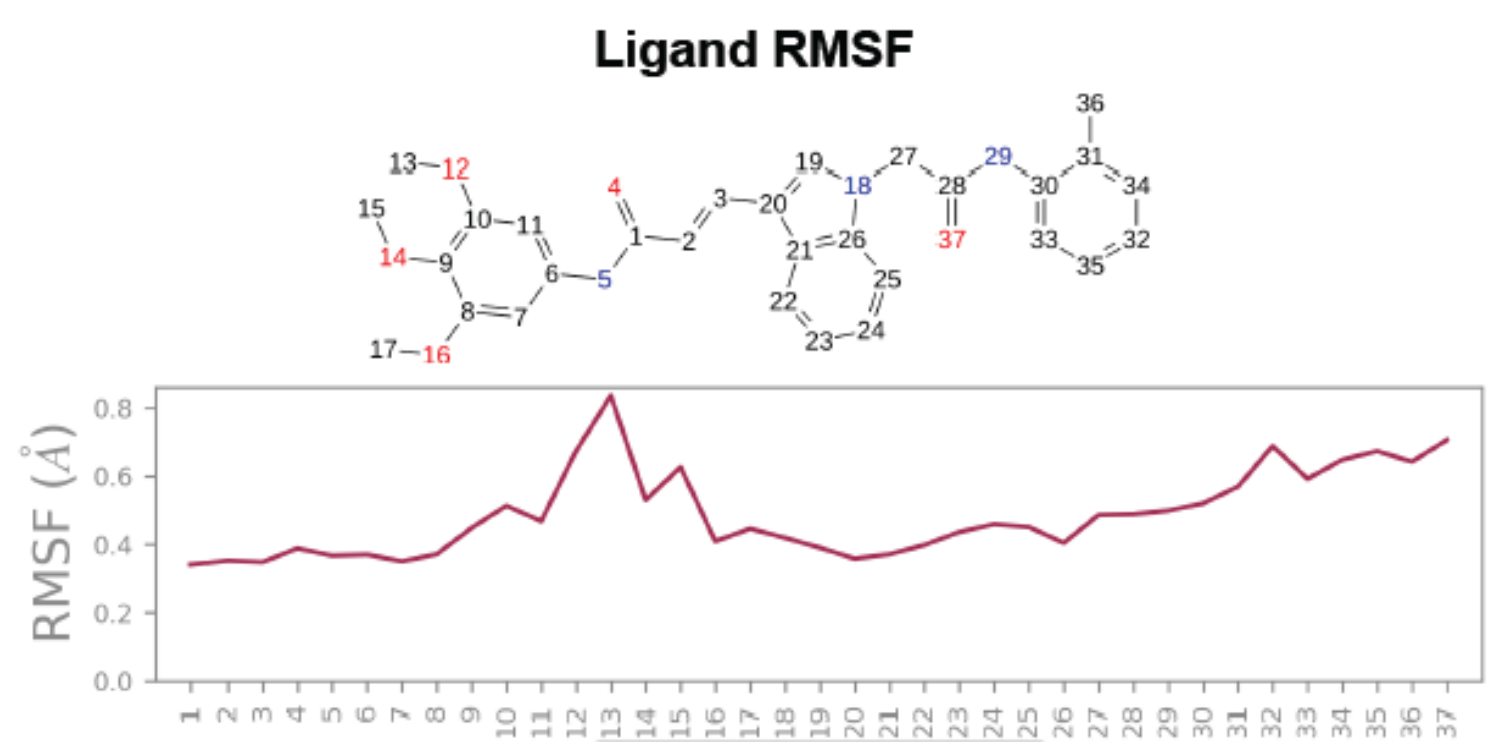

Fig. S60. RMSF of **10b** during the MD simulation

Method of molecular docking and molecular dynamics simulation

Molecular docking studies were performed using the Maestro molecular modeling platform (version 10.5, Schrödinger, LLC, New York, NY, USA). The X-ray crystal structure of the tubulin-colchicine complex (PDB ID: 4O2B) was obtained from the RCSB Protein Data Bank. Only chains C and D of the tubulin heterodimer were retained for docking studies. Protein preparation was carried out using the Protein Preparation Wizard with OPLS4 force field, and crystallographic water molecules were removed during system preparation. Ligand **10b** was parameterized using the OPLS4 force field following LigPrep preparation, with relevant protonation and tautomeric states generated at pH 7.0. The co-crystallized colchicine ligand was used to define the binding site and subsequently removed before docking, whereas the bound GDP/GTP molecules and associated Mg^{2}⁺ ions were retained to preserve the native conformation of the protein. Missing loops and side chains were reconstructed using Prime. Protein protonation states were assigned at pH 7.0 using PROPKA, while ligand protonation states, metal-binding states, and the most probable tautomeric forms were generated using Epik within LigPrep at the same pH. An induced-fit docking protocol was employed. The receptor grid was centered on the centroid of the

co-crystallized colchicine ligand, and the grid dimensions were set to 20 Å to encompass the entire colchicine-binding pocket and neighboring residues. Residues within 8 Å of the binding site were refined during induced-fit docking. Multiple binding poses were generated, and the top-ranked pose with the most favorable docking score was selected for subsequent interaction analysis.

The best pose of the induced-fit docking of the colchicine site of tubulin was selected for molecular dynamics simulation using Desmond v5.3 from Schrödinger's suite Maestro. The **10b**-tubulin complex was solvated with explicit SPC water molecules and positioned at the center of an orthorhombic box under periodic boundary conditions, ensuring a minimum buffer distance of 10 Å between the protein surface and the edges of the simulation box. Counterions from a 0.15 M NaCl solution were added to neutralize the system and achieve a total ionic strength of 0.15 M, mimicking real cellular ionic concentrations. The MD protocol consisted of a structured relaxation sequence followed by production runs. The system was first relaxed by performing energy minimization using the steepest-descent approach for 2500 steps with a solute harmonic positional restraint of 50 kcal/mol, followed by a second unrestrained minimization stage to clear local steric clashes. To prevent abrupt changes, a gradual thermal conditioning protocol was implemented where the system temperature gradually increased from 0 to 300 K over a 12 ps trajectory under a constant-volume (NVT) ensemble, maintaining positional restraints on the solute heavy atoms. System density was subsequently equilibrated via a 12 ps constant pressure (NPT) run at 300 K and 1.01325 bar with remaining restraints, followed by a final 24 ps unrestrained NPT equilibration phase.

The explicit production MD simulations were conducted under the NPT ensemble, maintaining a constant number of atoms, pressure (1.01325 bar), and temperature (300 K) for a total continuous production timeline of 100 ns. An integration timestep of 2.0 fs was set throughout the trajectory. Temperature was achieved utilizing the Nose-Hoover chain thermostat (1 ps), and pressure was controlled via the Martyna-Tobias-Klein barostat with a relation time of 2 ps. Long-range electrostatic interactions were computed using the smooth Particle Mesh Ewald (PME) method with a grid spacing of 0.8 Å, while non-bonded van der Waals and short-range electrostatic cut-offs were smoothly truncated at a radius of 9.0 Å.

Reference:

1. Saruengkhanphasit, R.; Chatwichien, J.; Ngiwsara, L.; Lirdprapamongkol, K.; Niwetmarin, W.; Eurtivong, C.; Kittakoop, P.; Svasti, J.; Ruchirawat, S., Design, synthesis, in silico and in vitro evaluation of pyrrole–indole hybrids as dual tubulin and aromatase inhibitors with potent anticancer activities. *RSC advances* **2025,** *15* (27), 21962-21976.
2. Venettozzi, M.; Coburn, T. E.; Evans, B. A.; Grillo, M. S.; Le, A. N.; Minayev, A. M.; Muse, A. H.; Otchere, J. G.; Potvin, K. L.; Staunton, A. P., Exploring Regioisomeric Indole–Furanone Tubulin Inhibitors. *ACS omega* **2025,** *10* (38), 44675-44682.
3. Gu, Y.; Gai, C.; Zou, S.; Song, Y.; Zhang, J.; Zhao, Q.; Chai, X.; Wang, P., Design, Synthesis and Anticancer Activity of 6-Substituted-1-(3, 4, 5-trimethoxyphenyl)-1H-indole Against Tubulin Polymerisation. *Molecules (Basel, Switzerland)* **2025,** *30* (23), 4538.

4. Mahmoud, E.; Abdelhamid, D.; Mohammed, A. F.; Almarhoon, Z. M.; Bräse, S.; Youssif, B. G.; Hayallah, A. M.; Abdel-Aziz, M., Design, synthesis, and antiproliferative activity of novel indole/1, 2, 4-triazole hybrids as tubulin polymerization inhibitors. *Pharmaceuticals* **2025,** *18* (2), 275.
5. Alsfouk, A. A.; Nossier, E. S.; Omar, M. A.; Mounier, M. M.; Mohamed, H. S.; Sarhan, A. E.; Saleh, A.; Srour, A. M., Indole-based carbohydrazides as potent anti-proliferative leads and tubulin polymerization inhibitors: design, synthesis, cytotoxic evaluation, in silico ADMET and docking studies. *Future Med Chem* **2026,** *18* (15), 2015-2029.
6. Abba, A.; Unal, N.; Dede, Z. S.; Bedir, I.; Telci, D.; Aru, B.; Demirel, G. Y.; Akdeniz, G. Y.; Timuçin, A. C.; Gungor, B.; Akgün, H., Indole ring linked thiazolidone hybrid molecules as potent tubulin inhibitors. *Letters in Drug Design & Discovery* **2025,** *22* (12), 100367.
